# Supplementary material for: Inpatient midwifery staffing levels and postpartum readmissions: a retrospective multicentre longitudinal study
Source: BMJ Open. 2024 Apr 3;14(4):e077710. doi: 10.1136/bmjopen-2023-077710 (PMC11146407; doi:10.1136/bmjopen-2023-077710)

SUPPLEMENTARY INFORMATION

Table of contents

S1 Model fit and missing data 2
1.1 Model fit by Akaike information criterion (AIC) and Bayesian information criterion (BIC) .. 2
1.2 Collinearity testing 2
1.3 Missing data per person in the study population (women who had a birth admission) 2
S2 Diagnosis codes for the 2903 postpartum readmissions 3
S3 Staffing levels expressed as Individuals per Registered Midwife (RM) and Maternity Assistant (MA) 5
S4 Staffing on day and night shifts over time in each of the Trusts 6
S5 Skill mix of staff within the three Trusts 7
S6 Trends in staffing over time in each of the Trusts 8
S7 Mean admissions per day and night in each Trust 9
S8 Univariable analyses 10
S8.1 Age category 10
S8.2 Mode of birth 11
S8.3 SHMI risk 12
S8.4 Understaffing by Registered Midwives, Maternity Assistants and Overall staffing 12
S8.5 Turnover (admissions + discharges) more than expected (mean) value for Trust 12
S8.6 Skill mix lower than expected (mean) levels for service 13
S9 Full models for staffing below the mean compared to mean staffing (HPPD) 14
S10. Full models with staffing expressed as individuals per midwife/maternity assistant (absolute level) 15
S11 Additional analysis : High and low staffing categories compared with mean staffing 16
S12 Frequency of exposure to staffing categories in study population 17
S13 Results of full models by subset of population 18
S14 Flow chart for data selection 19

SUPPLEMENTARY INFORMATION

S1 Model fit and missing data

1.1 Model fit by Akaike information criterion (AIC) and Bayesian information criterion (BIC)

Postpartum readmission	7 days from discharge model		30 days from discharge model	
	AIC	BIC	AIC	BIC
Empty model nested in Trust	18036.62	18054.76	23539.27	23557.41
Age category added	18031.47	18058.68	23527.22	23554.43
Mode of birth added	17667.94	17703.96	23024.88	23060.91
RM<mean staffing added	17240.87	17285.81	22518.88	22563.81
MA<mean staffing added	17112.23	17166.03	22296.37	22350.17
SHMI added	17008.74	17071.47	22149.12	22211.84
Skill mix added (did not improve model fit so excluded from full model)	17010.53	17082.21	22148.77	22220.45
Turnover added (did not improve model fit so excluded from full model)	17012	17092.65	22149.99	22230.63

RM Registered Midwife, MA Maternity Assistant, SHMI Standardised Hospital Mortality Indicator

1.2 Collinearity testing

Test for collinearity using the Variance inflation factor (VIF)

	VIF
understaffRMless1	1.37
understaffMAless1	1.90
age_category	1.00
Mode_birth	1.00
SHMI_risk	1.00
Higher expected turnover	1.02
Less expected skill mix	1.56

RM Registered Midwife, MA Maternity Assistant

VIF scores are low (<2) so collinearity was not detected. A VIF indicating high collinearity would be >10.

1.3 Missing data per person in the study population (women who had a birth admission)

Age	None
Length of stay	None
Mode birth	None
SHMI	385/64,250 (0.6%)
Staffing Registered midwives	1,258/64,250 (1.96%)
Staffing Maternity Assistants	2,452/64,250 (3.82%)

(Maternity assistant staffing data was missing from Trust A after 28.1.2019)

Turner LY, *et al. BMJ Open* 2024; 14:e077710. doi: 10.1136/bmjopen-2023-077710

SUPPLEMENTARY INFORMATION

S2 Diagnosis codes for the 2903 postpartum readmissions

**ICD codes for primary diagnosis matched to description** Where categories are broad the second diagnosis code has been reported at the end of this table	Count	percentage
Total number of readmissions	**2903**	
Other complications of the puerperium, not elsewhere classified*	482	16.6%
Care and examination of lactating mother**	373	12.8%
Delayed and secondary postpartum haemorrhage	310	10.7%
Puerperal sepsis	246	8.5%
Infection of obstetric surgical wound	227	7.8%
Other specified diseases and conditions complicating pregnancy, childbirth and the puerperium***	159	5.5%
Disruption of caesarean section wound	104	3.6%
Disruption of perineal obstetric wound	103	3.5%
Maternal care for rhesus isoimmunization	71	2.4%
Nonpurulent mastitis associated with childbirth	63	2.2%
Unspecified maternal hypertension	57	2.0%
Haematoma of obstetric wound	45	1.6%
Urinary tract infection following delivery	43	1.5%
Persons encountering health services in other specified circumstances	40	1.4%
Pyrexia of unknown origin following delivery	39	1.3%
Fitting and adjustment of urinary device	33	1.1%
Anaemia complicating pregnancy, childbirth and the puerperium	30	1.0%
Diseases of the digestive system complicating pregnancy, childbirth and the puerperium	26	0.9%
Care and examination immediately after delivery	25	0.9%
Spinal and epidural anaesthesia-induced headache during the puerperium	24	0.8%
Other prophylactic chemotherapy	23	0.8%
Spinal and epidural anaesthesia-induced headache during labour and delivery	21	0.7%
Gestational [pregnancy-induced] hypertension	20	0.7%
Other infection of genital tract following delivery	17	0.6%
Prophylactic immunotherapy	16	0.6%
Other specified pregnancy-related conditions	15	0.5%
Other maternal infectious and parasitic diseases complicating pregnancy, childbirth and the puerperium	14	0.5%
Pre-eclampsia, unspecified	14	0.5%
Retained portions of placenta and membranes, without haemorrhage	13	0.4%
Routine postpartum follow-up	11	0.4%
Supervision of other normal pregnancy	11	0.4%
Other specified puerperal infections	9	0.3%
Diseases of the respiratory system complicating pregnancy, childbirth and the puerperium	8	0.3%
Unspecified infection of urinary tract in pregnancy	8	0.3%
Complication of the puerperium, unspecified	7	0.2%
Diseases of the circulatory system complicating pregnancy, childbirth and the puerperium	7	0.2%
Obstetric blood-clot embolism	7	0.2%
Attention to surgical dressings and sutures	6	0.2%
Elevated blood-pressure reading, without diagnosis of hypertension	6	0.2%
Examination and observation for other specified reasons	6	0.2%

SUPPLEMENTARY INFORMATION

**ICD codes for primary diagnosis matched to description**<br>Where categories are broad the second diagnosis code has been reported at the end of this table	**Count**	**percentage**
Mental disorders and diseases of the nervous system complicating pregnancy, childbirth and the puerperium	6	0.2%
Other immediate postpartum haemorrhage	6	0.2%
Maternal care due to uterine scar from previous surgery	5	0.2%
Other and unspecified disorders of breast associated with childbirth	5	0.2%
Postpartum coagulation defects	5	0.2%
Second degree perineal laceration during delivery	5	0.2%
Superficial thrombophlebitis in the puerperium	5	0.2%
Unspecified pre-existing hypertension complicating pregnancy, childbirth and the puerperium	5	0.2%
Other conditions with ICD codes affecting <5 women in dataset each (condensed for reporting in this table)	115	4.0%
Blank	7	0.2%

* 68/478 had blank second diagnosis code, 179/478 had ICD indicating a type of pain as second diagnosis code
** 226/373 had blank second diagnosis code, 125/373 had 'Healthy person accompanying sick person' as second diagnosis code
***24/159 had blank second diagnosis code, no pattern seen in remaining ICD codes

When totalling the above 677/2903 (23.3%) of postpartum readmissions were due to infection or sepsis, 316/2903 (10.9%) were due to haemorrhage

Missing ICD diagnosis code for readmissions 7/2903 (0.2%)

Turner LY, *et al. BMJ Open* 2024; 14:e077710. doi: 10.1136/bmjopen-2023-077710

SUPPLEMENTARY INFORMATION

S3 Staffing levels expressed as Individuals per Registered Midwife (RM) and Maternity Assistant (MA)

Trust	Mean RM HPPD	Individuals per Midwife	Mean MA HPPD	Individuals per Maternity Assistant
A	11.747	2.330	4.869	5.934
B	5.940	4.104	1.759	14.205
C	6.906	3.576	2.650	9.387
All Trusts (non weighted average)	6.520	3.879	2.146	12.67

RM Registered Midwife, MA Maternity Assistant, HPPD= Hours Per Patient Day

Staffing was calculated for each Trust in the study period and averaged per day.
The ‘individuals cared for per staff member’ was calculated using the following formula = 24/HPPD.

Turner LY, *et al. BMJ Open* 2024; 14:e077710. doi: 10.1136/bmjopen-2023-077710

SUPPLEMENTARY INFORMATION

S4 Staffing on day and night shifts over time in each of the Trusts

Data for staffing for whole admitted population during specified time periods

Year	DAY or NIGHT	Mean RM HPPD	Mean MA HPPD	Mean Overall HPPD
Trust A				
2015	Day	10.75	4.56	15.32
2015	Night	11.10	3.66	14.77
2016	Day	11.73	5.47	17.20
2016	Night	11.76	5.18	16.94
2017	Day	14.22	6.04	20.26
2017	Night	13.54	5.46	19.00
2018	Day	14.08	5.97	20.04
2018	Night	13.81	5.09	18.90
2019	Day	14.68	5.77	19.06
2019	Night	14.28	4.84	17.96
2020	Day	12.57	Not available	Not available
2020	Night	12.44	Not available	Not available
Trust B				
2015	Day	7.00	1.82	8.83
2015	Night	5.09	1.52	6.60
2016	Day	6.93	1.86	8.80
2016	Night	4.99	1.53	6.52
2017	Day	7.19	2.08	9.27
2017	Night	5.04	1.65	6.70
2018	Day	6.71	1.81	8.52
2018	Night	4.81	1.34	6.15
2019	Day	6.92	2.15	9.07
2019	Night	4.89	1.72	6.61
2020	Day	6.61	2.35	8.96
2020	Night	4.84	2.09	6.93
Trust C				
2015	Day	7.05	1.98	9.03
2015	Night	5.82	2.01	7.83
2016	Day	7.02	2.17	9.19
2016	Night	5.91	2.12	8.03
2017	Day	7.23	2.52	9.75
2017	Night	6.06	2.37	8.44
2018	Day	7.34	2.76	10.10
2018	Night	6.16	2.64	8.80
2019	Day	7.91	2.84	10.76
2019	Night	6.96	2.85	9.81
2020	Day	8.62	3.25	11.87
2020	Night	7.84	3.10	10.94

RM = registered midwife MA=maternity assistant HPPD= Hours Per Patient Day

Turner LY, *et al. BMJ Open* 2024; 14:e077710. doi: 10.1136/bmjopen-2023-077710

SUPPLEMENTARY INFORMATION

S5 Skill mix of staff within the three Trusts

This table indicates the skill mix and seniority of staff within Registered Midwife and Maternity Assistant groups.

	Sum of worked hours and % of total in each staff group					
	Total sum of worked hrs	RM Band 6 or above	Registered midwives	Registered nurses	Band 3 care assistants	Band 2 care assistants
Trust A*	316,880	33.5%	70.7%	0.09%	2.2%	26.8%
Trust B	1,990,058	60.4%	77.4%	0%	3.5%	19.1%
Trust C	933,846	59.8%	73.4% (nurses not listed separately)		7.3%	19.2%

*up to 28.1.2019 as care assistant unavailable after this date

The bands refer to NHS Employers Agenda for Change pay scales.
Band 3 is a senior maternity assistant, Band 6 is a senior registered midwife.

Turner LY, *et al. BMJ Open* 2024; 14:e077710. doi: 10.1136/bmjopen-2023-077710

SUPPLEMENTARY INFORMATION

S6 Trends in staffing over time in each of the Trusts

RM = registered midwife MA=maternity assistant HPPD= Hours Per Patient Day

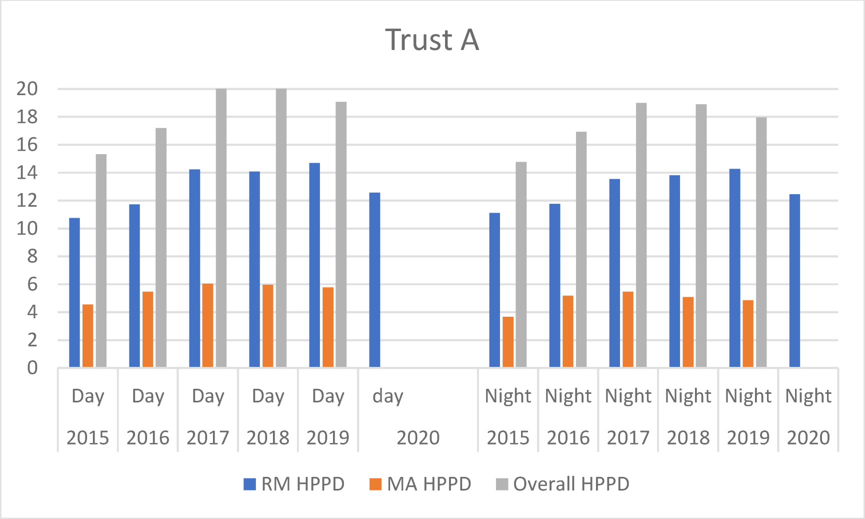

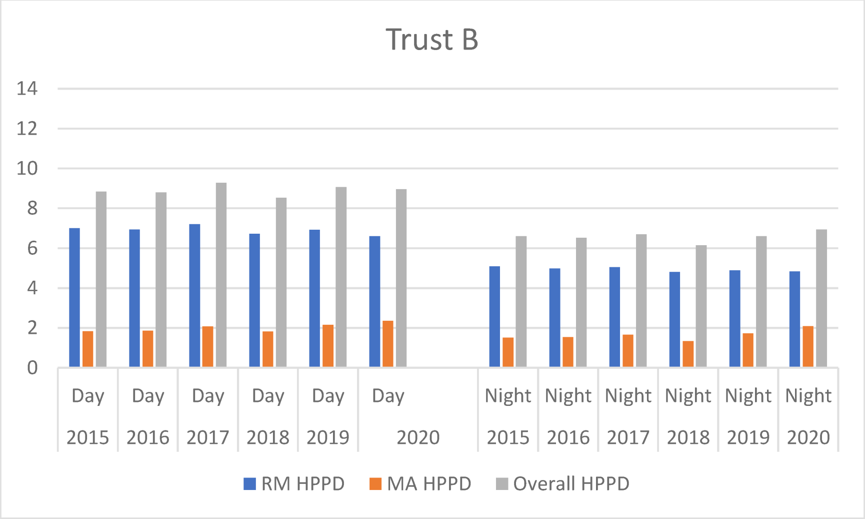

Turner LY, et al. BMJ Open 2024; 14:e077710. doi: 10.1136/bmjopen-2023-077710

SUPPLEMENTARY INFORMATION

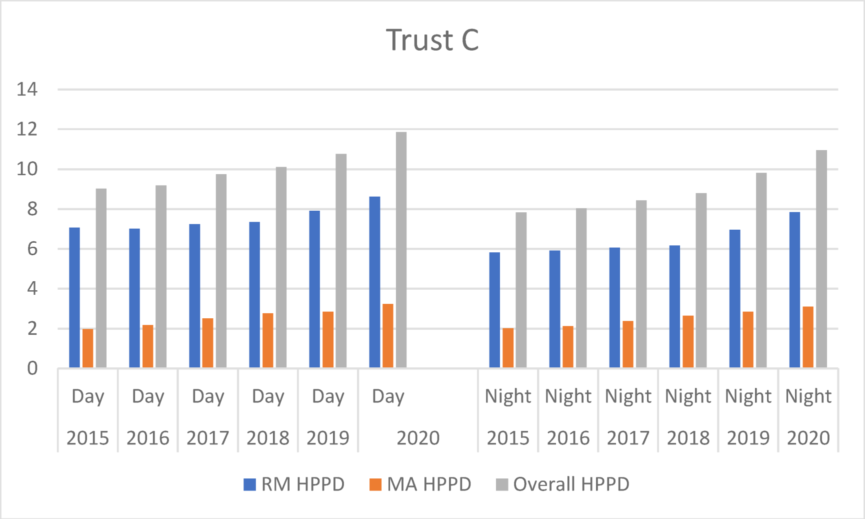

S7 Mean admissions per day and night in each Trust

	Mean admissions per Day	Mean admissions per Night
Trust A	3.2	1.7
Trust B	39.0	28.4
Trust C	15.6	13.5

Turner LY, *et al. BMJ Open* 2024; 14:e077710. doi: 10.1136/bmjopen-2023-077710

SUPPLEMENTARY INFORMATION

S8 Univariable analyses

Based on dataset with 64,250 records. Cases leading to birth only and their exposure to staffing and outcomes. Each independent variable was entered into a regression model with only this variable and the readmission outcome variable, nested within hospital Trust.

S8.1 Age category

For outcome of readmission within 7 days
(other age categories are compared with the 20-24 age group)

	Readmitted within 7 days		
	OR	95% CI	
Age category			
<20 years	1.009	0.770	1.323
20-24 years	1.000	reference category	
25-29 years	0.954	0.828	1.100
30-34 years	1.040	0.906	1.195
35-39 years	1.043	0.893	1.219
40+ years	1.476	1.193	1.827

For outcome of readmission within 30 days
(other age categories are compared with the 20-24 age group)

	Readmitted within 30 days		
	OR	95% CI	
Age category			
<20 years	0.994	0.787	1.256
20-24 years	1.000	reference category	
25-29 years	1.013	0.897	1.143
30-34 years	1.095	0.973	1.233
35-39 years	1.125	0.986	1.284
40+ years	1.454	1.208	1.750

Turner LY, *et al. BMJ Open* 2024; 14:e077710. doi: 10.1136/bmjopen-2023-077710

SUPPLEMENTARY INFORMATION

S8.2 Mode of birth

For outcome of readmission within 7 days
(other birth categories are compared with spontaneous vaginal birth)

	Readmitted within 7 days		
	OR	95% CI	
Assisted birth	1.838	1.622	2.083
Emergency Caesarean birth	1.888	1.685	2.116
Spontaneous vaginal birth	1.000	reference category	
Planned Caesarean birth	1.500	1.310	1.717

For outcome of readmission within 30 days
(other birth categories are compared with spontaneous vaginal birth)

	Readmitted within 30 days		
	OR	95% CI	
Assisted birth	1.828	1.642	2.036
Emergency Caesarean birth	2.041	1.854	2.247
Spontaneous vaginal birth		reference category	
Planned Caesarean birth	1.706	1.526	1.908

Turner LY, *et al. BMJ Open* 2024; 14:e077710. doi: 10.1136/bmjopen-2023-077710

SUPPLEMENTARY INFORMATION

S8.3 SHMI risk

For outcome of readmission within 7 days

	OR	95% CI	
SHMI risk	1.554	0.601	4.021

For outcome of readmission within 30 days

	OR	95% CI	
SHMI risk	1.546	0.671	3.560

S8.4 Understaffing by Registered Midwives, Maternity Assistants and Overall staffing

For outcome of readmission within 7 days

	Readmitted within 7 days		
	OR	95% CI	
Registered Midwife staffing <mean	1.065	0.974	1.166
Maternity Assistant staffing <mean	0.984	0.898	1.077
Overall staffing <mean	1.054	0.964	1.154

For outcome of readmission within 30 days

	Readmitted within 30 days		
	OR	95% CI	
Registered Midwife staffing <mean	1.041	0.965	1.123
Maternity Assistant staffing <mean	0.980	0.908	1.059
Overall staffing <mean	1.041	0.964	1.123

S8.5 Turnover (admissions + discharges) more than expected (mean) value for Trust

For outcome of readmission within 7 days

	Readmitted within 7 days		
	OR	95% CI	
Turnover > mean	0.972	0.889	1.062

For outcome of readmission within 30 days

	Readmitted within 30 days		
	OR	95% CI	
Turnover > mean	0.966	0.896	1.042

Turner LY, *et al. BMJ Open* 2024; 14:e077710. doi: 10.1136/bmjopen-2023-077710

SUPPLEMENTARY INFORMATION

S8.6 Skill mix lower than expected (mean) levels for service

For outcome of readmission within 7 days

	Readmitted within 7 days	
	OR	95% CI
Skill mix <mean	1.017	0.928 1.113

For outcome of readmission within 30 days

	Readmitted within 30 days	
	OR	95% CI
Skill mix <mean	0.979	0.907 1.057

Turner LY, *et al. BMJ Open* 2024; 14:e077710. doi: 10.1136/bmjopen-2023-077710

SUPPLEMENTARY INFORMATION

S9 Full models for staffing below the mean compared to mean staffing (HPPD)

For outcome of readmission within 7 days

Variable	OR readmission 7 days	95% CI
<20 years	1.066	0.810 1.402
20-24 years	1.000	reference category
25-29 years	0.914	0.790 1.057
30-34 years	0.983	0.853 1.133
35-39 years	0.983	0.837 1.153
40+ years	1.368	1.099 1.703
Assisted birth	1.873	1.648 2.128
Emergency Caesarean birth	1.912	1.701 2.148
Spontaneous vaginal birth	1.000	reference category
Planned Caesarean birth	1.518	1.321 1.745
Exposed to staffing below mean Registered midwives (HPPD)	1.108	1.003 1.223
Exposed to staffing below mean Maternity assistants (HPPD)	0.957	0.866 1.057
SHMI risk	0.687	0.108 4.360

For outcome of readmission within 30 days

Variable	OR readmission 30 days	95% CI
<20 years	1.065	0.840 1.350
20-24 years	1.000	reference category
25-29 years	0.969	0.856 1.098
30-34 years	1.024	0.906 1.156
35-39 years	1.046	0.913 1.198
40+ years	1.318	1.089 1.594
Assisted birth	1.850	1.657 2.065
Emergency Caesarean birth	2.077	1.883 2.291
Spontaneous vaginal birth	1.000	reference category
Planned Caesarean birth	1.707	1.522 1.915
Exposed to staffing below mean Registered midwives (HPPD)	1.080	0.994 1.174
Exposed to staffing below mean Maternity assistants (HPPD)	0.965	0.887 1.049
SHMI risk	0.8868	0.2421 3.2480

Turner LY, *et al. BMJ Open* 2024; 14:e077710. doi: 10.1136/bmjopen-2023-077710

SUPPLEMENTARY INFORMATION

S10. Full models with staffing expressed as individuals per midwife/maternity assistant (absolute level)

For outcome of readmission within 7 days

Variable	OR readmission 7 days	95% CI	
<20 years	1.066	0.811	1.402
20-24 years	1.000	reference category	
25-29 years	0.913	0.789	1.056
30-34 years	0.983	0.853	1.133
35-39 years	0.983	0.838	1.154
40+ years	1.368	1.099	1.702
Assisted birth	1.873	1.648	2.128
Emergency Caesarean birth	1.912	1.721	2.148
Spontaneous vaginal birth	1.000	reference category	
Planned Caesarean birth	1.519	1.322	1.745
Number of individuals to care for per midwife	1.063	0.960	1.177
Number of individuals to care for per maternity assistant	0.998	0.978	1.018
SHMI risk	0.691	0.109	4.370

For outcome of readmission within 30 days

Variable	OR readmission 30 days	95% CI	
<20 years	1.066	0.840	1.351
20-24 years	1.000	reference category	
25-29 years	0.969	0.856	1.098
30-34 years	1.025	0.907	1.157
35-39 years	1.046	0.913	1.199
40+ years	1.317	1.088	1.593
Assisted birth	1.850	1.657	2.064
Emergency Caesarean birth	2.077	1.883	2.291
Spontaneous vaginal birth	1.000	reference category	
Planned Caesarean birth	1.706	1.520	1.913
Number of individuals to care for per midwife	1.013	0.929	1.105
Number of individuals to care for per maternity assistant	1.002	0.986	1.019
SHMI risk	0.889	0.243	3.255

Turner LY, et al. BMJ Open 2024; 14:e077710. doi: 10.1136/bmjopen-2023-077710

SUPPLEMENTARY INFORMATION

S11 Additional analysis : High and low staffing categories compared with mean staffing

Observed (O) and Expected (E) staffing levels were calculated,
with E being the mean for each service.
The ratio of O/E was derived from these figures
The reference category was defined as O/E between 0.95 and 1.05
Low staffing was defined as O/E <0.95
High staffing was defined as O/E >1.05

This grouping allowed the mean (plus 5% either side of the mean) to be considered as an 'expected' level of staffing, allowing for a small amount of variability. The criteria for 'low' and 'high' were set outside this expected level.

This grouping was considered to be more meaningful that splitting the data into tertiles or quantiles or using standard deviations, as it is more reflective of a real clinical scenario and will provide a better insight to service planners.

For outcome of readmission within 7 days

Variable	OR readmission 7 days	95% CI	
<20 years	1.068	0.812	1.404
20-24 years	1.000	reference category	
25-29 years	0.914	0.790	1.057
30-34 years	0.984	0.854	1.134
35-39 years	0.984	0.838	1.154
40+ years	1.370	1.101	1.706
Assisted birth	1.873	1.648	2.127
Emergency Caesarean birth	1.911	1.701	2.147
Spontaneous vaginal birth	1.000	reference category	
Planned Caesarean birth	1.518	1.321	1.744
Midwifery staffing			
Low, 95% of mean or less	1.056	0.943	1.182
Near to mean 95%-105%	1.000	reference category	
High 105% of mean or more	0.973	0.862	1.098
Maternity assistant staffing			
Low, 95% of mean or less	0.924	0.816	1.046
Near to mean 95%-105%	1.000	reference category	
High 105% of mean or more	0.935	0.825	1.059
SHMI risk	0.693	0.111	4.335

SHMI Standardised Hospital Mortality Indicator

SUPPLEMENTARY INFORMATION

For outcome of readmission within 30 days

Variable	OR readmission 7 days	95% CI	
<20 years	1.066	0.841	1.351
20-24 years	1.000	reference category	
25-29 years	0.970	0.856	1.099
30-34 years	1.025	0.908	1.158
35-39 years	1.046	0.913	1.199
40+ years	1.319	1.090	1.595
Assisted birth	1.850	1.657	2.065
Emergency Caesarean birth	2.076	1.882	2.290
Spontaneous vaginal birth	1.000	reference category	
Planned Caesarean birth	1.705	1.520	1.913
Midwifery staffing			
Low, 95% of mean or less	1.002	0.911	1.102
Near to mean 95%-105%	1.000	reference category	
High 105% of mean or more	0.957	0.865	1.060
Maternity assistant staffing			
Low, 95% of mean or less	0.968	0.871	1.076
Near to mean 95%-105%	1.000	reference category	
High 105% of mean or more	0.968	0.870	1.077
SHMI risk	0.889	0.243	3.247

SHMI Standardised Hospital Mortality Indicator

S12 Frequency of exposure to staffing categories in study population

For registered midwives the staffing categories breakdown is as follows

1,258/64,250 (1.96%) staffing values known to be missing as reported in supplement 1.3

Staffing caregory	Frequency	percent
Low, 95% of mean or less	24,954	39.61
Near to mean 95%-105%	18,576	29.49
High 105% of mean or more	19,462	30.90

.

For maternity assistants the staffing categories breakdown is as follows

2,452/64,250 (3.82%) staffing values known to be missing as reported in supplement 1.3

Staffing caregory	Frequency	percent
Low, 95% of mean or less	26,374	42.68
Near to mean 95%-105%	12,520	20.26
High 105% of mean or more	22,903	37.06

SUPPLEMENTARY INFORMATION

S13 Results of full models by subset of population

Odds of readmission within 7 days, by subset of mode of birth

	Readmission rate	Exposed to staffing below mean Registered midwives (HPPD)	OR 95% CI	Exposed to staffing below mean Registered midwives (HPPD)	OR 95% CI
Whole cohort	3.18%	1.108	1.003, 1.223	0.957	0.866, 1.057
Assisted birth	4.59%	1.315	1.033, 1.675	0.876	0.689, 1.114
Emergency Caesarean	4.67%	1.219	0.991, 1.499	0.893	0.726, 1.098
Spontaneous birth	2.66%	0.983	0.849, 1.139	0.988	0.852, 1.146
Planned Caesarean	3.85%	1.113	0.858, 1.444	1.082	0.833, 1.405

Turner LY, *et al. BMJ Open* 2024; 14:e077710. doi: 10.1136/bmjopen-2023-077710

SUPPLEMENTARY INFORMATION

S14 Flow chart for data selection

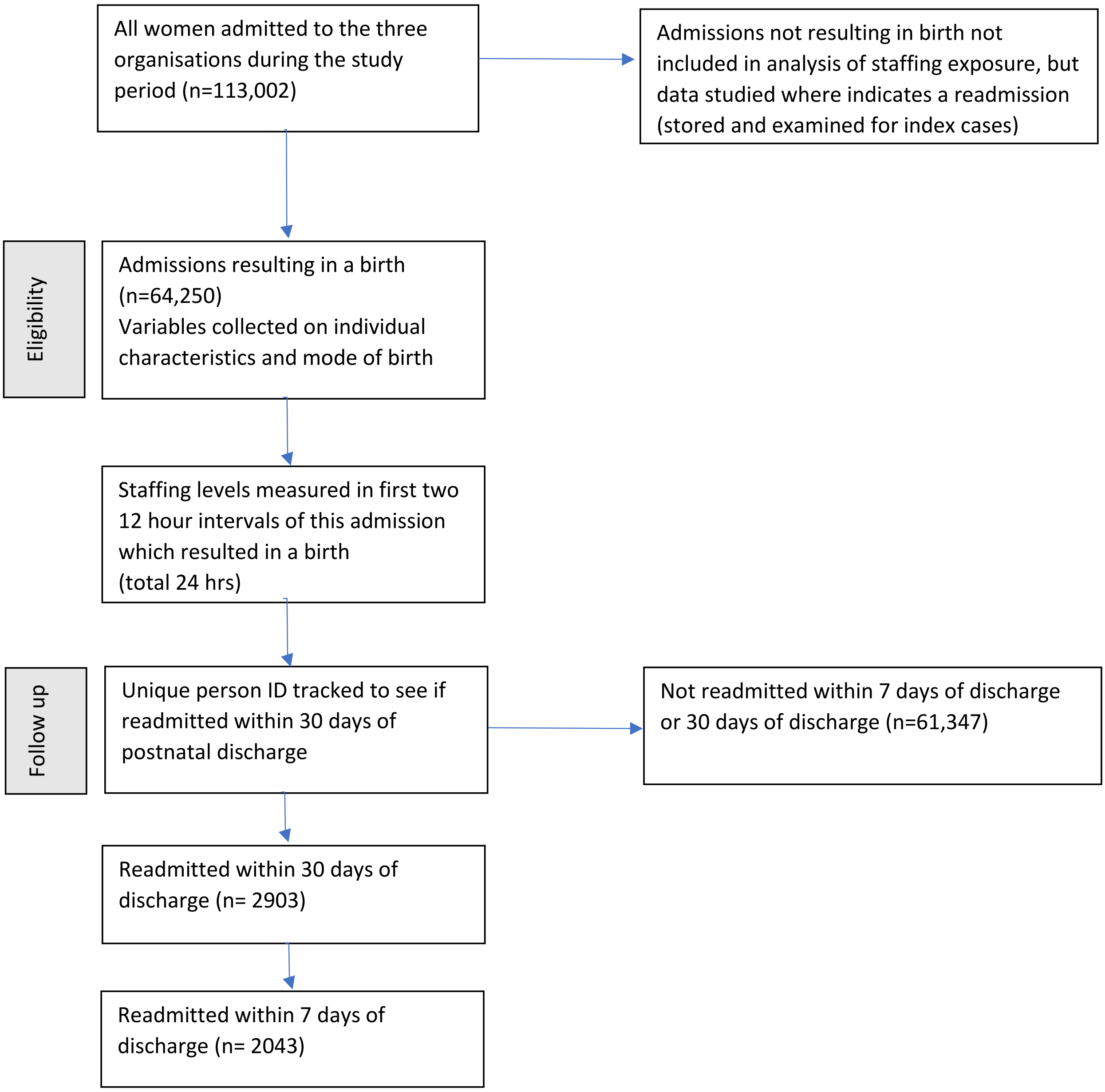

Supplement: Supplementary data [file bmjopen-2023-077710supp002.pdf]
