# Supplementary material for: Inpatient midwifery staffing levels and postpartum readmissions: a retrospective multicentre longitudinal study
Source: BMJ Open. 2024 Apr 3;14(4):e077710. doi: 10.1136/bmjopen-2023-077710 (PMC11146407; doi:10.1136/bmjopen-2023-077710)
Supplement: Supplementary data [file bmjopen-2023-077710supp001.pdf]

STROBE Statement—Checklist of items that should be included in reports of ***cohort studies***

	Item No	Recommendation	TRACKED CHANGES DOCUMENT Line No
**Title and abstract**	1	(*a*) Indicate the study's design with a commonly used term in the title or the abstract	1,28
		(*b*) Provide in the abstract an informative and balanced summary of what was done and what was found	30-58
**Introduction**			
Background/rationale	2	Explain the scientific background and rationale for the investigation being reported	82-117
Objectives	3	State specific objectives, including any prespecified hypotheses	118-120
**Methods**			
Study design	4	Present key elements of study design early in the paper	124-125
Setting	5	Describe the setting, locations, and relevant dates, including periods of recruitment, exposure, follow-up, and data collection	125-128
Participants	6	(*a*) Give the eligibility criteria, and the sources and methods of selection of participants. Describe methods of follow-up<br>(*b*) For matched studies, give matching criteria and number of exposed and unexposed	125-136
Variables	7	Clearly define all outcomes, exposures, predictors, potential confounders, and effect modifiers. Give diagnostic criteria, if applicable	136-186
Data sources/ measurement	8*	For each variable of interest, give sources of data and details of methods of assessment (measurement). Describe comparability of assessment methods if there is more than one group	136-186
Bias	9	Describe any efforts to address potential sources of bias	163-167, 190-192
Study size	10	Explain how the study size was arrived at	125-128, supp 13
Quantitative variables	11	Explain how quantitative variables were handled in the analyses. If applicable, describe which groupings were chosen and why	187-203, supp 11
Statistical methods	12	(*a*) Describe all statistical methods, including those used to control for confounding<br>(*b*) Describe any methods used to examine subgroups and interactions<br>(*c*) Explain how missing data were addressed<br>(*d*) If applicable, explain how loss to follow-up was addressed<br>(*e*) Describe any sensitivity analyses	187-206 supp 1, supp 10,11
**Results**			
Participants	13*	(a) Report numbers of individuals at each stage of study—eg numbers potentially eligible, examined for eligibility, confirmed eligible, included in the study, completing follow-up, and analysed<br>(b) Give reasons for non-participation at each stage<br>(c) Consider use of a flow diagram	214-216, supp 13
Descriptive data	14*	(a) Give characteristics of study participants (eg demographic, clinical, social) and information on exposures and potential confounders	Table 1, 215-222
		(b) Indicate number of participants with missing data for each variable of interest	Supplement1

For peer review only - http://bmjopen.bmj.com/site/about/guidelines.xhtml

Supplemental material BMJ Publishing Group Limited (BMJ) disclaims all liability and responsibility arising from any reliance placed on this supplemental material which has been supplied by the author(s) BMJ Open

		(c) Summarise follow-up time (eg, average and total amount)	188-190
Outcome data	15*	Report numbers of outcome events or summary measures over time	223-228

For peer review only

For peer review only - http://bmjopen.bmj.com/site/about/guidelines.xhtml

Turner LY, *et al. BMJ Open* 2024; 14:e077710. doi: 10.1136/bmjopen-2023-077710

Main results	16	(*a*) Give unadjusted estimates and, if applicable, confounder-adjusted estimates and their precision (eg, 95% confidence interval). Make clear which confounders were adjusted for and why they were included	Table 3, 246-258
		(*b*) Report category boundaries when continuous variables were categorized	Tables2-4
		(*c*) If relevant, consider translating estimates of relative risk into absolute risk for a meaningful time period	277-280
Other analyses	17	Report other analyses done—eg analyses of subgroups and interactions, and sensitivity analyses	277-288, S11
**Discussion**			
Key results	18	Summarise key results with reference to study objectives	295-301
Limitations	19	Discuss limitations of the study, taking into account sources of potential bias or imprecision. Discuss both direction and magnitude of any potential bias	360-382
Interpretation	20	Give a cautious overall interpretation of results considering objectives, limitations, multiplicity of analyses, results from similar studies, and other relevant evidence	295-304
Generalisability	21	Discuss the generalisability (external validity) of the study results	305-307
**Other information**			
Funding	22	Give the source of funding and the role of the funders for the present study and, if applicable, for the original study on which the present article is based	425-427

*Give information separately for exposed and unexposed groups.

Note: An Explanation and Elaboration article discusses each checklist item and gives methodological background and published examples of transparent reporting. The STROBE checklist is best used in conjunction with this article (freely available on the Web sites of PLoS Medicine at http://www.plosmedicine.org/, Annals of Internal Medicine at http://www.annals.org/, and Epidemiology at http://www.epidem.com/). Information on the STROBE Initiative is available at http://www.strobe-statement.org.

For peer review only

For peer review only - http://bmjopen.bmj.com/site/about/guidelines.xhtml

Turner LY, *et al. BMJ Open* 2024; 14:e077710. doi: 10.1136/bmjopen-2023-077710
