# Supplementary material for: Inpatient midwifery staffing levels and postpartum readmissions: a retrospective multicentre longitudinal study
Source: BMJ Open. 2024 Apr 3;14(4):e077710. doi: 10.1136/bmjopen-2023-077710 (PMC11146407; doi:10.1136/bmjopen-2023-077710)
Supplement: Author's manuscript [file bmjopen-2023-077710.draft_revisions.pdf]

BMJ Open is committed to open peer review. As part of this commitment we make the peer review history of every article we publish publicly available.

When an article is published we post the peer reviewers' comments and the authors' responses online. We also post the versions of the paper that were used during peer review. These are the versions that the peer review comments apply to.

The versions of the paper that follow are the versions that were submitted during the peer review process. They are not the versions of record or the final published versions. They should not be cited or distributed as the published version of this manuscript.

BMJ Open is an open access journal and the full, final, typeset and author-corrected version of record of the manuscript is available on our site with no access controls, subscription charges or pay-per-view fees (http://bmjopen.bmj.com).

If you have any questions on BMJ Open's open peer review process please email info.bmjopen@bmj.com

BMJ Open

Inpatient midwifery staffing levels and postpartum readmissions – a retrospective multi-centre longitudinal study

Journal:	*BMJ Open*
Manuscript ID	bmjopen-2023-077710
Article Type:	Original research
Date Submitted by the Author:	12-Jul-2023
Complete List of Authors:	Turner, Lesley; University of Southampton, School of Health Sciences<br>Saville, Christina; University of Southampton, School of Health Sciences<br>Ball, Jane; University of Southampton, School of Health Sciences<br>Culliford, David; NIHR Applied Research Collaboration Wessex; University of Southampton, School of Health Sciences<br>Dall'Ora, Chiara; University of Southampton, School of Health Sciences<br>Jones, Jeremy; University of Southampton, School of Health Sciences<br>Kitson-Reynolds, Ellen; University of Southampton, School of Health Sciences<br>Meredith, Paul; University of Southampton, School of Health Sciences<br>Griffiths, Peter; University of Southampton, School of Health Sciences; National Institute for Health Research Applied Research Centre (Wessex)
Keywords:	OBSTETRICS, Nursing Care, Postpartum Period, Human resource management < HEALTH SERVICES ADMINISTRATION & MANAGEMENT

SCHOLARONE™
Manuscripts

I, the Submitting Author has the right to grant and does grant on behalf of all authors of the Work (as defined in the below author licence), an exclusive licence and/or a non-exclusive licence for contributions from authors who are: i) UK Crown employees; ii) where BMJ has agreed a CC-BY licence shall apply, and/or iii) in accordance with the terms applicable for US Federal Government officers or employees acting as part of their official duties; on a worldwide, perpetual, irrevocable, royalty-free basis to BMJ Publishing Group Ltd ("BMJ") its licensees and where the relevant Journal is co-owned by BMJ to the co-owners of the Journal, to publish the Work in this journal and any other BMJ products and to exploit all rights, as set out in our licence.

The Submitting Author accepts and understands that any supply made under these terms is made by BMJ to the Submitting Author unless you are acting as an employee on behalf of your employer or a postgraduate student of an affiliated institution which is paying any applicable article publishing charge ("APC") for Open Access articles. Where the Submitting Author wishes to make the Work available on an Open Access basis (and intends to pay the relevant APC), the terms of reuse of such Open Access shall be governed by a Creative Commons licence – details of these licences and which Creative Commons licence will apply to this Work are set out in our licence referred to above.

Other than as permitted in any relevant BMJ Author's Self Archiving Policies, I confirm this Work has not been accepted for publication elsewhere, is not being considered for publication elsewhere and does not duplicate material already published. I confirm all authors consent to publication of this Work and authorise the granting of this licence.

For peer review only

Inpatient midwifery staffing levels and postpartum readmissions – a retrospective multi-centre longitudinal study

Abstract

Background
Preventing readmission to hospital after giving birth is a key priority, as rates have been rising along with associated costs. There are many contributing factors to readmission, and some are thought to be preventable. Nurse and midwife understaffing has been linked to deficits in care quality. This study explores the relationship between staffing levels and readmission rates in maternity settings.

Methods
We conducted a retrospective longitudinal study using routinely collected individual patient data in three maternity services in England from 2015-2020. Data on admissions, discharges and case mix were extracted from hospital administration systems. Staffing and workload were calculated per shift and the first two 12-hour shifts were used as the exposure window. Postpartum readmissions and staffing exposures for all birthing admissions were entered into a hierarchical multivariable logistic regression model to estimate the odds of readmission when staffing was below the mean level for the maternity service.

Results

64,250 maternal admissions resulted in birth and 2903 mothers were readmitted within 30 days of discharge (4.5%). Absolute levels of staffing ranged between 2.3 and 4.1 individuals per midwife in the three services. Below average midwifery staffing was associated with higher rates of postpartum readmissions within 7 days of discharge (adjusted OR 1.108, 95% CI 1.003, 1.223). The effect was smaller and not statistically significant for readmissions within 30 days of discharge (aOR 1.080, 95% CI 0.994, 1.174). Below average maternity assistant staffing was associated with lower rates of postpartum readmissions (7 days, aOR 0.957, 95% CI 0.867, 1.057; 30 days aOR 0.965, 95% CI 0.887, 1.049, both not statistically significant).

Conclusion

We found evidence that lower than expected midwifery staffing levels is associated with more postpartum readmissions. The nature of the relationship requires further investigation including examining potential mediating factors and reasons for readmission in maternity populations.

For peer review only - http://bmjopen.bmj.com/site/about/guidelines.xhtml

Keywords : readmission, post-partum, staffing

List of abbreviations

aOR	Adjusted odds ratio
NHS	National Health Service
HRG	Healthcare Resource Group
SHMI	Standardised Hospital Mortality Risk
HPPD	Hours Per Patient Day
RM	Registered Midwife
MA	Maternity Assistant

Strengths and Limitations of this study

Longitudinal design with individual level linking of staffing exposure during the birth admission to subsequent postpartum readmissions.

Adjustment for case mix, staff groups and organisational level variables using hierarchical multivariable modelling.

Staffing was measured in the first two 12-hour intervals of the hospital stay and did not account for the exposure to staffing for the whole hospital stay.

Considers exposure to staffing in the whole maternity service rather than close to women at ward level.

For peer review only - http://bmjopen.bmj.com/site/about/guidelines.xhtml

Background

In many countries of the world the majority of mothers give birth in hospital.[1] Preventing readmission due to potentially avoidable complications is a key priority, not only because of the negative experience and burden on the women affected, but because readmissions are costly.[2] Readmission rates are used as an indicator of the quality of care[3], and in some countries providers are held financially accountable for patient outcomes during the first 30 days after discharge for many patient groups.[4] Rates of postpartum readmission vary by country, with reported rates of 1.0% in the USA[5], 1.7% in Sweden[6] and 3.3% in England and Wales.[7]

Reports from England and the USA suggest that readmission rates have increased in recent years[7, 8]. This coincides with an increase in caesarean birth rates.[9] In England 4.3% of women having a caesarean birth are readmitted (1 in 23) compared with 2.9% of those having a vaginal birth (1 in 34).[7] The recent trend towards shorter length of hospital stay and reduced community provision may also contribute to increased readmission rates. However, whilst some research has pointed to an association between reduced length of stay and increased readmission rates [10, 11] this finding is not consistent in the research literature.[12, 13]

Readmission is a sign that a potentially avoidable complication has happened, and that further treatment is necessary. Readmissions are costly and puts the person at risk of further iatrogenic problems such as infections or medication errors. Furthermore, it is disruptive to families, and distressing to mothers who will have an altered experience of early motherhood. [14-16] Additional time spent in hospital as a result of readmission lasts 4.6 days on average for those who have had an emergency caesarean birth.[16] The most common reasons for readmission include wound complications, post-partum haemorrhage, hypertension, mastitis and thromboembolism. [5, 8,9,17,18] Postpartum readmissions typically occur within the first 14-20 days after discharge. [14,19]

Pressures on staffing within maternity services have been reported globally[20] but the consequences of this are poorly understood. In many areas of health care there is strong evidence that lower nurse staffing levels are associated with reduced quality of care and poorer patient outcomes (e.g. as measured by mortality[21], adverse events[22] and patient satisfaction).[23] Studies in medical and surgical inpatient settings have found that higher registered nurse staffing levels are associated with reduced readmission rates. [24-26] A scoping review of midwifery and nurse staffing for inpatient maternity services[27] found just two studies which measured maternal readmission in relation to staffing. These studies found that readmission rates were lower when more midwifery staff were employed[28] and the proportion of registered staff increased.[29] More evidence is needed on the relationship between maternity staffing levels, skill mix and readmission rates to understand whether previous findings are replicated and to understand the size of the effect if an association exists.

The aim of this study is to assess the relationship between individual patient-level exposure to staffing and postpartum readmission rates. A longitudinal design was adopted to enable women's exposure to staffing to be aligned to their outcomes, adjusting for individual risk factors.

Methods

We conducted a retrospective longitudinal study using individual patient records in multiple centres. Maternity inpatient stays from 13th April 2015- 29th February 2020 were extracted from the hospital Patient Administration System for 3 Acute National Health Service (NHS) Trusts in England. NHS

For peer review only - http://bmjopen.bmj.com/site/about/guidelines.xhtml

Trusts comprise one or more hospitals operating as a group under a combined management. Two of the Trusts had inpatient services all on one site. The third Trust had two sites where maternity hospitals were located in different areas surrounding a city.

The data included all women admitted to maternity wards, including those admitted via Day Assessment unit, totalling 113,002 admissions. Patient data was extracted for the date and time of admissions and discharges per maternity patient episode during the study period. The patient pseudonyms and dates were used to identify which postpartum women had been readmitted to the same maternity service for any cause within 7 days and 30 days of discharge. The primary reason for readmission was extracted from the ICD-10 code for the readmission episode. Case mix details for the index case included the age band on admission, the Healthcare Resource Group (HRG) and the Standardised Hospital Mortality Risk (SHMI) which includes adjustment for comorbidities and diagnosis group.[30] HRGs are standard groupings of clinically similar treatments which use comparable levels of healthcare resource. Admissions without a birth were excluded from the final data set as they would not be eligible to have a postpartum readmission.

Shift-level staffing data was obtained from Trust electronic roster systems which recorded all worked shifts and the grades of staff. This data comprised the time worked excluding breaks for the Registered Midwife group and Maternity Assistant Staff group and was matched to 12 hour periods, either 07.00 to 19.00 or 1900 to 07.00 to determine the staff time available in each time period. Staffing was measured by Hours Per Patient Day (HPPD) and this variable was generated by dividing the total worked seconds for the Staff group by the total ward occupancy in admitted person seconds and multiplying by 24. All admitted individuals were included in the occupancy measure because they all contribute to workload, with neonates accounting for 40% of the admitted population. The HPPD was calculated for Registered Midwife (RM) and Maternity Assistant (MA) staff separately, and the staff groups were combined as a measure of Overall staffing per 12-hour period per day. Skill mix was calculated per shift using the RM worked seconds as a proportion of the total worked seconds, which encompassed RMs and MAs. Registered Nurses were included in the Registered Midwife totals as there were very few (<0.1%) and were of a similar grade to midwives. Data was cleaned to remove shifts where patient occupancy is zero seconds, and to remove shifts with RM HPPD outliers (defined as RM HPPD < 0.5 or RM HPPD > 48).

Staffing for the index (birth) admission prior to the postpartum readmission was recorded. For the analysis, staffing was noted in the first two 12-hour periods of care in the Index admission (one interval of day shift and one of night shift). This method avoids recording staffing exposure for increased length of stay for a minority of complex cases. Longer time periods will skew the analysis as there is more opportunity to be exposed to understaffing. We defined the expected staffing as the mean HPPD for the maternity service in each Trust, and this was calculated separately for both day and night shifts. Each of the two 12-hour intervals of observed staffing (HPPD) was divided by expected staffing for the same period (HPPD) and these ratios were averaged for the first two 12 hour intervals, with values below 1 indicating a reduction in staff compared with expected levels.

An analysis using absolute staffing levels was also performed in a hierarchical model, nested in hospital Trust. The HPPD was converted into the number individuals cared for per midwife and individuals per maternity assistant by dividing 24 hours by the HPPD for each group. This allows service planners to see the potential differences of altering staffing levels in absolute terms using variables familiar to them. In addition, an analysis was undertaken using categories of staffing above and below the mean value. The categories were Low staffing (95% of mean or less) and High staffing (105% of mean or more) with near to mean staffing (95%-105% of mean) being used as the base level for comparison. This enabled non-linear relationships to be explored.

For peer review only - http://bmjopen.bmj.com/site/about/guidelines.xhtml

A further measure of ward activity and workload was included in the analysis. The variable 'turnover' equalled the total number of admissions plus discharges per Trust per 12 hour interval. Transfers between wards were not included in this measure. Mean turnover was calculated per Trust and variables were created to represent higher than expected turnover compared to the Trust mean, by day and night shifts.

Multi-level logistic regression was used with the primary outcomes of postpartum readmission within 7 days and 30 days of discharge, nested in hospital Trust. Readmissions within 7 days was included as this may be more sensitive to variation in care quality during index admissions[31 32], and longer time intervals increase the chance that readmission may be due to outpatient management.[33] The null model was the starting point and variables were added firstly based on indicators in the literature to suggest a potential effect on readmission (age, mode of birth), then staffing variables of interest (exposure to RM and MA staffing below the mean in the main analysis) and then remaining variables of SHMI risk, skill mix and turnover were added in sequence and only retained in the full model if they improved model fit using AIC and BIC values (see supplement S1 for model fit using this forward selection process).

All analyses were conducted in Stata (Release 16. College Station, TX: StataCorp LLC). Ethics permission was gained via the University of Southampton Ethics Committee ERGO 52957 and IRAS 128056.

Results

There were 113,002 separate maternal admissions between mid-April 2015 and the end of February 2020. Of these, 64,250 maternal admissions led to a birth. The Trusts differed in size with 5,260, 45,518 and 13,472 admissions leading to birth in each of the Trusts respectively. In the cohort 58.1% of women had a spontaneous vaginal birth, 12.9% assisted instrumental birth, 12.3% planned caesarean birth and 16.7% emergency caesarean birth.

Two thousand nine hundred and three women who gave birth were readmitted postnatally within 30 days of discharge (4.5%). Of those readmitted within 30 days, 70% were readmitted within the first 7 days and 89% within 14 days. Rates of readmission differed by birth method: spontaneous vaginal birth (3.7%), planned caesarean birth (5.9%), assisted birth (6.3%) and emergency caesarean birth (6.8%). The postpartum readmission rates following birth varied by Trust and were 1.8%, 4.9% and 4.3%. The mean length of stay for postpartum readmissions was 45.4 hours (median 22.5 hrs, IQR 3 hrs to 52 hrs). Infection/sepsis was the most common primary reason for readmission (677/2903, 23.3%). Postpartum haemorrhage accounted for 316/2903 readmissions (10.9%). Some readmissions appeared to be healthy mothers accompanying their newborn, although this represented only 125/2903 (4.3%) of maternal readmissions (supplement S2).

Staffing varied by Trust for those in our study cohort, with an average of 2.3, 3.6 and 4.1 individuals per midwife in the three services. More staff were available on day shifts compared with night shifts. The proportion of RM hours per Trust varies from 70.7%% to 77.4% between the Trusts, and almost all the registered staff are classified as midwives where this data is available (supplements S3-S5). For two of the Trusts the mean overall staffing in HPPD has shown an increase during the 5-year study period, whereas in the third Trust there is a stable level of overall staffing but a slight decrease in RM staff and increase in MA staffing over this period (approx. 4% change in each group).

For peer review only - http://bmjopen.bmj.com/site/about/guidelines.xhtml

There was more ward activity in terms of admissions and discharges on day shifts compared with night shifts (supplements S6-S7).

Univariable analyses on covariates and postpartum readmission

Age and the method of birth were statistically significant predictors of postpartum readmission in univariable analyses, with older mothers and those having assisted or operative birth at higher risk (see Table 1 and supplement S8). Higher SHMI risk was associated with higher risk of readmission, but this was not statistically significant. Women exposed to below average RM staffing and overall staffing had increased odds for readmission, but this was not statistically significant. Having more admissions and discharges than expected and a more diluted skill mix than average was not associated with increased postpartum readmissions at 7 days and 30 days.

Table 1: Univariable analyses of Readmission at 7 days and at 30 days from discharge

	Readmitted within 7 days			Readmitted within 30 days		
	OR	95% CI		OR	95% CI	
Age category	1.053	1.014	1.094	1.063	1.030	1.098
Mode of birth	0.849	0.822	0.878	0.857	0.833	0.881
Registered Midwife staffing <mean	1.065	0.974	1.166	1.041	0.965	1.123
Maternity Assistant staffing <mean	0.984	0.898	1.077	0.980	0.908	1.059
Overall staffing <mean	1.054	0.964	1.154	1.041	0.964	1.123
SHMI risk	1.554	0.601	4.021	1.546	0.671	3.560
Turnover > mean	0.972	0.889	1.062	0.966	0.896	1.042
Skill mix <mean	1.017	0.928	1.113	0.979	0.907	1.057

Relationship between staffing and postpartum readmission in multivariable models

Model fit was improved from the null model using a forward selection procedure with the addition of age category, birth method, RM and MA staffing and SHMI risk in that order. It was not improved with the addition of turnover and skill mix variables and therefore these variables are not shown in the full model. The variance inflation factors were below 2 for all variables which suggests that they are not collinear. Missing data is minimal for the variables studied (supplement S1).

Lower than expected midwifery staffing was associated with an 11% higher odds of postpartum readmissions within 7 days of discharge (adjusted OR 1.108, 95% CI 1.003, 1.223). The effect was smaller and not statistically significant for readmissions within 30 days of discharge (adjusted OR 1.080, 95% CI 0.994, 1.175). Lower than expected midwifery assistant staffing was associated with a lower odds of readmission at 7 days (OR 0.957, 95% CI 0.866, 1.057) and at 30 days (OR 0.965, 95% CI 0.887, 1.049). These relationships were not statistically significant in the multivariable model (see Table 2 and supplement S9).

For peer review only

For peer review only - http://bmjopen.bmj.com/site/about/guidelines.xhtml

Table 2 : 7 day and 30 day readmissions in the full multivariable models (staffing relative to the mean per service, supplement S9).

Variable	OR readmission 7 days	95% CI		OR readmission 30 days	95% CI	
<20 years	1.066	0.810	1.402	1.065	0.840	1.350
20-24 years	1.000 (base)			1.000 (base)		
25-29 years	0.914	0.790	1.057	0.969	0.856	1.098
30-34 years	0.983	0.853	1.133	1.024	0.906	1.156
35-39 years	0.983	0.837	1.153	1.046	0.913	1.198
40+ years	1.368	1.099	1.703	1.318	1.089	1.594
Assisted birth	1.873	1.648	2.128	1.850	1.657	2.065
Emergency Caesarean birth	1.912	1.701	2.148	2.077	1.883	2.291
Spontaneous vaginal birth	1.000 (base)			1.000 (base)		
Planned Caesarean birth	1.518	1.321	1.745	1.707	1.522	1.915
Exposed to staffing below mean Registered midwives (HPPD)	1.108	1.003	1.223	1.080	0.994	1.174
Exposed to staffing below mean Maternity assistants (HPPD)	0.957	0.866	1.057	0.965	0.887	1.049
SHMI risk	0.687	0.108	4.360	0.887	0.242	3.248

In models using absolute (rather than relative) staffing levels, an increase of one individual (mother or baby) per midwife was associated with a 6% increase in the odds of readmission at 7 days, aOR1.063 (95% CI 0.960, 1.178). There was no association with maternity assistant staffing (aOR 0.998, 95% CI 0.978, 1.018). Neither relationship was statistically significant (supplement 10)

Staffing was examined in categories to capture higher than average staffing as well as lower than average levels for the sensitive outcome measure of readmissions within 7 days (Table 3, supplement 11). When grouped as three categories, higher midwifery staffing (105% of mean or more) was associated with reduced odds of readmission compared with the category containing the mean. The category of lower than mean midwifery staffing (95% of mean or lower) was associated with increased odds of readmission. An inverted U-shaped pattern was observed with the point estimates for maternity assistant staffing when grouped as three categories. None of the relationships in the categorical analyses were statistically significant.

For peer review only

Table 3 : Multivariable analysis with staffing in categories above and below mean levels for readmissions within 7 days of discharge

Variable	OR readmission 7 days	95% CI	
<20 years	1.068	0.812	1.404
20-24 years	1.000 (base)		
25-29 years	0.914	0.790	1.057
30-34 years	0.984	0.854	1.134
35-39 years	0.984	0.838	1.154
40+ years	1.370	1.101	1.706
Assisted birth	1.873	1.648	2.127
Emergency Caesarean birth	1.911	1.701	2.147
Spontaneous vaginal birth	1.000 (base)		
Planned Caesarean birth	1.518	1.321	1.744
Midwifery staffing			
Low, 95% of mean or less	1.056	0.943	1.182
Near to mean 95%-105%	1.000 (base)		
High 105% of mean or more	0.973	0.862	1.098
Maternity assistant staffing			
Low, 95% of mean or less	0.924	0.816	1.046
Near to mean 95%-105%	1.000 (base)		
High 105% of mean or more	0.935	0.825	1.059
SHMI risk	0.693	0.111	4.335

Discussion

In this longitudinal study we analysed individual patient stays and concurrent staff rosters, and we found an association between midwifery staffing below the mean level and higher rates of postpartum readmission. This effect was stronger and statistically significant for readmissions within 7 days of discharge, with an increase in odds of 11% for understaffing compared with the organisation mean staffing levels. We estimate that if a midwife's workload is increased by one additional person, the odds of readmission within 7 days increases by 6.3%, although confidence intervals are wide and cross the null effect in this analysis of absolute staffing data. The direction of findings is in line with that of Gerova[28] who found that increased number of full-time-equivalent midwives in a service was associated with reduced postpartum readmissions. These findings are also seen in the nursing literature, in medical, surgical and paediatric settings. [24, 34]

The mean 30-day readmission rate in this study was 4.5%. The readmission rate following birth is much lower than with medical and surgical patients.[35] This is likely to be because obstetric patients are healthier and younger than other patient populations and because the index case is often undergoing a normal physiological process rather than management of a disease or condition.[4] The readmission median length of stay was 22.5 hours, which represents a sizeable provision within services each year when multiplied by the rate of readmission.

We found wide variation in the number of individuals cared for by each midwife, from 2.3 per midwife in one organisation to 4.1 per midwife in another. It is important to remember that this figure spans the inpatient maternity service where some women will receive one-to-one care in labour[36] and others will be on wards including neonates with fewer staff per person than in the birthing environment. One of the Trusts had a smaller service than the others, while also having higher rates of staffing and lower readmission rates. Smaller services require more staff to ensure services are available and to manage unscheduled demand.[37, 38] Smaller services may also be treating fewer complex cases which could account for lower readmission rates.[39] These variations were controlled for in our hierarchical analysis and also in the case-mix adjustment, so we can be

For peer review only - http://bmjopen.bmj.com/site/about/guidelines.xhtml

confident our analysis has taken account of these factors.

Risk factors associated with maternal postpartum readmission include age, ethnicity, parity, body mass index and mode of birth.[17 9] Individual characteristics were noted to account for most of the inter-hospital variance in postpartum readmission rates in the study by Clapp[40] and we saw further evidence of this as age and mode of birth were statistically significant predictors of readmission in all of our analyses. Staffing is a modifiable factor that has also been associated with rates of readmission[28 29]. Having more people to care for can affect the quality of care, as some aspects can be missed or incomplete as staff prioritise and ration their time.[41] Some activities such as medicines administration are commonly prioritised, whereas patient teaching and discharge preparation may be seen as less urgent, and therefore more likely to be incomplete or missed.[34] This has been found in a number of studies including those in Malaysia[42], Australia[43], USA[34] and South Korea[44], although not all of these studies are in maternity populations.

The mechanisms for an association between staffing and readmissions need further study. Weiss et al[45] found a sequential pathway from high registered nurse staffing, to improved discharge teaching quality and readiness, to decreased odds of readmission for medical-surgical patients. However, this type of study has not been replicated in the maternity population as far as we are aware. Proposed mechanisms include reduced staff time for recognising deterioration[46], time for education[34], coordination of care[25], hand hygiene compliance[3, 47], discharge planning[41] and medicines optimisation.[14] We found that infection or sepsis was listed as the primary diagnosis in 23% of the postpartum readmissions. Although this was the most common identifiable reason for readmission it was not as high as that reported in the literature, where infection has been implicated in 62% of all postpartum admissions[9] and 82% of readmissions following caesarean birth[18]. Nevertheless, this is still a sizeable figure so mediating pathways from staffing linked to infection and then readmission may be worthy of exploring.

We found an association between midwifery staffing below the mean level and higher rates of postpartum readmissions within 7 days of discharge. We went on to look at staffing levels in categories just below and just above the mean levels for this outcome. This categorical analysis of midwifery staffing found fewer readmissions when services were staffed above mean levels and more readmissions when staffed below mean levels, although these were not statistically significant. Service planners should consider setting staffing above mean levels if these findings are confirmed, as it is possible that mean levels in themselves represent suboptimal understaffing.

There was no clear relationship between maternity assistant staffing levels and readmission rates in our study. It may be that maternity assistants perform fewer sensitive activities which could be related to the risk of readmission, although there is very little published evidence on assistants in maternity services to compare our findings to.

Strengths and Limitations

There is limited evidence to determine optimal staffing levels in maternity services. Our research adds to the body of evidence by highlighting the association between staffing levels of midwives and rates of postpartum readmissions. Our analysis has taken account of case mix and between-hospital differences when estimating effects. Another strength of our approach is the longitudinal and granular design, so we have been able to measure the individual's exposure to staffing per shift, rather than estimating via monthly or annual data. Our approach has an advantage over sequential panel studies as the longitudinal design allows direct individual-level linking of staffing exposure to readmission despite the time lag between these variables.

For peer review only - http://bmjopen.bmj.com/site/about/guidelines.xhtml

The amount of missing data is minimal, and therefore we believe this does not materially affect the conclusions drawn. A limitation of our study is that we have been unable to extract information on the staffing close to each woman at the ward level, as two of the Trusts only had staffing data available as a whole service. Also we have been unable to account for post-discharge care in the community, which is likely to have a bearing on readmission rates if women have differing opportunities to seek early intervention elsewhere.[17]

Areas for future research

Any measurable reduction in hospital readmission rates would offer significant financial benefits to hospitals.[4] The cost of increasing staffing could be offset by the potential savings in the future. Cost-effectiveness studies are needed to investigate the consequences of different staffing configurations in relation to the quality of care and related costs and savings. In future research, the components of services that help to reduce readmissions could be explored so resources are applied in areas known to be effective. This may mean targeting increased attention towards at-risk populations that account for a high proportion of readmissions.[9,19] The provision of additional staff alone may not be fully effective in reducing readmissions unless they are focusing activity on areas of need or critical activities.

Conclusion

Low midwifery staffing in maternity units is associated with increased rates of readmissions within seven days after discharge but there was no adverse effect from lower assistant staffing. This is an important finding especially as readmission rates have increased in some countries in recent years, they are costly to services and distressing for families. Limiting the number of individuals cared for per midwife may have benefits beyond the inpatient stay and result in cost savings if readmissions can be reduced.

3966 words, includes tables, excluding abstract

For peer review only

For peer review only - http://bmjopen.bmj.com/site/about/guidelines.xhtml

References

1. World Health Organization. Global Health Observatory data repository 2023 [Available from: https://www.who.int/data/gho.
2. Vest JR, Gamm LD, Oxford BA, Gonzalez MI, Slawson KM. Determinants of preventable readmissions in the United States: a systematic review. Implementation science. 2010;5(1):1-28.
3. Blume KS, Dietermann K, Kirchner-Heklau U, Winter V, Fleischer S, Kreidl LM, et al. Staffing levels and nursing-sensitive patient outcomes: Umbrella review and qualitative study. Health Services Research. 2021;00:1-23.
4. Brown TL. Nurse Staffing, Satisfaction and Hospital Readmission Rates in Skilled Nursing Facilities: A Quantitative, Correlational Study. Doctoral Thesis.: University of Phoenix; 2020.
5. Clapp MA, Little SE, Zheng J, Kaimal AJ, Robinson JN. Hospital-level variation in postpartum readmissions. JAMA. 2017;317(20):2128-9.
6. Ellberg L, Lundman B, Persson M, Hogberg U. Comparison of health care utilization of postnatal programs in Sweden. Journal of Obstetric, Gynecologic, & Neonatal Nursing. 2005;34(1):55-62.
7. National Maternity and Perinatal Audit. Clinical Report 2022. Based on births in NHS maternity services in England and Wales between 1 April 2018 and 31 March 2019 2022 [Available from: https://maternityaudit.org.uk/FilesUploaded/Ref%20336%20NMPA%20Clinical%20Report_2022.pdf]
8. Clapp MA, Little SE, Zheng J, Robinson JN. A multi-state analysis of postpartum readmissions in the United States. American journal of obstetrics and gynecology. 2016;215(1):113. e1.
9. Sharvit M, Rubinstein T, Ravid D, Shechter-Maor G, Fishman A, Biron-Shental T. Patients with high-risk pregnancies and complicated deliveries have an increased risk of maternal postpartum readmissions. Archives of gynecology and obstetrics. 2014;290:629-33.
10. Liu S, Heaman M, Kramer MS, Demissie K, Wen SW, Marcoux S, et al. Length of hospital stay, obstetric conditions at childbirth, and maternal readmission: a population-based cohort study. American journal of obstetrics and gynecology. 2002;187(3):681-7.
11. Wen T, Yu VX, Wright JD, Goffman D, Attenello F, Mack WJ, et al. Postpartum length of stay and risk for readmission among women with preeclampsia. The Journal of Maternal-Fetal & Neonatal Medicine. 2020;33(7):1086-94.
12. Ford JB, Algert CS, Morris JM, Roberts CL. Decreasing length of maternal hospital stay is not associated with increased readmission rates. Australian and New Zealand Journal of Public Health. 2012;5(36):430-4.
13. Pervez S, Sharawi N, Blake L, Habib A, Brookfield K, Carvalho B. Impact of enhanced recovery after caesarean delivery on maternal outcomes: A systematic review and meta-analysis. Anaesthesia, Critical Care & Pain Medicine. 2021(100935):1-11.
14. Combs CA, Goffman D, Pettker CM, Medicine SfM-F, Committee Q. Society for Maternal-Fetal Medicine special statement: a critique of postpartum readmission rate as a quality metric. American Journal of Obstetrics and Gynecology. 2022;226(4):B2-B9.
15. Feenstra MM, Nilsson I, Danbjørg DB. Broken expectations of early motherhood: Mothers' experiences of early discharge after birth and readmission of their infants. Journal of clinical nursing. 2019;28(5-6):870-81.
16. Panda S, Begley C, Daly D. Readmission following caesarean section: Outcomes for women in an Irish maternity hospital. British Journal of Midwifery. 2016;24(5):322-8.
17. Szafrańska M, Begley C, Carroll M, Daly D. Factors associated with maternal readmission to hospital, attendance at emergency rooms or visits to general practitioners within three months postpartum. European Journal of Obstetrics & Gynecology and Reproductive Biology. 2020;254:251-8.
18. Kipkore SJ. Risk Factors, Indications and Outcomes of Post Cesarean Section Readmission at Kenyatta N. Hospital: a Case-control Study. Masters Thesis.: University of Nairobi; 2022.

For peer review only - http://bmjopen.bmj.com/site/about/guidelines.xhtml

19. Wen T, Overton EE, Sheen J-J, Attenello FJ, Mack WJ, D'Alton ME, et al. Risk for postpartum readmissions and associated complications based on maternal age. The Journal of Maternal-Fetal & Neonatal Medicine. 2019;34(9):1375-81.
20. UNFPA. The State of The World's Midwifery 2021 [Available from: https://www.unfpa.org/sites/default/files/resource-pdf/21-038-UNFPA-SoWMY2021-FastFacts-ENv4302.pdf.]
21. Needleman J, Liu, L., Shang, J., Larson, E. Association of registered nurse and nursing support staffing with inpatient hospital mortality. BMJ quality & safety. 2020;29:10-8.
22. Kane RL, Shamliyan TA, Mueller C, Duval S, Wilt TJ. The association of registered nurse staffing levels and patient outcomes: systematic review and meta-analysis. Medical care. 2007:1195-204.
23. Aiken LH, Sloane DM, Ball J, Bruyneel L, Rafferty AM, Griffiths P. Patient satisfaction with hospital care and nurses in England: an observational study. BMJ open. 2018;8(1):e019189.
24. Ma C, McHugh MD, Aiken LH. Organization of hospital nursing and 30-day readmissions in Medicare patients undergoing surgery. Medical Care. 2015;53(1):65-70.
25. McHugh MD, Berez J, Small DS. Hospitals with higher nurse staffing had lower odds of readmissions penalties than hospitals with lower staffing. Health Affairs. 2013;32(10):1740-7.
26. Lasater KB, McHugh M, Rosenbaum PR, Aiken LH, Smith H, Reiter JG, et al. Valuing hospital investments in nursing: multistate matched-cohort study of surgical patients. BMJ Quality & Safety. 2021;30(1):46-55.
27. Turner L, Griffiths P, Kitson-Reynolds E. Midwifery and nurse staffing of inpatient maternity services – a systematic scoping review of associations with outcomes and quality of care. Midwifery. 2021;103:103118.
28. Gerova V, Griffiths P, Jones S, Bick D. The association between midwifery staffing and outcomes in maternity units in England: observational study using routinely collected data. Kings College London; 2010. https://eprints.soton.ac.uk/168511/
29. Kim SJ, Han KT, Lee HJ, Kwon JA, Park EC. Positive effects of medical staffing on readmission within 30 days after discharge: a retrospective analysis of obstetrics and gynecology data. European journal of public health. 2016;26(6):935-9.
30. Digital N. Summary Hospital-level Mortality Indicator (SHMI) 2023 [Available from: https://digital.nhs.uk/data-and-information/publications/ci-hub/summary-hospital-level-mortality-indicator-shmi.
31. Chin DL, Bang H, Manickam RN, Romano PS. Rethinking thirty-day hospital readmissions: shorter intervals might be better indicators of quality of care. Health Affairs. 2016;35(10):1867-75.
32. Graham KL, Wilker EH, Howell MD, Davis RB, Marcantonio ER. Differences between early and late readmissions among patients: a cohort study. Annals of Internal Medicine. 2015;162(11):741-9.
33. Goldfield NI, McCullough EC, Hughes JS, Tang AM, Eastman B, Rawlins LK, et al. Identifying potentially preventable readmissions. Health care financing review. 2008;30(1):75.
34. Tubbs-Cooley HL, Cimiotti JP, Silber JH, Sloane DM, Aiken LH. An observational study of nurse staffing ratios and hospital readmission among children admitted for common conditions. BMJ quality & safety. 2013;22(9):735-42.
35. NHS Digital. Emergency readmissions to hospital within 30 days of discharge by diagnosis 2022 [Available from: https://digital.nhs.uk/data-and-information/publications/statistical/compendium-emergency-readmissions/current/emergency-readmissions-to-hospital-within-30-days-of-discharge.
36. Care Quality Commission, Picker Institute Europe. Maternity Survey, 2019 [data collection]. 2021. UK Data Service. SN: 8790
37. Vaughan L, Edwards N. The problems of smaller, rural and remote hospitals: separating facts from fiction. Future Healthcare Journal. 2020;7(1):38.
38. Edwards N, Vaughan, L., Palmer, B. Maternity services in smaller hospitals: a call to action 2020 [Available from: https://www.nuffieldtrust.org.uk/research/maternity-services-in-smaller-hospitals-a-call-to-action.]

For peer review only - http://bmjopen.bmj.com/site/about/guidelines.xhtml

39. Fischer C, Lingsma HF, Marang-van de Mheen PJ, Kringos DS, Klazinga NS, Steyerberg EW. Is the readmission rate a valid quality indicator? A review of the evidence. PloS one. 2014;9(11):e112282.
40. Clapp MA, Little SE, Zheng J, Robinson JN, Kaimal AJ. The relative effects of patient and hospital factors on postpartum readmissions. Journal of Perinatology. 2018;38(7):804-12.
41. Simpson KR, Lyndon A, Ruhl C. Consequences of inadequate staffing include missed care, potential failure to rescue, and job stress and dissatisfaction. Journal of Obstetric, Gynecologic & Neonatal Nursing. 2016;45(4):481-90.
42. Nahasaram ST, Ramoo V, Lee WL. Missed nursing care in the Malaysian context: A cross-sectional study from nurses' perspective. Journal of nursing management. 2021;00:1-9.
43. Albsoul RAM. Investigating missed nursing care in an Australian acute care hospital: An exploratory study. [(Doctoral dissertation).]: Queensland University of Technology; 2019.
44. Lee E, Kalisch BJ. Identification and comparison of missed nursing care in the United States and South Korea. Journal of Clinical Nursing. 2021;00:1-11.
45. Weiss ME, Yakusheva O, Bobay KL. Quality and cost analysis of nurse staffing, discharge preparation, and postdischarge utilization. Health services research. 2011;46(5):1473-94.
46. Jeffs L, Dhalla I, Cardoso R, Bell CM. The perspectives of patients, family members and healthcare professionals on readmissions: preventable or inevitable? Journal of interprofessional care. 2014;28(6):507-12.
47. Rae PJ, Pearce S, Greaves PJ, Dall'Ora C, Griffiths P, Endacott R. Outcomes sensitive to critical care nurse staffing levels: A systematic review. Intensive and Critical Care Nursing. 2021;67:103110.

For peer review only

Lesley Turner*, School of Health Sciences, University of Southampton, Southampton, England

*Corresponding author : Lesley Turner
Email lyt1g19@soton.ac.uk

Christina Saville, School of Health Sciences, University of Southampton, Southampton, England

Jane Ball, School of Health Sciences, University of Southampton, Southampton, England

David Culliford, NIHR Applied Research Collaboration Wessex, School of Health Sciences, University of Southampton, Southampton, England

Chiara Dall'ora, School of Health Sciences, University of Southampton, Southampton, England

Jeremy Jones, School of Health Sciences, University of Southampton, Southampton, England

Ellen Kitson-Reynolds, School of Health Sciences, University of Southampton, Southampton, England

Paul Meredith, School of Health Sciences, University of Southampton, Southampton, England

Peter Griffiths, School of Health Sciences, University of Southampton, National Institute for Health Research Applied Research Centre (Wessex), Southampton, England

Funding acquisition : PG, CDO, JB, DC, JJ, FL, PM, CS.
FL provided study co-ordination.
CS and PM cleaned and linked data from different sources
LT, PG, CDO, JB, DC, JJ, PM, and CS developed the methodology.
LT conducted the investigation and analysis and wrote the original draft.
LT, PG, CDO, JB, DC, PM, CS and EKR reviewed the results and contributed to interpretation.
PG, EKR and JB supervised this project.
DC provided statistical advice.
All authors reviewed the manuscript.

Acknowledgements.
The views expressed are those of the author(s) and not necessarily those of the National Institute for Health Research, the Department of Health and Social Care, 'arms-length' bodies or other government departments. Frankie Lambert (FL) made contributions to the work that fall short of ICMJE authorship criteria but we wish her contribution to be recognised.

For peer review only - http://bmjopen.bmj.com/site/about/guidelines.xhtml

SUPPLEMENTARY INFORMATION

Table of contents

S1 Model fit and missing data 2
1.1 Model fit by AIC and BIC 2
1.2 Collinearity testing 2
1.3 Missing data 2
S2 Diagnosis codes for the 2903 postpartum readmissions 3
S3 Absolute staffing levels expressed as Individuals per RM and MA 5
S4 Staffing on day and night shifts over time in each of the Trusts 6
S5 Skill mix of staff within the three Trusts 7
S6 Trends in staffing over time 8
S7 Mean admissions per day and night 9
S8 Univariable analyses 10
S8.1 Age category 10
S8.2 Mode of birth 11
S8.3 SHMI risk 12
S8.4 Understaffing by Registered Midwives, Maternity Assistants and Overall staffing compared with expected staffing 12
S8.5 Turnover (admissions + discharges) more than expected 13
S8.6 Skill mix lower than expected 13
S9 Full models for staffing below the mean compared to mean staffing (HPPD) 14
S10. Staffing expressed as absolute values : number of individuals per midwife / maternity care assistant 15
S11 Additional analysis : High and low staffing categories compared with mean staffing....16

For peer review only - http://bmjopen.bmj.com/site/about/guidelines.xhtml

SUPPLEMENTARY INFORMATION

S1 Model fit and missing data

1.1 Model fit by AIC and BIC

Postpartum readmission	7 days from discharge model		30 days from discharge model	
	AIC	BIC	AIC	BIC
Empty model nested in Trust	18036.62	18054.76	23539.27	23557.41
Age category added	18031.47	18058.68	23527.22	23554.43
Mode of birth added	17667.94	17703.96	23024.88	23060.91
RN<mean staffing added	17240.87	17285.81	22518.88	22563.81
NA<mean staffing added	17112.23	17166.03	22296.37	22350.17
SHMI added	17008.74	17071.47	22149.12	22211.84
Skill mix added (did not improve model fit so excluded from full model)	17010.53	17082.21	22148.77	22220.45
Turnover added (did not improve model fit so excluded from full model)	17012	17092.65	22149.99	22230.63

1.2 Collinearity testing

Test for collinearity

regress INDEXHRGPostnatalR7days understaffRNless1 understaffNAless1 age_category_combined birth_method SHMI_risk HighrerExpectedTurnover LessExpectedSkillMix

VIF

understaffRNless1	1.37
understaffNAless1	1.90
age_category_combined	1.00
birth_method	1.00
SHMI_risk	1.00
Higher expected turnover	1.02
Less expected skill mix	1.56

VIF scores are low so collinearity not detected

1.3 Missing data

Age None
Length of stay None
Mode birth None
SHMI 385/64,250 (0.6%)
Staffing RM 1,258/64,250 (1.96%)
Staffing MA 2,452/64,250 (3.82%)
(MA missing from Trust A after 28.1.2019)

For peer review only - http://bmjopen.bmj.com/site/about/guidelines.xhtml

SUPPLEMENTARY INFORMATION

S2 Diagnosis codes for the 2903 postpartum readmissions

**ICD codes for primary diagnosis matched to description** Where categories are broad the second diagnosis code has been reported at the end of this table	Count	percentage
Total number of readmissions	**2903**	
Other complications of the puerperium, not elsewhere classified*	482	16.6%
Care and examination of lactating mother**	373	12.8%
Delayed and secondary postpartum haemorrhage	310	10.7%
Puerperal sepsis	246	8.5%
Infection of obstetric surgical wound	227	7.8%
Other specified diseases and conditions complicating pregnancy, childbirth and the puerperium***	159	5.5%
Disruption of caesarean section wound	104	3.6%
Disruption of perineal obstetric wound	103	3.5%
Maternal care for rhesus isoimmunization	71	2.4%
Nonpurulent mastitis associated with childbirth	63	2.2%
Unspecified maternal hypertension	57	2.0%
Haematoma of obstetric wound	45	1.6%
Urinary tract infection following delivery	43	1.5%
Persons encountering health services in other specified circumstances	40	1.4%
Pyrexia of unknown origin following delivery	39	1.3%
Fitting and adjustment of urinary device	33	1.1%
Anaemia complicating pregnancy, childbirth and the puerperium	30	1.0%
Diseases of the digestive system complicating pregnancy, childbirth and the puerperium	26	0.9%
Care and examination immediately after delivery	25	0.9%
Spinal and epidural anaesthesia-induced headache during the puerperium	24	0.8%
Other prophylactic chemotherapy	23	0.8%
Spinal and epidural anaesthesia-induced headache during labour and delivery	21	0.7%
Gestational [pregnancy-induced] hypertension	20	0.7%
Other infection of genital tract following delivery	17	0.6%
Prophylactic immunotherapy	16	0.6%
Other specified pregnancy-related conditions	15	0.5%
Other maternal infectious and parasitic diseases complicating pregnancy, childbirth and the puerperium	14	0.5%
Pre-eclampsia, unspecified	14	0.5%
Retained portions of placenta and membranes, without haemorrhage	13	0.4%
Routine postpartum follow-up	11	0.4%
Supervision of other normal pregnancy	11	0.4%
Other specified puerperal infections	9	0.3%
Diseases of the respiratory system complicating pregnancy, childbirth and the puerperium	8	0.3%
Unspecified infection of urinary tract in pregnancy	8	0.3%
Complication of the puerperium, unspecified	7	0.2%
Diseases of the circulatory system complicating pregnancy, childbirth and the puerperium	7	0.2%
Obstetric blood-clot embolism	7	0.2%
Attention to surgical dressings and sutures	6	0.2%
Elevated blood-pressure reading, without diagnosis of hypertension	6	0.2%
Examination and observation for other specified reasons	6	0.2%

For peer review only - http://bmjopen.bmj.com/site/about/guidelines.xhtml

SUPPLEMENTARY INFORMATION

**ICD codes for primary diagnosis matched to description** Where categories are broad the second diagnosis code has been reported at the end of this table	**Count**	**percentage**
Mental disorders and diseases of the nervous system complicating pregnancy, childbirth and the puerperium	6	0.2%
Other immediate postpartum haemorrhage	6	0.2%
Maternal care due to uterine scar from previous surgery	5	0.2%
Other and unspecified disorders of breast associated with childbirth	5	0.2%
Postpartum coagulation defects	5	0.2%
Second degree perineal laceration during delivery	5	0.2%
Superficial thrombophlebitis in the puerperium	5	0.2%
Unspecified pre-existing hypertension complicating pregnancy, childbirth and the puerperium	5	0.2%
Other conditions with ICD codes affecting <5 women in dataset each (condensed for reporting in this table)	115	4.0%
Blank	7	0.2%

* 68/478 had blank second diagnosis code, 179/478 had ICD indicating a type of pain as second diagnosis code
** 226/373 had blank second diagnosis code, 125/373 had 'Healthy person accompanying sick person' as second diagnosis code
***24/159 had blank second diagnosis code, no pattern seen in remaining ICD codes

When totalling the above 677/2903 (23.3%) of postpartum readmissions were due to infection or sepsis, 316/2903 (10.9%) were due to haemorrhage

Missing ICD diagnosis code for readmissions 7/2903 (0.2%)

For peer review only - http://bmjopen.bmj.com/site/about/guidelines.xhtml

SUPPLEMENTARY INFORMATION

S3 Absolute staffing levels expressed as Individuals per RM and MA

Staffing calculated for women having an admission which leads to birth over first two 12hour intervals (study cohort)

Trust	Mean RM HPPD	Individuals per Midwife	Mean MA HPPD	Individuals per Maternity Assistant
A	11.747	2.330	4.869	5.934
B	5.940	4.104	1.759	14.205
C	6.906	3.576	2.650	9.387
All Trusts (non weighted average)	6.520	3.879	2.146	12.67

Individuals cared for per staff member = 24/HPPD.

For peer review only

For peer review only - http://bmjopen.bmj.com/site/about/guidelines.xhtml

SUPPLEMENTARY INFORMATION

S4 Staffing on day and night shifts over time in each of the Trusts

Data for staffing for whole admitted population

Year	DAY or NIGHT	Mean RM HPPD	Mean MA HPPD	Mean Overall HPPD
Trust A				
2015	Day	10.75	4.56	15.32
2015	Night	11.10	3.66	14.77
2016	Day	11.73	5.47	17.20
2016	Night	11.76	5.18	16.94
2017	Day	14.22	6.04	20.26
2017	Night	13.54	5.46	19.00
2018	Day	14.08	5.97	20.04
2018	Night	13.81	5.09	18.90
2019	Day	14.68	5.77	19.06
2019	Night	14.28	4.84	17.96
2020	Day	12.57	Not available	Not available
2020	Night	12.44	Not available	Not available
Trust B				
2015	Day	7.00	1.82	8.83
2015	Night	5.09	1.52	6.60
2016	Day	6.93	1.86	8.80
2016	Night	4.99	1.53	6.52
2017	Day	7.19	2.08	9.27
2017	Night	5.04	1.65	6.70
2018	Day	6.71	1.81	8.52
2018	Night	4.81	1.34	6.15
2019	Day	6.92	2.15	9.07
2019	Night	4.89	1.72	6.61
2020	Day	6.61	2.35	8.96
2020	Night	4.84	2.09	6.93
Trust C				
2015	Day	7.05	1.98	9.03
2015	Night	5.82	2.01	7.83
2016	Day	7.02	2.17	9.19
2016	Night	5.91	2.12	8.03
2017	Day	7.23	2.52	9.75
2017	Night	6.06	2.37	8.44
2018	Day	7.34	2.76	10.10
2018	Night	6.16	2.64	8.80
2019	Day	7.91	2.84	10.76
2019	Night	6.96	2.85	9.81
2020	Day	8.62	3.25	11.87
2020	Night	7.84	3.10	10.94

RM = registered midwife MA=maternity assistant

For peer review only - http://bmjopen.bmj.com/site/about/guidelines.xhtml

SUPPLEMENTARY INFORMATION

S5 Skill mix of staff within the three Trusts

	Sum of worked hours and % of total in each staff group					
	Total sum of worked hrs	RM Band 6 or above	Registered midwives	Registered nurses	Band 3 care assistants	Band 2 care assistants
Trust A*	316,880	33.5%	70.7%	0.09%	2.2%	26.8%
Trust B	1,990,058	60.4%	77.4%	0%	3.5%	19.1%
Trust C	933,846	59.8%	73.4% (nurses not listed separately)		7.3%	19.2%

*up to 28.1.2019 as care assistant unavailable after this date

For peer review only - http://bmjopen.bmj.com/site/about/guidelines.xhtml

SUPPLEMENTARY INFORMATION

S6 Trends in staffing over time

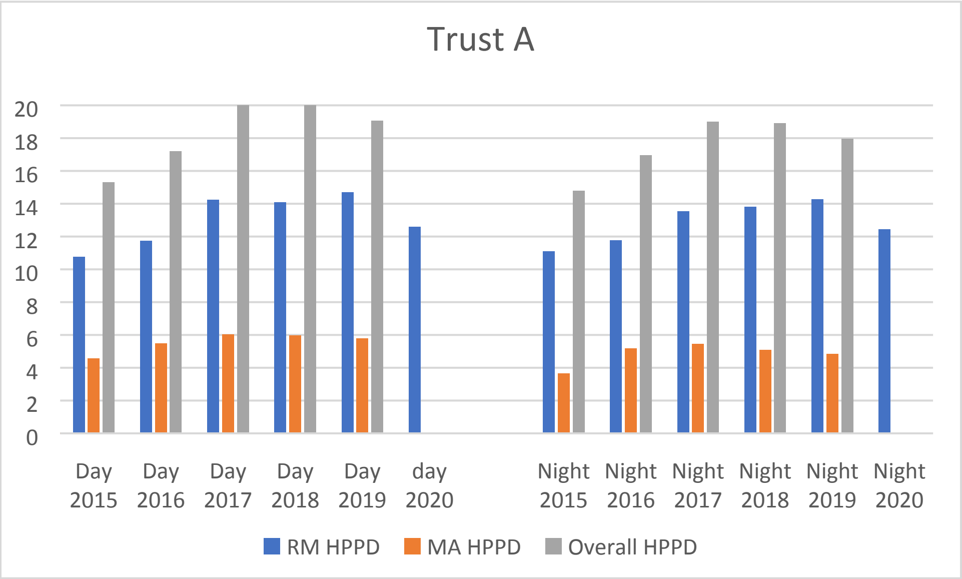

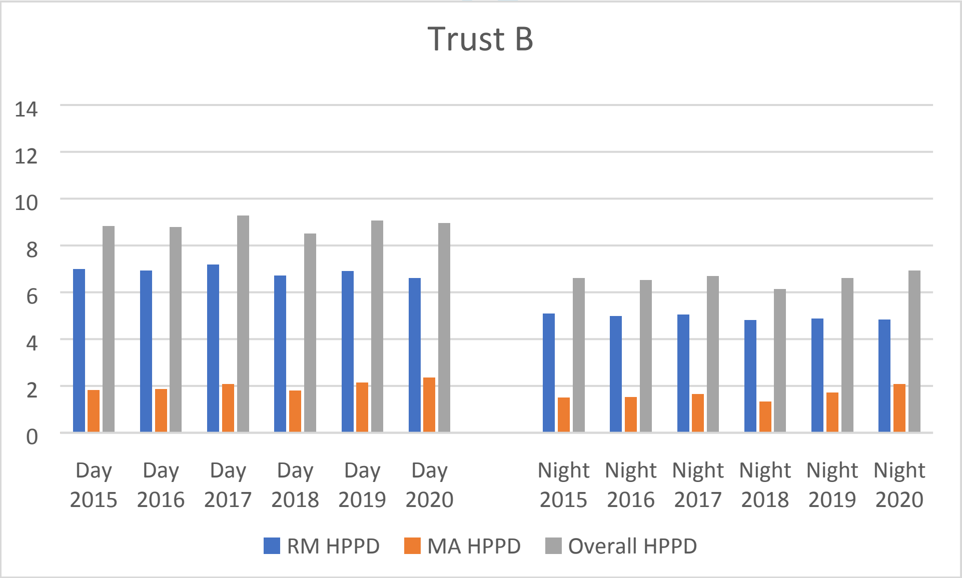

SUPPLEMENTARY INFORMATION

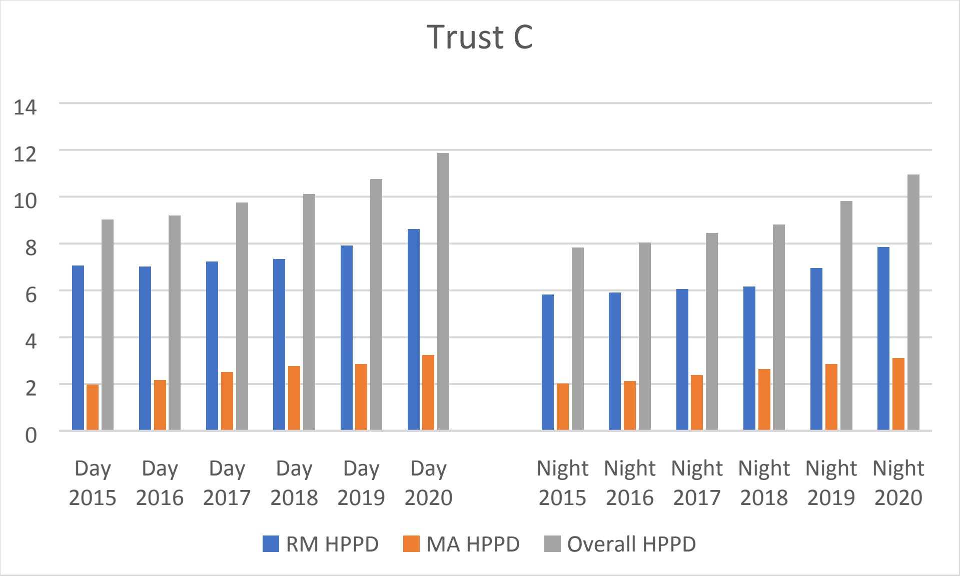

S7 Mean admissions per day and night

	Mean admissions Day	Mean admissions Night
Trust A	3.2	1.7
Trust B	39.0	28.4
Trust C	15.6	13.5

For peer review only - http://bmjopen.bmj.com/site/about/guidelines.xhtml

SUPPLEMENTARY INFORMATION

S8 Univariable analyses

Based on dataset with 64,250 records. Cases leading to birth only and their exposure to staffing and outcomes

S8.1 Age category

7 days

INDEXHRGPostnatalR7days	Odds Ratio	Std. Err.	z	P>|z|	[95% Conf.	Interval]
age_category_combined	1.053196	.0204267	2.67	0.008	1.013912	1.094002
_cons	.0183375	.0055243	-13.27	0.000	.0101604	.0330956
Trustid						
var(_cons)	.229649	.1965479			.0429095	1.229068

INDEXHRGPostnatalR7~s	Odds Ratio	Std. Err.	z	P>|z|	[95% Conf.	Interval]
age_category_combined						
<20 years	1.058031	.1402046	0.43	0.670	.8160215	1.371813
20-24 years	1.048133	.0760309	0.65	0.517	.9092238	1.208264
25-29 years	1	(base)				
30-34 years	1.090576	.0644839	1.47	0.143	.971238	1.224577
35-39 years	1.093644	.0758638	1.29	0.197	.9546186	1.252916
40+years	1.547226	.157508	4.29	0.000	1.267363	1.888888
_cons	.022734	.0064471	-13.34	0.000	.0130403	.0396338
Trustid						
var(_cons)	.2307061	.1974092			.0431229	1.23427

30 days

INDEXHRGPostnatalR30days	Odds Ratio	Std. Err.	z	P>|z|	[95% Conf.	Interval]
age_category_combined	1.063331	.0174322	3.75	0.000	1.029707	1.098052
_cons	.0246987	.0069991	-13.06	0.000	.0141729	.0430416
Trustid						
var(_cons)	.2104974	.1779219			.0401581	1.103369

INDEXHRGPostnatalR3~s	Odds Ratio	Std. Err.	z	P>|z|	[95% Conf.	Interval]
age_category_combined						
<20 years	.9815472	.1122787	-0.16	0.871	.7844092	1.22823
20-24 years	.9876068	.0611014	-0.20	0.840	.8748262	1.114927
25-29 years	1	(base)				
30-34 years	1.081891	.0537382	1.58	0.113	.9815306	1.192513
35-39 years	1.111124	.0643879	1.82	0.069	.9918288	1.244768
40+years	1.435806	.1267896	4.10	0.000	1.207619	1.70711
_cons	.0328283	.0088607	-12.66	0.000	.019342	.055718
Trustid						
var(_cons)	.211206	.1784987			.0403015	1.106856

For peer review only - http://bmjopen.bmj.com/site/about/guidelines.xhtml

SUPPLEMENTARY INFORMATION

S8.2 Mode of birth

7 days

INDEXHRGPostnatalR7days	Odds Ratio	Std. Err.	z	P>|z|	[95% Conf.	Interval]
birth_method	.8493981	.0143447	-9.67	0.000	.8217431	.8779837
_cons	.0437614	.0138256	-9.90	0.000	.0235597	.0812855
Trustid						
var(_cons)	.2852507	.2420273			.0540761	1.504692

INDEXHRGPostnatalR7days	Odds Ratio	Std. Err.	z	P>|z|	[95% Conf.	Interval]
birth_method						
Assisted delivery	1.837988	.1171888	9.55	0.000	1.622073	2.082642
Emergency Caesarean section	1.887994	.1098547	10.92	0.000	1.684506	2.116063
Spontaneous vaginal birth	1	(base)				
Planned caesaren section	1.499737	.1035507	5.87	0.000	1.309915	1.717066
_cons	.0191961	.0061326	-12.37	0.000	.010263	.0359045
Trustid						
var(_cons)	.2976366	.2520993			.0565868	1.565515

30 days

INDEXHRGPostnatalR30days	Odds Ratio	Std. Err.	z	P>|z|	[95% Conf.	Interval]
birth_method	.8565016	.012286	-10.80	0.000	.8327569	.8809234
_cons	.060773	.0182508	-9.33	0.000	.0337354	.1094802
Trustid						
var(_cons)	.2604033	.2187666			.0501818	1.351284

INDEXHRGPostnatalR30days	Odds Ratio	Std. Err.	z	P>|z|	[95% Conf.	Interval]
birth_method						
Assisted birth	1.82806	.1003386	10.99	0.000	1.641609	2.035688
Emergency Caesarean birth	2.04119	.1001662	14.54	0.000	1.854013	2.247263
Spontaneous vaginal birth	1	(base)				
Planned caesaren birth	1.7063	.0971034	9.39	0.000	1.526211	1.907639
_cons	.0262815	.0080849	-11.83	0.000	.0143813	.0480289
Trustid						
var(_cons)	.2778377	.2329243			.0537266	1.43679

For peer review only

SUPPLEMENTARY INFORMATION

S8.3 SHMI risk

7 days

INDEXHRGPostnatalR7days	Odds Ratio	Std. Err.	z	P>|z|	[95% Conf.	Interval]
SHMI_risk	1.554559	.7537043	0.91	0.363	.601056	4.020679
_cons	.0245152	.0068651	-13.24	0.000	.0141603	.0424424
Trustid						
var(_cons)	.2289089	.1959533			.042757	1.225515

30 days

INDEXHRGPostnatalR30days	Odds Ratio	Std. Err.	z	P>|z|	[95% Conf.	Interval]
SHMI_risk	1.546009	.6579018	1.02	0.306	.6714051	3.559915
_cons	.0348519	.0092971	-12.58	0.000	.0206614	.0587886
Trustid						
var(_cons)	.2090766	.1767756			.0398666	1.096483

S8.4 Understaffing by Registered Midwives, Maternity Assistants and Overall staffing compared with expected staffing

RM – 7 and 30 days

INDEXHRGPostnatalR7days	Odds Ratio	Std. Err.	z	P>|z|	[95% Conf.	Interval]
understaffRNless1	1.065489	.0489993	1.38	0.168	.9736534	1.165987
_cons	.0236707	.0067299	-13.17	0.000	.0135582	.0413257
Trustid						
var(_cons)	.2338232	.2000391			.0437192	1.250555

INDEXHRGPostnatalR30days	Odds Ratio	Std. Err.	z	P>|z|	[95% Conf.	Interval]
understaffRNless1	1.04061	.0403364	1.03	0.304	.9644803	1.122748
_cons	.0341239	.0091883	-12.54	0.000	.0201308	.0578436
Trustid						
var(_cons)	.2114666	.1787744			.0403305	1.108791

MA – 7 and 30 days

INDEXHRGPostnatalR7days	Odds Ratio	Std. Err.	z	P>|z|	[95% Conf.	Interval]
understaffNAless1	.9835236	.0455362	-0.36	0.720	.8982039	1.076948
_cons	.0252991	.0067369	-13.81	0.000	.0150119	.0426356
Trustid						
var(_cons)	.2031686	.177073			.0368117	1.121314

INDEXHRGPostnatalR30days	Odds Ratio	Std. Err.	z	P>|z|	[95% Conf.	Interval]
understaffNAless1	.980422	.0383335	-0.51	0.613	.9080964	1.058508
_cons	.0350969	.009577	-12.28	0.000	.0205588	.0599155
Trustid						
var(_cons)	.2164081	.1847274			.0406146	1.153095

For peer review only - http://bmjopen.bmj.com/site/about/guidelines.xhtml

SUPPLEMENTARY INFORMATION

Overall – 7 and 30 days

INDEXHRGPostnatalR7days	Odds Ratio	Std. Err.	z	P>|z|	[95% Conf.	Interval]
understaffOverallLess1	1.054719	.0486384	1.16	0.248	.9635706	1.154489
_cons	.0242992	.0065635	-13.76	0.000	.0143111	.0412583
Trustid						
var(_cons)	.2089796	.1818267			.0379752	1.150027

INDEXHRGPostnatalR30days	Odds Ratio	Std. Err.	z	P>|z|	[95% Conf.	Interval]
understaffOverallLess1	1.040723	.0405168	1.03	0.305	.9642651	1.123242
_cons	.0339218	.0093583	-12.27	0.000	.0197538	.0582513
Trustid						
var(_cons)	.2211271	.1886021			.0415567	1.176637

S8.5 Turnover (admissions + discharges) more than expected

7 days

INDEXHRGPostnatalR7days	Odds Ratio	Std. Err.	z	P>|z|	[95% Conf.	Interval]
HighrerExpectedTurnover	.9720326	.044106	-0.63	0.532	.889319	1.062439
_cons	.0248647	.0069261	-13.26	0.000	.0144038	.0429228
Trustid						
var(_cons)	.2245931	.1924639			.0418759	1.204562

30 days

INDEXHRGPostnatalR30days	Odds Ratio	Std. Err.	z	P>|z|	[95% Conf.	Interval]
HighrerExpectedTurnover	.9662132	.0369906	-0.90	0.369	.8963662	1.041503
_cons	.0355261	.0094323	-12.57	0.000	.021113	.0597782
Trustid						
var(_cons)	.2057703	.174093			.039194	1.080305

S8.6 Skill mix lower than expected

7 days

INDEXHRGPostnatalR7days	Odds Ratio	Std. Err.	z	P>|z|	[95% Conf.	Interval]
LessExpectedSkillMix	1.016522	.0471679	0.35	0.724	.9281541	1.113304
_cons	.0248425	.0066367	-13.83	0.000	.0147162	.0419366
Trustid						
var(_cons)	.2044159	.178052			.0370761	1.127029

30 days

INDEXHRGPostnatalR30days	Odds Ratio	Std. Err.	z	P>|z|	[95% Conf.	Interval]
LessExpectedSkillMix	.979263	.0383599	-0.53	0.593	.9068927	1.057408
_cons	.0351277	.0096161	-12.23	0.000	.0205417	.0600707
Trustid						
var(_cons)	.2177784	.1858425			.0408918	1.159826

For peer review only - http://bmjopen.bmj.com/site/about/guidelines.xhtml

SUPPLEMENTARY INFORMATION

For peer review only - http://bmjopen.bmj.com/site/about/guidelines.xhtml

For peer review only

SUPPLEMENTARY INFORMATION

S9 Full models for staffing below the mean compared to mean staffing (HPPD)

7 days

INDEXHRGPostnatalR7days	Odds Ratio	Std. Err.	z	P>|z|	[95% Conf.	Interval]
age_category_combined						
<20 years	1.0659	0.1490	0.46	0.648	0.8104	1.4019
20-24 years	1.0000	(base)				
25-29 years	0.9135	0.0680	-1.22	0.224	0.7896	1.0570
30-34 years	0.9828	0.0712	-0.24	0.811	0.8528	1.1327
35-39 years	0.9828	0.0803	-0.21	0.832	0.8374	1.1534
40+years	1.3680	0.1528	2.81	0.005	1.0990	1.7029
birth_method						
Assisted birth	1.8728	0.1220	9.63	0.000	1.6484	2.1278
Emergency Caesarean birth	1.9117	0.1136	10.90	0.000	1.7015	2.1480
Spontaneous vaginal birth	1.0000	(base)				
Planned caesaren birth	1.5183	0.1076	5.89	0.000	1.3214	1.7446
understaffRNless1	1.1077	0.0560	2.02	0.043	1.0031	1.2231
understaffNAless1	0.9572	0.0486	-0.86	0.389	0.8664	1.0574
SHMI_risk	0.6869	0.6477	-0.40	0.690	0.1082	4.3602
_cons	0.0189	0.0060	-12.52	0.000	0.0102	0.0353
Trustid						
var(_cons)	0.2795	0.2394			0.0521	1.4980

30 days

INDEXHRGPostnatalR30days	Odds Ratio	Std. Err.	z	P>|z|	[95% Conf.	Interval]
age_category_combined						
<20 years	1.0650	0.1289	0.52	0.603	0.8401	1.3501
20-24 years	1.0000	(base)				
25-29 years	0.9694	0.0617	-0.49	0.625	0.8558	1.0981
30-34 years	1.0237	0.0636	0.38	0.706	0.9064	1.1563
35-39 years	1.0455	0.0726	0.64	0.522	0.9125	1.1980
40+years	1.3175	0.1281	2.84	0.005	1.0889	1.5941
birth_method						
Assisted birth	1.8498	0.1038	10.96	0.000	1.6570	2.0649
Emergency Caesarean birth	2.0769	0.1040	14.60	0.000	1.8828	2.2911
Spontaneous vaginal birth	1.0000	(base)				
Planned caesaren birth	1.7072	0.1001	9.12	0.000	1.5219	1.9151
understaffRNless1	1.0802	0.0461	1.81	0.071	0.9935	1.1745
understaffNAless1	0.9647	0.0414	-0.84	0.402	0.8868	1.0494
SHMI_risk	0.8868	0.5874	-0.18	0.856	0.2421	3.2480
_cons	0.0246	0.0079	-11.49	0.000	0.0131	0.0463
Trustid						
var(_cons)	0.2956	0.2494			0.0566	1.5448

For peer review only

For peer review only - http://bmjopen.bmj.com/site/about/guidelines.xhtml

SUPPLEMENTARY INFORMATION

S10. Staffing expressed as absolute values : number of individuals per midwife / maternity care assistant

Full model with individuals per midwife/maternity assistant as staffing variable

7 days

INDEXHRGPostnatalR7days	Odds Ratio	Std. Err.	z	P>|z|	[95% Conf.	Interval]
age_category_combined						
<20 years	1.0673	0.1492	0.47	0.641	0.8115	1.4037
20-24 years	1.0000	(base)				
25-29 years	0.9131	0.0679	-1.22	0.222	0.7892	1.0564
30-34 years	0.9831	0.0712	-0.24	0.814	0.8531	1.1330
35-39 years	0.9831	0.0803	-0.21	0.835	0.8377	1.1537
40+years	1.3675	0.1527	2.80	0.005	1.0986	1.7022
birth_method						
Assisted birth	1.8729	0.1220	9.64	0.000	1.6485	2.1279
Emergency Caesarean birth	1.9118	0.1136	10.90	0.000	1.7015	2.1480
Spontaneous vaginal birth	1.0000	(base)				
Planned caesaren birth	1.5185	0.1076	5.89	0.000	1.3215	1.7448
individuals_per_midwife	1.0630	0.0553	1.17	0.240	0.9599	1.1772
individuals_per_MAssistant	0.9978	0.0100	-0.21	0.830	0.9784	1.0177
SHMI_risk	0.6913	0.6504	-0.39	0.695	0.1094	4.3702
_cons	0.0164	0.0053	-12.63	0.000	0.0087	0.0310

30 days

INDEXHRGPostnatalR30days	Odds Ratio	Std. Err.	z	P>|z|	[95% Conf.	Interval]
age_category_combined						
<20 years	1.0661	0.1290	0.53	0.597	0.8410	1.3514
20-24 years	1.0000	(base)				
25-29 years	0.9693	0.0617	-0.49	0.624	0.8557	1.0980
30-34 years	1.0245	0.0636	0.39	0.697	0.9071	1.1571
35-39 years	1.0462	0.0727	0.65	0.515	0.9131	1.1988
40+years	1.3167	0.1280	2.83	0.005	1.0882	1.5931
birth_method						
Assisted birth	1.8494	0.1038	10.95	0.000	1.6567	2.0645
Emergency Caesarean birth	2.0769	0.1040	14.60	0.000	1.8828	2.2911
Spontaneous vaginal birth	1.0000	(base)				
Planned caesaren birth	1.7056	0.1000	9.11	0.000	1.5204	1.9133
individuals_per_midwife	1.0129	0.0448	0.29	0.772	0.9288	1.1045
individuals_per_MAssistant	1.0022	0.0084	0.26	0.796	0.9859	1.0187
SHMI_risk	0.8889	0.5886	-0.18	0.859	0.2428	3.2548
_cons	0.0237	0.0079	-11.20	0.000	0.0123	0.0457
Trustid						
var(_cons)	0.2746	0.2343			0.0516	1.4616

For peer review only - http://bmjopen.bmj.com/site/about/guidelines.xhtml

SUPPLEMENTARY INFORMATION

S11 Additional analysis : High and low staffing categories compared with mean staffing

7 days

INDEXHRGPostnatalR7days	Odds Ratio	Std. Err.	z	P>|z|	[95% Conf.	Interval]
age_category_combined						
<20 years	1.0678	0.1493	0.47	0.639	0.8118	1.4044
20-24 years	1.0000	(base)				
25-29 years	0.9137	0.0680	-1.21	0.225	0.7897	1.0572
30-34 years	0.9843	0.0713	-0.22	0.827	0.8541	1.1343
35-39 years	0.9836	0.0803	-0.20	0.839	0.8381	1.1543
40+years	1.3703	0.1531	2.82	0.005	1.1009	1.7058
birth_method						
Assisted delivery	1.8725	0.1220	9.63	0.000	1.6481	2.1275
Emergency Caesarean section	1.9110	0.1136	10.89	0.000	1.7008	2.1472
Spontaneous vaginal birth	1.0000	(base)				
Planned caesaren section	1.5177	0.1076	5.89	0.000	1.3208	1.7438
StaffCatRN						
Low, 95% of mean or less	1.0562	0.0609	0.95	0.343	0.9434	1.1825
Near to mean 95%-105%	1.0000	(base)				
High 105% of mean or more	0.9730	0.0600	-0.44	0.657	0.8622	1.0980
StaffCatNA						
Low, 95% of mean or less	0.9239	0.0584	-1.25	0.211	0.8162	1.0458
Near to mean 95%-105%	1.0000	(base)				
High 105% of mean or more	0.9346	0.0597	-1.06	0.289	0.8246	1.0592
SHMI_risk	0.6935	0.6485	-0.39	0.695	0.1109	4.3353
_cons	0.0205	0.0066	-12.17	0.000	0.0110	0.0384
Trustid						
var(_cons)	0.2779	0.2382			0.0518	1.4913

30 days

INDEXHRGPostnatalR30days	Odds Ratio	Std. Err.	z	P>|z|	[95% Conf.	Interval]
age_category_combined						
<20 years	1.0660	0.1290	0.53	0.598	0.8409	1.3513
20-24 years	1.0000	(base)				
25-29 years	0.9698	0.0617	-0.48	0.630	0.8562	1.0986
30-34 years	1.0250	0.0637	0.40	0.692	0.9075	1.1576
35-39 years	1.0463	0.0727	0.65	0.515	0.9131	1.1988
40+years	1.3185	0.1282	2.84	0.004	1.0897	1.5954
birth_method						
Assisted delivery	1.8497	0.1038	10.96	0.000	1.6570	2.0649
Emergency Caesarean section	2.0760	0.1039	14.59	0.000	1.8819	2.2900
Spontaneous vaginal birth	1.0000	(base)				
Planned caesaren section	1.7054	0.1000	9.10	0.000	1.5203	1.9131
StaffCatRN						
Low, 95% of mean or less	1.0016	0.0487	0.03	0.973	0.9105	1.1018
Near to mean 95%-105%	1.0000	(base)				
High 105% of mean or more	0.9574	0.0496	-0.84	0.401	0.8649	1.0598
StaffCatNA						
Low, 95% of mean or less	0.9679	0.0521	-0.61	0.545	0.8710	1.0757
Near to mean 95%-105%	1.0000	(base)				
High 105% of mean or more	0.9675	0.0526	-0.61	0.543	0.8696	1.0763
SHMI_risk	0.8887	0.5875	-0.18	0.858	0.2432	3.2470
_cons	0.0263	0.0085	-11.27	0.000	0.0140	0.0495
Trustid						
var(_cons)	0.2919	0.2464			0.0558	1.5271

For peer review only - http://bmjopen.bmj.com/site/about/guidelines.xhtml

SUPPLEMENTARY INFORMATION

For peer review only

STROBE Statement—Checklist of items that should be included in reports of ***cohort studies***

	Item No	Recommendation	Page No
**Title and abstract**	1	(*a*) Indicate the study's design with a commonly used term in the title or the abstract	1
		(*b*) Provide in the abstract an informative and balanced summary of what was done and what was found	1
**Introduction**			
Background/rationale	2	Explain the scientific background and rationale for the investigation being reported	3
Objectives	3	State specific objectives, including any prespecified hypotheses	3
**Methods**			
Study design	4	Present key elements of study design early in the paper	3
Setting	5	Describe the setting, locations, and relevant dates, including periods of recruitment, exposure, follow-up, and data collection	3-4
Participants	6	(*a*) Give the eligibility criteria, and the sources and methods of selection of participants. Describe methods of follow-up<br>(*b*) For matched studies, give matching criteria and number of exposed and unexposed	3-4
Variables	7	Clearly define all outcomes, exposures, predictors, potential confounders, and effect modifiers. Give diagnostic criteria, if applicable	4
Data sources/ measurement	8*	For each variable of interest, give sources of data and details of methods of assessment (measurement). Describe comparability of assessment methods if there is more than one group	4
Bias	9	Describe any efforts to address potential sources of bias	4
Study size	10	Explain how the study size was arrived at	4
Quantitative variables	11	Explain how quantitative variables were handled in the analyses. If applicable, describe which groupings were chosen and why	4-5
Statistical methods	12	(*a*) Describe all statistical methods, including those used to control for confounding<br>(*b*) Describe any methods used to examine subgroups and interactions<br>(*c*) Explain how missing data were addressed<br>(*d*) If applicable, explain how loss to follow-up was addressed<br>(*e*) Describe any sensitivity analyses	5 and supplement
**Results**			
Participants	13*	(a) Report numbers of individuals at each stage of study—eg numbers potentially eligible, examined for eligibility, confirmed eligible, included in the study, completing follow-up, and analysed<br>(b) Give reasons for non-participation at each stage<br>(c) Consider use of a flow diagram	5
Descriptive data	14*	(a) Give characteristics of study participants (eg demographic, clinical, social) and information on exposures and potential confounders	5
		(b) Indicate number of participants with missing data for each variable of interest	Supplement
		(c) Summarise follow-up time (eg, average and total amount)	5
Outcome data	15*	Report numbers of outcome events or summary measures over time	5

For peer review only

Main results	16	(*a*) Give unadjusted estimates and, if applicable, confounder-adjusted estimates and their precision (eg, 95% confidence interval). Make clear which confounders were adjusted for and why they were included	6
		(*b*) Report category boundaries when continuous variables were categorized	8
		(*c*) If relevant, consider translating estimates of relative risk into absolute risk for a meaningful time period	7
Other analyses	17	Report other analyses done—eg analyses of subgroups and interactions, and sensitivity analyses	7 and supplement
**Discussion**			
Key results	18	Summarise key results with reference to study objectives	8
Limitations	19	Discuss limitations of the study, taking into account sources of potential bias or imprecision. Discuss both direction and magnitude of any potential bias	9
Interpretation	20	Give a cautious overall interpretation of results considering objectives, limitations, multiplicity of analyses, results from similar studies, and other relevant evidence	9
Generalisability	21	Discuss the generalisability (external validity) of the study results	9
**Other information**			
Funding	22	Give the source of funding and the role of the funders for the present study and, if applicable, for the original study on which the present article is based	In submission portal

*Give information separately for exposed and unexposed groups.

Note: An Explanation and Elaboration article discusses each checklist item and gives methodological background and published examples of transparent reporting. The STROBE checklist is best used in conjunction with this article (freely available on the Web sites of PLoS Medicine at http://www.plosmedicine.org/, Annals of Internal Medicine at http://www.annals.org/, and Epidemiology at http://www.epidem.com/). Information on the STROBE Initiative is available at http://www.strobe-statement.org.

For peer review only

For peer review only - http://bmjopen.bmj.com/site/about/guidelines.xhtml

BMJ Open

Inpatient midwifery staffing levels and postpartum readmissions – a retrospective multi-centre longitudinal study

Journal:	*BMJ Open*
Manuscript ID	bmjopen-2023-077710.R1
Article Type:	Original research
Date Submitted by the Author:	30-Jan-2024

Complete List of Authors:	Turner, Lesley; University of Southampton, School of Health Sciences<br>Saville, Christina; University of Southampton, School of Health Sciences<br>Ball, Jane; University of Southampton, School of Health Sciences<br>Culliford, David; NIHR Applied Research Collaboration Wessex; University of Southampton, School of Health Sciences<br>Dall'Ora, Chiara; University of Southampton, School of Health Sciences<br>Jones, Jeremy; University of Southampton, School of Health Sciences<br>Kitson-Reynolds, Ellen; University of Southampton, School of Health Sciences<br>Meredith, Paul; University of Southampton, School of Health Sciences<br>Griffiths, Peter; University of Southampton, School of Health Sciences; National Institute for Health Research Applied Research Centre (Wessex)

**Primary Subject Heading**:	Obstetrics and gynaecology
Secondary Subject Heading:	Health services research
Keywords:	OBSTETRICS, Nursing Care, Postpartum Period, Human resource management < HEALTH SERVICES ADMINISTRATION & MANAGEMENT

SCHOLARONE™
Manuscripts

I, the Submitting Author has the right to grant and does grant on behalf of all authors of the Work (as defined in the below author licence), an exclusive licence and/or a non-exclusive licence for contributions from authors who are: i) UK Crown employees; ii) where BMJ has agreed a CC-BY licence shall apply, and/or iii) in accordance with the terms applicable for US Federal Government officers or employees acting as part of their official duties; on a worldwide, perpetual, irrevocable, royalty-free basis to BMJ Publishing Group Ltd ("BMJ") its licensees and where the relevant Journal is co-owned by BMJ to the co-owners of the Journal, to publish the Work in this journal and any other BMJ products and to exploit all rights, as set out in our licence.

The Submitting Author accepts and understands that any supply made under these terms is made by BMJ to the Submitting Author unless you are acting as an employee on behalf of your employer or a postgraduate student of an affiliated institution which is paying any applicable article publishing charge ("APC") for Open Access articles. Where the Submitting Author wishes to make the Work available on an Open Access basis (and intends to pay the relevant APC), the terms of reuse of such Open Access shall be governed by a Creative Commons licence – details of these licences and which Creative Commons licence will apply to this Work are set out in our licence referred to above.

Other than as permitted in any relevant BMJ Author's Self Archiving Policies, I confirm this Work has not been accepted for publication elsewhere, is not being considered for publication elsewhere and does not duplicate material already published. I confirm all authors consent to publication of this Work and authorise the granting of this licence.

For peer review only

Inpatient midwifery staffing levels and postpartum readmissions – a retrospective multi-centre longitudinal study

Lesley Turner*, School of Health Sciences, University of Southampton, Southampton, England, SO17 1BJ

*Corresponding author : Lesley Turner
Email lyt1g19@soton.ac.uk

Christina Saville, School of Health Sciences, University of Southampton, Southampton, England

Jane Ball, School of Health Sciences, University of Southampton, Southampton, England

David Culliford, NIHR Applied Research Collaboration Wessex, School of Health Sciences, University of Southampton, Southampton, England

Chiara Dall'ora, School of Health Sciences, University of Southampton, Southampton, England

Jeremy Jones, School of Health Sciences, University of Southampton, Southampton, England

Ellen Kitson-Reynolds, School of Health Sciences, University of Southampton, Southampton, England

Paul Meredith, School of Health Sciences, University of Southampton, Southampton, England

Peter Griffiths, School of Health Sciences, University of Southampton, National Institute for Health Research Applied Research Centre (Wessex), Southampton, England

Keywords : readmission, post-partum, human resource management

Word count - excluding title page, references, figures and tables 3759 words

For peer review only - http://bmjopen.bmj.com/site/about/guidelines.xhtml

Inpatient midwifery staffing levels and postpartum readmissions – a retrospective multi-centre longitudinal study

Abstract

Background

Preventing readmission to hospital after giving birth is a key priority, as rates have been rising along with associated costs. There are many contributing factors to readmission, and some are thought to be preventable. Nurse and midwife understaffing has been linked to deficits in care quality. This study explores the relationship between staffing levels and readmission rates in maternity settings.

Methods

We conducted a retrospective longitudinal study using routinely collected individual patient data in three maternity services in England from 2015-2020. Data on admissions, discharges and case-mix were extracted from hospital administration systems. Staffing and workload were calculated in Hours Per Patient day per shift in the first two 12-hour shifts of the index (birth) admission. Postpartum readmissions and staffing exposures for all birthing admissions were entered into a hierarchical multivariable logistic regression model to estimate the odds of readmission when staffing was below the mean level for the maternity service.

Results

64,250 maternal admissions resulted in birth and 2903 mothers were readmitted within 30 days of discharge (4.5%). Absolute levels of staffing ranged between 2.3 and 4.1 individuals per midwife in the three services. Below average midwifery staffing was associated with higher rates of postpartum readmissions within 7 days of discharge (aOR 1.108, 95% CI 1.003, 1.223). The effect was smaller and not statistically significant for readmissions within 30 days of discharge (aOR 1.080, 95% CI 0.994, 1.174). Below average maternity assistant staffing was associated with lower rates of postpartum readmissions (7 days, aOR 0.957, 95% CI 0.867, 1.057; 30 days aOR 0.965, 95% CI 0.887, 1.049, both not statistically significant).

Conclusion

We found evidence that lower than expected midwifery staffing levels is associated with more postpartum readmissions. The nature of the relationship requires further investigation including examining potential mediating factors and reasons for readmission in maternity populations.

For peer review only - http://bmjopen.bmj.com/site/about/guidelines.xhtml

List of abbreviations

aOR	Adjusted odds ratio
NHS	National Health Service
HRG	Healthcare Resource Group
SHMI	Standardised Hospital Mortality Indicator
HPPD	Hours Per Patient Day
RM	Registered Midwife
MA	Maternity Assistant
AIC	Akaike information criterion
BIC	Bayesian information criterion

Strengths and Limitations of this study

Longitudinal design with individual level linking of staffing exposure to subsequent readmissions.

Adjustment for case mix, staff groups and organisational level variables using hierarchical multivariable modelling.

Staffing was measured in the first two 12-hour intervals of the index (birth) hospital stay and did not account for the exposure to staffing for the whole hospital stay.

Considers exposure to staffing in the whole maternity service rather than close to women at ward level.

For peer review only

For peer review only - http://bmjopen.bmj.com/site/about/guidelines.xhtml

Background

In many countries of the world the majority of mothers give birth in hospital[1]. Preventing readmission due to potentially avoidable complications is a key priority, not only because of the negative experience and burden on the women affected[2], but because readmissions are costly[3]. Readmission rates are used as an indicator of the quality of care[4], and in some countries providers are held financially accountable for patient outcomes during the first 30 days after discharge for many patient groups[5]. Rates of postpartum readmission vary by country, with reported rates of 1.0% in the USA[6], 1.7% in Sweden[7] and 3.3% in England and Wales[8].

Reports from England and the USA suggest that readmission rates have increased in recent years[8, 9]. This coincides with an increase in caesarean birth rates[10]. In England 4.3% of women having a caesarean birth are readmitted (1 in 23) compared with 2.9% of those having a vaginal birth (1 in 34)[8]. The recent trend towards shorter length of hospital stay and reduced community provision may also contribute to increased readmission rates. However, whilst some research has pointed to an association between reduced length of stay and increased readmission rates [11, 12] this finding is not consistent in the research literature[13, 14]. The most common reasons for readmission include wound complications, post-partum haemorrhage, hypertension, mastitis and thromboembolism [6, 9, 10, 15, 16]. Postpartum readmissions typically occur within the first 14-20 days after discharge[17, 18].

Pressures on staffing within maternity services have been reported globally[19] but the consequences of this are poorly understood. In many areas of health care there is strong evidence that lower nurse staffing levels are associated with reduced quality of care and poorer patient outcomes (e.g. as measured by mortality[20], adverse events[21] and patient satisfaction)[22]. Studies in medical and surgical inpatient settings have found that higher registered nurse staffing levels are associated with reduced readmission rates[23-25]. A scoping review of midwifery and nurse staffing for inpatient maternity services[26] found just two studies which measured maternal readmission in relation to staffing. These studies found that readmission rates were lower when more midwifery staff were employed[27] and the proportion of registered staff increased[28]. More evidence is needed on the relationship between maternity staffing levels, skill mix and readmission rates to understand whether previous findings are replicated and to understand the size of the effect if an association exists.

For peer review only

The aim of this study is to assess the relationship between individual patient-level exposure to staffing and postpartum readmission rates. A longitudinal design was adopted to enable women's exposure to staffing to be aligned to their outcomes, adjusting for individual risk factors.

Methods

We conducted a retrospective longitudinal study using individual patient records in multiple centres[29]. Maternity inpatient stays from 13th April 2015- 29th February 2020 were extracted from the hospital Patient Administration System for 3 Acute National Health Service (NHS) Trusts in England. Data after February 2020 was not studied due to service changes during the covid pandemic. NHS Trusts comprise one or more hospitals operating as a group under a combined management. Two of the Trusts had inpatient services all on one site (antenatal, postnatal and labour ward). The third Trust had two geographical sites, comprising two maternity hospitals.

The available data included all women admitted to maternity wards, including those admitted via Day Assessment triage unit, totalling 113,002 admissions. Patient data was extracted for the date and time of admissions and discharges per maternity patient episode during the study period. The patient pseudonyms and dates were used to identify which postpartum women had been readmitted to the same maternity service for any cause within 7 days and 30 days of discharge. The primary reason for readmission was extracted from the ICD-10 code for the readmission episode. To account for variation in case mix and comorbidities we used the age band on admission, mode of birth from the Healthcare Resource Group (HRG) procedure code[30], and the Standardised Hospital Mortality Indicator (SHMI) which calculates an age specific risk score based on comorbidities for a given reason for admission ('diagnosis')[31, 32].HRGs are standard groupings of clinically similar treatments which use comparable levels of healthcare resource. Admissions without a birth were excluded from the final data set as they would not be eligible to have a postpartum readmission.

Shift-level staffing data was obtained from Trust electronic roster systems which recorded all worked shifts and the grades of staff. This data comprised the time worked excluding breaks for the Registered Midwife group and Maternity Assistant Staff group and was matched to 12 hour periods, either 07.00 to 19.00 or 1900 to 07.00 to determine the staff time available in each time period. Staffing was measured by Hours Per Patient Day (HPPD) and this variable was generated by dividing the total worked time for the Staff group by the total ward occupancy as detailed in the measure

For peer review only - http://bmjopen.bmj.com/site/about/guidelines.xhtml

guidance document[33].. All admitted individuals were included in the occupancy measure because they all contribute to workload, with neonates accounting for 40% of the admitted population. The HPPD was calculated for Registered Midwife (RM) and Maternity Assistant (MA) staff separately, and the staff groups were combined as a measure of Overall staffing per 12-hour period per day. Skill mix was calculated per shift using the RM worked seconds as a proportion of the total worked seconds, which encompassed RMs and MAs. Registered Nurses were included in the Registered Midwife totals as there were very few (<0.1%) and were of a similar grade to midwives. Data was cleaned to remove shifts where patient occupancy is zero seconds, and to remove shifts with RM HPPD outliers (defined as RM HPPD < 0.5 or RM HPPD > 48).

Staffing for only the index (birth) admission prior to the postpartum readmission was recorded. For the analysis, staffing was noted in the first two 12-hour periods of care in the Index admission (one interval of day shift and one of night shift). This method avoids recording staffing exposure for increased length of stay for a minority of complex cases. Longer time periods will skew the analysis as there is more opportunity to be exposed to understaffing. We defined the expected staffing as the mean HPPD for the maternity service in each Trust, and this was calculated separately for both day and night shifts. Each of the two 12-hour intervals of observed staffing (HPPD) was divided by expected staffing for the same period (HPPD) and these ratios were averaged for the first two 12 hour intervals, with values below 1 indicating a reduction in staff compared with expected levels. The HPPD was converted into the number individuals cared for per midwife and individuals per maternity assistant by dividing 24 hours by the HPPD for each group. This allows service planners to see the potential differences of altering staffing levels in absolute terms using variables familiar to them. A further measure of ward activity and workload was calculated. The variable 'turnover' equalled the total number of admissions plus discharges per Trust per 12 hour interval. Transfers between wards were not included in this measure. Mean turnover was calculated per Trust and variables were created to represent higher than expected turnover compared to the Trust mean, by day and night shifts.

Univariable analyses on the relationship between independent variables and readmissions were performed, nested in hospital Trust. Multi-level logistic regression was then undertaken with the primary outcomes of postpartum readmission within 7 days and 30 days of discharge, nested in hospital Trust. Readmissions within 7 days was included as this may be more sensitive to variation in care quality during index admissions[34, 35], and longer time intervals increase the chance that

For peer review only - http://bmjopen.bmj.com/site/about/guidelines.xhtml

readmission may be due to outpatient management[36]. The null model was the starting point and variables were added firstly based on indicators in the literature to suggest a potential effect on readmission (age, mode of birth), then staffing variables of interest (exposure to RM and MA staffing below the mean in the main analysis) and then remaining variables of SHMI risk, skill mix and turnover were added in sequence and only retained in the full model if they improved model fit using Akaike information criterion (AIC) and Bayesian information criterion (BIC) values (see supplement S1 for model fit using this forward selection process). After the model fitting, only age, mode of birth and SHMI risk remained in the full model along with the staffing variables. For ease of interpretation, we repeated the full model using absolute values of staffing expressed as the number of individuals cared for by each midwife and maternity assistant. We performed a secondary analysis comparing low and high levels of staffing compared to near mean staffing, classified as 95% to 105% of the mean, to explore non-linear relationships.

All analyses were conducted in Stata (Release 16. College Station, TX: StataCorp LLC). Ethics permission was gained via the University of Southampton Ethics Committee ERGO 52957 and IRAS 128056. This study was funded by the National Institute for Health Research, Health Services & Delivery Research programme, award 128056.

Patient and Public Involvement

This study had service user and clinical staff consultation when selecting the outcome measures of interest, and a lay member contributed to the project steering group.

For peer review only

Results

There were 64,250 maternal admissions that led to a birth between mid-April 2015 and the end of February 2020. The Trusts differed in size with 5,260, 45,518 and 13,472 admissions leading to birth in each of the Trusts respectively. In the cohort, 58.1% of women had a spontaneous vaginal birth, 12.9% assisted instrumental birth, 12.3% planned caesarean birth and 16.7% emergency caesarean birth (Table 1).

Table 1 : Characteristics of the study population and their exposure to staffing

Age		
<20 years	2099	(3.27%)
20-24 years	9,862	(15.35%)
25-29 years	18,362	(28.58%)
30-34 years	20,114	(31.31%)
35-39 years	11,016	(17.15%)
40+ years	2,797	(4.35%)
Mode of Birth		
Assisted birth	7,764	(12.88%)
Emergency Caesarean birth	10,041	(16.66%)
Spontaneous vaginal birth	35,040	(58.14%)
Planned Caesarean birth	7,425	(12.32%)
SHMI	Mean	0.0035
	Std. Dev.	0.0319
Turnover (admissions plus discharges per day)	Mean	56.716
	Std. Dev.	25.397
Registered midwife staffing exposure (HPPD)	Mean	6.640
	Std. Dev.	2.351
Maternity assistant staffing exposure (HPPD)	Mean	2.146
	Std. Dev.	1.045
Skill mix (registered midwives as proportion of total staff)	Mean	0.757
	Std. Dev.	0.039
Readmitted within 30 days of discharge	2903	(4.52%)
Readmitted within 7 days of discharge	2043	(3.18%)

SHMI Standardised Hospital Mortality Indicator HPPD Hours Per Patient Day

Two thousand nine hundred and three women who gave birth were readmitted postnatally within 30 days of discharge (4.5%). Of those readmitted within 30 days, 70% were readmitted within the first 7 days and 89% within 14 days. Rates of readmission differed by birth method: 3.7% of women having spontaneous vaginal birth were readmitted, and rates were 5.9% for planned caesarean birth, 6.3% for assisted birth and 6.8% for emergency caesarean birth respectively. The postpartum readmission rates following birth varied by Trust and were 1.8%, 4.9% and 4.3%. The mean length of stay for postpartum readmissions was 45.4 hours (median 22.5 hrs, IQR 3 hrs to 52 hrs). Infection/sepsis was the most common primary reason for readmission (677/2903, 23.3%). Postpartum haemorrhage accounted for 316/2903 readmissions (10.9%). Some readmissions

For peer review only

appeared to be healthy mothers accompanying their newborn, although this represented only 125/2903 (4.3%) of maternal readmissions (supplement S2).

Staffing varied by Trust for those in our study cohort, with an average of 2.3, 3.6 and 4.1 individuals per midwife in the three services (this equates to 11.7, 6.9 and 5.9 HPPD respectively). More staff were available on day shifts compared with night shifts. The proportion of RM hours per Trust varies from 70.7%% to 77.4% between the Trusts, and almost all the registered staff are classified as midwives where this data is available (supplements S3-S5).

For two of the Trusts the mean overall staffing in HPPD has shown an increase during the 5-year study period, whereas in the third Trust there is a stable level of overall staffing but a slight decrease in RM staff and increase in MA staffing over this period (approx. 4% change in each group). There was more ward activity in terms of admissions and discharges on day shifts compared with night shifts (supplements S6-S7).

Univariable analyses on covariates and postpartum readmission

Age and the mode of birth were statistically significant predictors of postpartum readmission in univariable analyses, with older mothers and those having assisted or operative birth at higher risk (see Table 2 and supplement S8). Higher SHMI risk was associated with higher risk of readmission, but this was not statistically significant. Women exposed to below average RM staffing and overall staffing had increased odds for readmission, but this was not statistically significant. Having more admissions and discharges than expected and a more diluted skill mix than average was not associated with increased postpartum readmissions at 7 days and 30 days.

For peer review only

Table 2: Univariable analyses of Readmission at 7 days and at 30 days from discharge

	Readmitted within 7 days			Readmitted within 30 days		
	OR	95% CI		OR	95% CI	
Age category						
<20 years	1.009	0.770	1.323	0.994	0.787	1.256
20-24 years	1.000	reference category		1.000	reference category	
25-29 years	0.954	0.828	1.100	1.013	0.897	1.143
30-34 years	1.040	0.906	1.195	1.095	0.973	1.233
35-39 years	1.043	0.893	1.219	1.125	0.986	1.284
40+ years	1.476	1.193	1.827	1.454	1.208	1.750
Mode of birth						
Assisted birth	1.838	1.622	2.083	1.828	1.642	2.036
Emergency Caesarean birth	1.888	1.685	2.116	2.041	1.854	2.247
Spontaneous vaginal birth	1.000	reference category			reference category	
Planned Caesarean birth	1.500	1.310	1.717	1.706	1.526	1.908
Registered Midwife staffing <mean	1.065	0.974	1.166	1.041	0.965	1.123
Maternity Assistant staffing <mean	0.984	0.898	1.077	0.980	0.908	1.059
Overall staffing <mean	1.054	0.964	1.154	1.041	0.964	1.123
SHMI risk	1.554	0.601	4.021	1.546	0.671	3.560
Turnover > mean	0.972	0.889	1.062	0.966	0.896	1.042
Skill mix <mean	1.017	0.928	1.113	0.979	0.907	1.057

SHMI Standardised Hospital Mortality Indicator

Relationship between staffing and postpartum readmission in multivariable models

Model fit was improved from the null model using a forward selection procedure with the addition of age category, mode of birth, RM and MA staffing and SHMI risk in that order. It was not improved with the addition of turnover and skill mix variables and therefore these variables are not shown in the full model. The variance inflation factors were below 2 for all variables which suggests that they are not collinear. Missing data is minimal for the variables studied (supplement S1).

Lower than expected midwifery staffing was associated with an 11% higher odds of postpartum readmissions within 7 days of discharge (adjusted OR 1.108, 95% CI 1.003, 1.223). The effect was smaller and not statistically significant for readmissions within 30 days of discharge (adjusted OR 1.080, 95% CI 0.994, 1.175). Lower than expected maternity assistant staffing was associated with a lower odds of readmission at 7 days (OR 0.957, 95% CI 0.866, 1.057) and at 30 days (OR 0.965, 95% CI 0.887, 1.049). These relationships were not statistically significant in the multivariable model (see Table 3 and supplement S9).

For peer review only

For peer review only - http://bmjopen.bmj.com/site/about/guidelines.xhtml

Table 3 : 7 day and 30 day readmissions in the full multivariable models (staffing relative to the mean per service, supplement S9).

Variable	OR readmission 7 days	95% CI		OR readmission 30 days	95% CI	
<20 years	1.066	0.810	1.402	1.065	0.840	1.350
20-24 years	1.000	reference category		1.000	reference category	
25-29 years	0.914	0.790	1.057	0.969	0.856	1.098
30-34 years	0.983	0.853	1.133	1.024	0.906	1.156
35-39 years	0.983	0.837	1.153	1.046	0.913	1.198
40+ years	1.368	1.099	1.703	1.318	1.089	1.594
Assisted birth	1.873	1.648	2.128	1.850	1.657	2.065
Emergency Caesarean birth	1.912	1.701	2.148	2.077	1.883	2.291
Spontaneous vaginal birth	1.000	reference category		1.000	reference category	
Planned Caesarean birth	1.518	1.321	1.745	1.707	1.522	1.915
Exposed to staffing below mean Registered midwives (HPPD)	1.108	1.003	1.223	1.080	0.994	1.174
Exposed to staffing below mean Maternity assistants (HPPD)	0.957	0.866	1.057	0.965	0.887	1.049
SHMI risk	0.687	0.108	4.360	0.8868	0.2421	3.2480

SHMI Standardised Hospital Mortality Indicator HPPD Hours Per Patient Day

In models using absolute (rather than relative) staffing levels, an increase of one individual (mother or baby) per midwife was associated with a 6% increase in the odds of readmission at 7 days, aOR1.063 (95% CI 0.960, 1.177). There was no association with maternity assistant staffing (aOR 0.998, 95% CI 0.978, 1.018). Neither relationship was statistically significant (supplement 10)

Staffing was examined in categories to capture higher than average staffing as well as lower than average levels for readmissions within 7 and 30 days (Table 4, supplement 11). When grouped as three categories, higher midwifery staffing (105% of mean or more) was associated with reduced odds of readmission compared with the category containing the mean. The category of lower than mean midwifery staffing (95% of mean or lower) was associated with increased odds of readmission. None of the relationships in the categorical analyses were statistically significant.

For peer review only - http://bmjopen.bmj.com/site/about/guidelines.xhtml

Table 4 : Multivariable analysis with staffing in categories above and below mean levels for readmissions within 7 days and 30 days of discharge

Variable	OR readmission 7 days	95% CI		OR readmission 30 days	95% CI	
<20 years	1.068	0.812	1.404	1.066	0.841	1.351
20-24 years	1.000	reference category		1.000	reference category	
25-29 years	0.914	0.790	1.057	0.970	0.856	1.099
30-34 years	0.984	0.854	1.134	1.025	0.908	1.158
35-39 years	0.984	0.838	1.154	1.046	0.913	1.199
40+ years	1.370	1.101	1.706	1.319	1.090	1.595
Assisted birth	1.873	1.648	2.127	1.850	1.657	2.065
Emergency Caesarean birth	1.911	1.701	2.147	2.076	1.882	2.290
Spontaneous vaginal birth	1.000	reference category		1.000	reference category	
Planned Caesarean birth	1.518	1.321	1.744	1.705	1.520	1.913
Midwifery staffing						
Low, 95% of mean or less	1.056	0.943	1.182	1.002	0.911	1.102
Near to mean 95%-105%	1.000	reference category		1.000	reference category	
High 105% of mean or more	0.973	0.862	1.098	0.957	0.865	1.060
Maternity assistant staffing						
Low, 95% of mean or less	0.924	0.816	1.046	0.968	0.871	1.076
Near to mean 95%-105%	1.000	reference category		1.000	reference category	
High 105% of mean or more	0.935	0.825	1.059	0.968	0.870	1.077
SHMI risk	0.693	0.111	4.335	0.889	0.243	3.247

SHMI Standardised Hospital Mortality Indicator See supplements S11 and S12

Discussion

In this longitudinal study we analysed individual patient stays and concurrent staff rosters, and we found an association between midwifery staffing below the mean level and higher rates of postpartum readmission. This effect was stronger and statistically significant for readmissions within 7 days of discharge, with an increase in odds of 11% for understaffing compared with the organisation mean staffing levels. We estimate that if a midwife's workload is increased by one additional person, the odds of readmission within 7 days increases by 6.3%, although confidence intervals are wide and cross the null effect in this analysis of absolute staffing data. The direction of findings is in line with that of Gerova[27] who found that increased number of full-time-equivalent midwives in a service was associated with reduced postpartum readmissions. These findings are also seen in the nursing literature, in medical, surgical and paediatric settings[23, 37].

The mean 30-day readmission rate in this study was 4.5% which is higher than rates reported in USA[6] and Sweden[7] where reports are between 1-2%. This difference may reflect service variability such as community provision and the threshold for readmission[38]. The readmission rate following birth is much lower than with medical and surgical patients[39]. This is likely to be because obstetric patients are healthier and younger than other patient populations and because the index

For peer review only - http://bmjopen.bmj.com/site/about/guidelines.xhtml

case is often undergoing a normal physiological process rather than management of a disease or condition[5]. The readmission median length of stay was 22.5 hours, which represents a sizeable provision within services each year when multiplied by the rate of readmission.

We found wide variation in the number of individuals cared for by each midwife, from 2.3 per midwife in one organisation to 4.1 per midwife in another. It is important to remember that this figure spans the inpatient maternity service where some women will receive one-to-one care in labour[40] and others will be on wards including neonates with fewer staff per person than in the birthing environment. One of the Trusts had a smaller service than the others, while also having higher rates of staffing and lower readmission rates. Smaller services require more staff to ensure services are available and to manage unscheduled demand[41, 42]. Smaller services may also be treating fewer complex cases which could account for lower readmission rates[38]. These variations were controlled for in our hierarchical analysis and also in the case-mix adjustment, so we can be confident our analysis has taken account of these factors.

Risk factors associated with maternal postpartum readmission include age, ethnicity, parity, body mass index and mode of birth[10, 15]. Individual characteristics were noted to account for most of the inter-hospital variance in postpartum readmission rates in the study by Clapp[43] and we saw further evidence of this as age and mode of birth were statistically significant predictors of readmission in all of our analyses. Staffing is a modifiable factor that has also been associated with rates of readmission[27, 28]. Having more people to care for can affect the quality of care, as some aspects can be missed or incomplete as staff prioritise and ration their time[44]. Some activities such as medicines administration are commonly prioritised, whereas patient teaching and discharge preparation may be seen as less urgent, and therefore more likely to be incomplete or missed[37]. This has been found in a number of studies including those in Malaysia[45], Australia[46], USA[37] and South Korea[47], although not all of these studies are in maternity populations.

The mechanisms for an association between staffing and readmissions need further study. Weiss et al[48] found a sequential pathway from high registered nurse staffing, to improved discharge teaching quality and readiness, to decreased odds of readmission for medical-surgical patients. However, this type of study has not been replicated in the maternity population as far as we are aware. Proposed mechanisms include reduced staff time for recognising deterioration[49], time for education[37], coordination of care[24], hand hygiene compliance[4, 50], discharge planning[44] and medicines optimisation[17]. We found that infection or sepsis was listed as the primary diagnosis in

For peer review only - http://bmjopen.bmj.com/site/about/guidelines.xhtml

23% of the postpartum readmissions. Although this was the most common identifiable reason for readmission it was not as high as that reported in the literature, where infection has been implicated in 62% of all postpartum admissions[10] and 82% of readmissions following caesarean birth[16]. Nevertheless, this is still a sizeable figure so mediating pathways from staffing linked to infection and then readmission may be worthy of exploring.

We found a statistically significant association between midwifery staffing below the mean level and higher rates of postpartum readmissions within 7 days of discharge. We went on to look at staffing levels in categories below andabove the mean levels. This categorical analysis of midwifery staffing found fewer readmissions when services were staffed above mean levels and more readmissions when staffed below mean levels, although these were not statistically significant. Service planners should consider setting staffing above mean levels if these findings are confirmed, as it is possible that mean levels in themselves represent suboptimal understaffing.

There was no clear relationship between maternity assistant staffing levels and readmission rates in our study. It may be that maternity assistants perform fewer sensitive activities which could be related to the risk of readmission, although there is very little published evidence on assistants in maternity services to compare our findings to.

Strengths and Limitations

There is limited evidence to determine optimal staffing levels in maternity services. Our research adds to the body of evidence by highlighting the association between staffing levels of midwives and rates of postpartum readmissions. Our analysis has taken account of case mix and between-hospital differences when estimating effects, but unfortunately, we were unable to adjust for other important factors such as ethnicity, parity, deprivation and body mass index from the available data. A strength of our approach is the longitudinal and granular design, so we have been able to measure the individual's exposure to staffing per shift, rather than estimating via monthly or annual data. Our approach has an advantage over sequential panel studies as the longitudinal design allows direct individual-level linking of staffing exposure to readmission despite the time lag between these variables.

For peer review only

The amount of missing data is minimal, and therefore we believe this does not materially affect the conclusions drawn. A limitation of our study is that we have been unable to extract information on the staffing close to each woman at the ward level, as two of the Trusts only had staffing data available as a whole service. We measured staffing in the first 24 hours of the admission including birth and will not have captured the hours before discharge for all women. Some of the protective mechanism of adequate staffing could be related to the period prior to discharge, with the handover of care and provision of discharge information. Also we have been unable to account for post-discharge care in the community, which is likely to have a bearing on readmission rates if women have differing opportunities to seek early intervention elsewhere[15]. A further limitation is that our study did not include the rosters of medical staff due to differences in roster configurations. If this staff group is included in future models this may modify the estimates seen for midwives and maternity assistants.

Areas for future research

Any measurable reduction in hospital readmission rates would offer significant financial benefits to hospitals[5]. The cost of increasing staffing could be offset by the potential savings in the future. Cost-effectiveness studies are needed to investigate the consequences of different staffing configurations in relation to the quality of care and related costs and savings. In future research, the components of services that help to reduce readmissions could be explored so resources are applied in areas known to be effective. This may mean targeting increased attention towards at-risk populations that account for a high proportion of readmissions[10, 18]. The provision of additional staff alone may not be fully effective in reducing readmissions unless they are focusing activity on areas of need or critical activities. A more thorough investigation of reasons for readmission would need to be included in this future work.

Conclusion

Low midwifery staffing in maternity units is associated with increased rates of readmissions within seven days after discharge but there was no adverse effect from lower assistant staffing. This is an important finding especially as readmission rates have increased in some countries in recent years, they are costly to services and distressing for families. Limiting the number of individuals cared for per midwife may have benefits beyond the inpatient stay and result in cost savings if readmissions can be reduced.

For peer review only

Contributor statement

Funding acquisition : PG, CDO, JB, DC, JJ, PM, CS.

CS and PM cleaned and linked data from different sources

LT, PG, CDO, JB, DC, JJ, PM, and CS developed the methodology.

LT conducted the investigation and analysis and wrote the original draft.

LT, PG, CDO, JB, DC, PM, CS and EKR reviewed the results and contributed to interpretation.

PG, EKR and JB supervised this project.

DC provided statistical advice.

All authors reviewed the manuscript.

Acknowledgements.

The views expressed are those of the author(s) and not necessarily those of the National Institute for Health Research, the Department of Health and Social Care, 'arms-length' bodies or other government departments.

Frankie Lambert (FL) made contributions to the work that fall short of ICMJE authorship criteria but we wish her contribution to be recognised.

Please note that, for the purpose of open access, the author has applied a Creative Commons attribution license (CC BY) to any Author Accepted Manuscript version arising from this submission.

Competing interest

No authors have declared any conflicts of interest

Funding

National Institute for Health Research Health Services & Delivery Research programme, award 128056

NIHR Applied Research Collaboration Wessex, no award number.

Data sharing statement

Due to the sensitive nature of the data and data sharing agreements with the providers we are unable to freely share the source data, but we guarantee its authenticity and the rigour of methods used in the analysis.

Ethics approval statement

This work has been reviewed and approved by the following ethics committees ERGO 52957, IRAS 128056.

For peer review only

References

1. World Health Organization, Global Health Observatory data repository, 2023. https://www.who.int/data/gho.

2. M.M. Feenstra, I. Nilsson, D.B. Danbjørg, Broken expectations of early motherhood: Mothers' experiences of early discharge after birth and readmission of their infants, Journal of clinical nursing 28(5-6) (2019) 870-881.

3. J.R. Vest, L.D. Gamm, B.A. Oxford, M.I. Gonzalez, K.M. Slawson, Determinants of preventable readmissions in the United States: a systematic review, Implementation science 5(1) (2010) 1-28.

4. K.S. Blume, K. Dietermann, U. Kirchner-Heklau, V. Winter, S. Fleischer, L.M. Kreidl, G. Meyer, J. Schreyögg, Staffing levels and nursing-sensitive patient outcomes: Umbrella review and qualitative study, Health Services Research 00 (2021) 1-23.

5. T.L. Brown, Nurse Staffing, Satisfaction and Hospital Readmission Rates in Skilled Nursing Facilities: A Quantitative, Correlational Study. Doctoral Thesis., University of Phoenix, 2020.

6. M.A. Clapp, S.E. Little, J. Zheng, A.J. Kaimal, J.N. Robinson, Hospital-level variation in postpartum readmissions, JAMA 317(20) (2017) 2128-2129.

7. L. Ellberg, B. Lundman, M. Persson, U. Hogberg, Comparison of health care utilization of postnatal programs in Sweden, Journal of Obstetric, Gynecologic, & Neonatal Nursing 34(1) (2005) 55-62.

8. National Maternity and Perinatal Audit, Clinical Report 2022. Based on births in NHS maternity services in England and Wales between 1 April 2018 and 31 March 2019, 2022. https://maternityaudit.org.uk/FilesUploaded/Ref%20336%20NMPA%20Clinical%20Report_2022.pdf .

9. M.A. Clapp, S.E. Little, J. Zheng, J.N. Robinson, A multi-state analysis of postpartum readmissions in the United States, American journal of obstetrics and gynecology 215(1) (2016) 113. e1.

10. M. Sharvit, T. Rubinstein, D. Ravid, G. Shechter-Maor, A. Fishman, T. Biron-Shental, Patients with high-risk pregnancies and complicated deliveries have an increased risk of maternal postpartum readmissions, Archives of gynecology and obstetrics 290 (2014) 629-633.

11. S. Liu, M. Heaman, M.S. Kramer, K. Demissie, S.W. Wen, S. Marcoux, M.H.S.G.o.t.C.P.S. System, Length of hospital stay, obstetric conditions at childbirth, and maternal readmission: a population-based cohort study, American journal of obstetrics and gynecology 187(3) (2002) 681-687.

12. T. Wen, V.X. Yu, J.D. Wright, D. Goffman, F. Attenello, W.J. Mack, M. D'Alton, A.M. Friedman, Postpartum length of stay and risk for readmission among women with preeclampsia, The Journal of Maternal-Fetal & Neonatal Medicine 33(7) (2020) 1086-1094.

13. J.B. Ford, C.S. Algert, J.M. Morris, C.L. Roberts, Decreasing length of maternal hospital stay is not associated with increased readmission rates, Australian and New Zealand Journal of Public Health 5(36) (2012) 430-434.

14. S. Pervez, N. Sharawi, L. Blake, A. Habib, K. Brookfield, B. Carvalho, Impact of enhanced recovery after caesarean delivery on maternal outcomes: A systematic review and meta-analysis, Anaesthesia, Critical Care & Pain Medicine (100935) (2021) 1-11.

For peer review only - http://bmjopen.bmj.com/site/about/guidelines.xhtml

15. M. Szafrańska, C. Begley, M. Carroll, D. Daly, Factors associated with maternal readmission to hospital, attendance at emergency rooms or visits to general practitioners within three months postpartum, European Journal of Obstetrics & Gynecology and Reproductive Biology 254 (2020) 251-258.

16. S.J. Kipkore, Risk Factors, Indications and Outcomes of Post Cesarean Section Readmission at Kenyatta N. Hospital: a Case-control Study. Masters Thesis., University of Nairobi, 2022.

17. C.A. Combs, D. Goffman, C.M. Pettker, Society for Maternal-Fetal Medicine special statement: a critique of postpartum readmission rate as a quality metric, American Journal of Obstetrics and Gynecology 226(4) (2022) B2-B9.

18. T. Wen, E.E. Overton, J.-J. Sheen, F.J. Attenello, W.J. Mack, M.E. D'Alton, A.M. Friedman, Risk for postpartum readmissions and associated complications based on maternal age, The Journal of Maternal-Fetal & Neonatal Medicine 34(9) (2019) 1375-1381.

19. United Nations Population Fund, The State of The World's Midwifery, 2021. https://www.unfpa.org/sites/default/files/resource-pdf/21-038-UNFPA-SoWMY2021-FastFacts-ENv4302.pdf.

20. J. Needleman, Liu, L., Shang, J., Larson, E., Association of registered nurse and nursing support staffing with inpatient hospital mortality, BMJ quality & safety 29 (2020) 10-18.

21. R.L. Kane, T.A. Shamliyan, C. Mueller, S. Duval, T.J. Wilt, The association of registered nurse staffing levels and patient outcomes: systematic review and meta-analysis, Medical care (2007) 1195-1204.

22. L.H. Aiken, D.M. Sloane, J. Ball, L. Bruyneel, A.M. Rafferty, P. Griffiths, Patient satisfaction with hospital care and nurses in England: an observational study, BMJ Open 8(1) (2018) e019189.

23. C. Ma, M.D. McHugh, L.H. Aiken, Organization of hospital nursing and 30-day readmissions in Medicare patients undergoing surgery, Medical Care 53(1) (2015) 65-70.

24. M.D. McHugh, J. Berez, D.S. Small, Hospitals with higher nurse staffing had lower odds of readmissions penalties than hospitals with lower staffing, Health Affairs 32(10) (2013) 1740-1747.

25. K.B. Lasater, M. McHugh, P.R. Rosenbaum, L.H. Aiken, H. Smith, J.G. Reiter, B.A. Niknam, A.S. Hill, L.L. Hochman, S. Jain, J.H. Silber, Valuing hospital investments in nursing: multistate matched-cohort study of surgical patients, BMJ Quality & Safety 30(1) (2021) 46-55.

26. L. Turner, P. Griffiths, E. Kitson-Reynolds, Midwifery and nurse staffing of inpatient maternity services – a systematic scoping review of associations with outcomes and quality of care, Midwifery 103 (2021) 103118.

27. V. Gerova, P. Griffiths, S. Jones, D. Bick, The association between midwifery staffing and outcomes in maternity units in England: observational study using routinely collected data, Kings College London, 2010.

28. S.J. Kim, K.T. Han, H.J. Lee, J.A. Kwon, E.C. Park, Positive effects of medical staffing on readmission within 30 days after discharge: a retrospective analysis of obstetrics and gynecology data, European Journal of Public Health 26(6) (2016) 935-939.

For peer review only - http://bmjopen.bmj.com/site/about/guidelines.xhtml

29. P. Griffiths, Dall'Ora, C., Ball, J., Culliford, D., Emmanuel, T., Jones, J., Lambert, F., Meredith, P., Rubbo, B., Schmidt, O., Saville, C. , Consequences, costs and cost-effectiveness of different workforce configurations in English acute hospitals: a longitudinal retrospective study using routinely collected data (protocol), in: A.I. NIHR128056 (Ed.) 2022.

30. NHS England, NHS data model and dictionary, 2023. https://www.datadictionary.nhs.uk/supporting_information/healthcare_resource_group.html.

31. NHS Digital, Summary Hospital-level Mortality Indicator (SHMI), 2023. https://digital.nhs.uk/data-and-information/publications/ci-hub/summary-hospital-level-mortality-indicator-shmi.

32. NHS Digital, Indicator Supporting Documentation. IAP00385 Summary Hospital-level Mortality Indicator (SHMI), 2019. https://www.nice.org.uk/Media/Default/Standards-and-indicators/National-Library-of-Quality-Indicators/IAP00385%20supporting%20documentation.docx.

33. NHS Improvement, Care hours per patient day (CHPPD): guidance for acute and acute specialist trusts, 2021.

34. D.L. Chin, H. Bang, R.N. Manickam, P.S. Romano, Rethinking thirty-day hospital readmissions: shorter intervals might be better indicators of quality of care, Health Affairs 35(10) (2016) 1867-1875.

35. K.L. Graham, E.H. Wilker, M.D. Howell, R.B. Davis, E.R. Marcantonio, Differences between early and late readmissions among patients: a cohort study, Annals of Internal Medicine 162(11) (2015) 741-749.

36. N.I. Goldfield, E.C. McCullough, J.S. Hughes, A.M. Tang, B. Eastman, L.K. Rawlins, R.F. Averill, Identifying potentially preventable readmissions, Health care financing review 30(1) (2008) 75.

37. H.L. Tubbs-Cooley, J.P. Cimiotti, J.H. Silber, D.M. Sloane, L.H. Aiken, An observational study of nurse staffing ratios and hospital readmission among children admitted for common conditions, BMJ Quality & Safety 22(9) (2013) 735-742.

38. C. Fischer, H.F. Lingsma, P.J. Marang-van de Mheen, D.S. Kringos, N.S. Klazinga, E.W. Steyerberg, Is the readmission rate a valid quality indicator? A review of the evidence, PloS one 9(11) (2014) e112282.

39. NHS Digital, Emergency readmissions to hospital within 30 days of discharge by diagnosis, 2022. https://digital.nhs.uk/data-and-information/publications/statistical/compendium-emergency-readmissions/current/emergency-readmissions-to-hospital-within-30-days-of-discharge.

40. Care Quality Commission, Maternity survey 2021, 2022.

41. L. Vaughan, N. Edwards, The problems of smaller, rural and remote hospitals: separating facts from fiction, Future Healthcare Journal 7(1) (2020) 38.

42. N. Edwards, Vaughan, L., Palmer, B., Maternity services in smaller hospitals: a call to action, 2020. https://www.nuffieldtrust.org.uk/research/maternity-services-in-smaller-hospitals-a-call-to-action.

43. M.A. Clapp, S.E. Little, J. Zheng, J.N. Robinson, A.J. Kaimal, The relative effects of patient and hospital factors on postpartum readmissions, Journal of Perinatology 38(7) (2018) 804-812.

For peer review only - http://bmjopen.bmj.com/site/about/guidelines.xhtml

44. K.R. Simpson, A. Lyndon, C. Ruhl, Consequences of inadequate staffing include missed care, potential failure to rescue, and job stress and dissatisfaction, Journal of Obstetric, Gynecologic & Neonatal Nursing 45(4) (2016) 481-490.

45. S.T. Nahasaram, V. Ramoo, W.L. Lee, Missed nursing care in the Malaysian context: A cross-sectional study from nurses' perspective, Journal of nursing management 00 (2021) 1-9.

46. R.A. Albsoul, Investigating missed nursing care in an Australian acute care hospital: An exploratory study., Queensland University of Technology, 2019.

47. E. Lee, B.J. Kalisch, Identification and comparison of missed nursing care in the United States and South Korea, Journal of Clinical Nursing 00 (2021) 1-11.

48. M.E. Weiss, O. Yakusheva, K.L. Bobay, Quality and cost analysis of nurse staffing, discharge preparation, and postdischarge utilization, Health services research 46(5) (2011) 1473-1494.

49. L. Jeffs, I. Dhalla, R. Cardoso, C.M. Bell, The perspectives of patients, family members and healthcare professionals on readmissions: preventable or inevitable?, Journal of interprofessional care 28(6) (2014) 507-512.

50. P.J. Rae, S. Pearce, P.J. Greaves, C. Dall'Ora, P. Griffiths, R. Endacott, Outcomes sensitive to critical care nurse staffing levels: A systematic review, Intensive and Critical Care Nursing 67 (2021) 103110.

For peer review only - http://bmjopen.bmj.com/site/about/guidelines.xhtml

SUPPLEMENTARY INFORMATION

Table of contents

S1 Model fit and missing data 2
1.1 Model fit by Akaike information criterion (AIC) and Bayesian information criterion (BIC) .. 2
1.2 Collinearity testing 2
1.3 Missing data per person in the study population (women who had a birth admission) 2
S2 Diagnosis codes for the 2903 postpartum readmissions 3
S3 Staffing levels expressed as Individuals per Registered Midwife (RM) and Maternity Assistant (MA) 5
S4 Staffing on day and night shifts over time in each of the Trusts 6
S5 Skill mix of staff within the three Trusts 7
S6 Trends in staffing over time in each of the Trusts 8
S7 Mean admissions per day and night in each Trust 9
S8 Univariable analyses 10
S8.1 Age category 10
S8.2 Mode of birth 11
S8.3 SHMI risk 12
S8.4 Understaffing by Registered Midwives, Maternity Assistants and Overall staffing 12
S8.5 Turnover (admissions + discharges) more than expected (mean) value for Trust 12
S8.6 Skill mix lower than expected (mean) levels for service 13
S9 Full models for staffing below the mean compared to mean staffing (HPPD) 14
S10. Full models with staffing expressed as individuals per midwife/maternity assistant (absolute level) 15
For outcome of readmission within 7 days 15
S11 Additional analysis : High and low staffing categories compared with mean staffing 16
S12 Frequency of exposure to staffing categories in study population 17
S13 Flow chart for data selection 18

For peer review only
For peer review only - http://bmjopen.bmj.com/site/about/guidelines.xhtml

SUPPLEMENTARY INFORMATION

S1 Model fit and missing data

1.1 Model fit by Akaike information criterion (AIC) and Bayesian information criterion (BIC)

Postpartum readmission	7 days from discharge model		30 days from discharge model	
	AIC	BIC	AIC	BIC
Empty model nested in Trust	18036.62	18054.76	23539.27	23557.41
Age category added	18031.47	18058.68	23527.22	23554.43
Mode of birth added	17667.94	17703.96	23024.88	23060.91
RM<mean staffing added	17240.87	17285.81	22518.88	22563.81
MA<mean staffing added	17112.23	17166.03	22296.37	22350.17
SHMI added	17008.74	17071.47	22149.12	22211.84
Skill mix added (did not improve model fit so excluded from full model)	17010.53	17082.21	22148.77	22220.45
Turnover added (did not improve model fit so excluded from full model)	17012	17092.65	22149.99	22230.63

RM Registered Midwife, MA Maternity Assistant, SHMI Standardised Hospital Mortality Indicator

1.2 Collinearity testing

Test for collinearity using the Variance inflation factor (VIF)

	VIF
understaffRMless1	1.37
understaffMAless1	1.90
age_category	1.00
Mode_birth	1.00
SHMI_risk	1.00
Higher expected turnover	1.02
Less expected skill mix	1.56

RM Registered Midwife, MA Maternity Assistant

VIF scores are low (<2) so collinearity was not detected. A VIF indicating high collinearity would be >10.

1.3 Missing data per person in the study population (women who had a birth admission)

Age	None
Length of stay	None
Mode birth	None
SHMI	385/64,250 (0.6%)
Staffing Registered midwives	1,258/64,250 (1.96%)
Staffing Maternity Assistants	2,452/64,250 (3.82%)

(Maternity assistant staffing data was missing from Trust A after 28.1.2019)

For peer review only - http://bmjopen.bmj.com/site/about/guidelines.xhtml

SUPPLEMENTARY INFORMATION

S2 Diagnosis codes for the 2903 postpartum readmissions

**ICD codes for primary diagnosis matched to description** Where categories are broad the second diagnosis code has been reported at the end of this table	Count	percentage
Total number of readmissions	**2903**	
Other complications of the puerperium, not elsewhere classified*	482	16.6%
Care and examination of lactating mother**	373	12.8%
Delayed and secondary postpartum haemorrhage	310	10.7%
Puerperal sepsis	246	8.5%
Infection of obstetric surgical wound	227	7.8%
Other specified diseases and conditions complicating pregnancy, childbirth and the puerperium***	159	5.5%
Disruption of caesarean section wound	104	3.6%
Disruption of perineal obstetric wound	103	3.5%
Maternal care for rhesus isoimmunization	71	2.4%
Nonpurulent mastitis associated with childbirth	63	2.2%
Unspecified maternal hypertension	57	2.0%
Haematoma of obstetric wound	45	1.6%
Urinary tract infection following delivery	43	1.5%
Persons encountering health services in other specified circumstances	40	1.4%
Pyrexia of unknown origin following delivery	39	1.3%
Fitting and adjustment of urinary device	33	1.1%
Anaemia complicating pregnancy, childbirth and the puerperium	30	1.0%
Diseases of the digestive system complicating pregnancy, childbirth and the puerperium	26	0.9%
Care and examination immediately after delivery	25	0.9%
Spinal and epidural anaesthesia-induced headache during the puerperium	24	0.8%
Other prophylactic chemotherapy	23	0.8%
Spinal and epidural anaesthesia-induced headache during labour and delivery	21	0.7%
Gestational [pregnancy-induced] hypertension	20	0.7%
Other infection of genital tract following delivery	17	0.6%
Prophylactic immunotherapy	16	0.6%
Other specified pregnancy-related conditions	15	0.5%
Other maternal infectious and parasitic diseases complicating pregnancy, childbirth and the puerperium	14	0.5%
Pre-eclampsia, unspecified	14	0.5%
Retained portions of placenta and membranes, without haemorrhage	13	0.4%
Routine postpartum follow-up	11	0.4%
Supervision of other normal pregnancy	11	0.4%
Other specified puerperal infections	9	0.3%
Diseases of the respiratory system complicating pregnancy, childbirth and the puerperium	8	0.3%
Unspecified infection of urinary tract in pregnancy	8	0.3%
Complication of the puerperium, unspecified	7	0.2%
Diseases of the circulatory system complicating pregnancy, childbirth and the puerperium	7	0.2%
Obstetric blood-clot embolism	7	0.2%
Attention to surgical dressings and sutures	6	0.2%
Elevated blood-pressure reading, without diagnosis of hypertension	6	0.2%
Examination and observation for other specified reasons	6	0.2%

For peer review only - http://bmjopen.bmj.com/site/about/guidelines.xhtml

SUPPLEMENTARY INFORMATION

ICD codes for primary diagnosis matched to description<br>Where categories are broad the second diagnosis code has been reported at the end of this table	Count	percentage
Mental disorders and diseases of the nervous system complicating pregnancy, childbirth and the puerperium	6	0.2%
Other immediate postpartum haemorrhage	6	0.2%
Maternal care due to uterine scar from previous surgery	5	0.2%
Other and unspecified disorders of breast associated with childbirth	5	0.2%
Postpartum coagulation defects	5	0.2%
Second degree perineal laceration during delivery	5	0.2%
Superficial thrombophlebitis in the puerperium	5	0.2%
Unspecified pre-existing hypertension complicating pregnancy, childbirth and the puerperium	5	0.2%
Other conditions with ICD codes affecting <5 women in dataset each (condensed for reporting in this table)	115	4.0%
Blank	7	0.2%

* 68/478 had blank second diagnosis code, 179/478 had ICD indicating a type of pain as second diagnosis code
** 226/373 had blank second diagnosis code, 125/373 had 'Healthy person accompanying sick person' as second diagnosis code
***24/159 had blank second diagnosis code, no pattern seen in remaining ICD codes

When totalling the above 677/2903 (23.3%) of postpartum readmissions were due to infection or sepsis, 316/2903 (10.9%) were due to haemorrhage

Missing ICD diagnosis code for readmissions 7/2903 (0.2%)

For peer review only

SUPPLEMENTARY INFORMATION

S3 Staffing levels expressed as Individuals per Registered Midwife (RM) and Maternity Assistant (MA)

Trust	Mean RM HPPD	Individuals per Midwife	Mean MA HPPD	Individuals per Maternity Assistant
A	11.747	2.330	4.869	5.934
B	5.940	4.104	1.759	14.205
C	6.906	3.576	2.650	9.387
All Trusts (non weighted average)	6.520	3.879	2.146	12.67

RM Registered Midwife, MA Maternity Assistant, HPPD= Hours Per Patient Day

Staffing was calculated for each Trust in the study period and averaged per day.
The 'individuals cared for per staff member' was calculated using the following formula = 24/HPPD.

For peer review only

For peer review only - http://bmjopen.bmj.com/site/about/guidelines.xhtml

SUPPLEMENTARY INFORMATION

S4 Staffing on day and night shifts over time in each of the Trusts

Data for staffing for whole admitted population during specified time periods

Year	DAY or NIGHT	Mean RM HPPD	Mean MA HPPD	Mean Overall HPPD
Trust A				
2015	Day	10.75	4.56	15.32
2015	Night	11.10	3.66	14.77
2016	Day	11.73	5.47	17.20
2016	Night	11.76	5.18	16.94
2017	Day	14.22	6.04	20.26
2017	Night	13.54	5.46	19.00
2018	Day	14.08	5.97	20.04
2018	Night	13.81	5.09	18.90
2019	Day	14.68	5.77	19.06
2019	Night	14.28	4.84	17.96
2020	Day	12.57	Not available	Not available
2020	Night	12.44	Not available	Not available
Trust B				
2015	Day	7.00	1.82	8.83
2015	Night	5.09	1.52	6.60
2016	Day	6.93	1.86	8.80
2016	Night	4.99	1.53	6.52
2017	Day	7.19	2.08	9.27
2017	Night	5.04	1.65	6.70
2018	Day	6.71	1.81	8.52
2018	Night	4.81	1.34	6.15
2019	Day	6.92	2.15	9.07
2019	Night	4.89	1.72	6.61
2020	Day	6.61	2.35	8.96
2020	Night	4.84	2.09	6.93
Trust C				
2015	Day	7.05	1.98	9.03
2015	Night	5.82	2.01	7.83
2016	Day	7.02	2.17	9.19
2016	Night	5.91	2.12	8.03
2017	Day	7.23	2.52	9.75
2017	Night	6.06	2.37	8.44
2018	Day	7.34	2.76	10.10
2018	Night	6.16	2.64	8.80
2019	Day	7.91	2.84	10.76
2019	Night	6.96	2.85	9.81
2020	Day	8.62	3.25	11.87
2020	Night	7.84	3.10	10.94

RM = registered midwife MA=maternity assistant HPPD= Hours Per Patient Day

For peer review only - http://bmjopen.bmj.com/site/about/guidelines.xhtml

SUPPLEMENTARY INFORMATION

S5 Skill mix of staff within the three Trusts

This table indicates the skill mix and seniority of staff within Registered Midwife and Maternity Assistant groups.

	Sum of worked hours and % of total in each staff group					
	Total sum of worked hrs	RM Band 6 or above	Registered midwives	Registered nurses	Band 3 care assistants	Band 2 care assistants
Trust A*	316,880	33.5%	70.7%	0.09%	2.2%	26.8%
Trust B	1,990,058	60.4%	77.4%	0%	3.5%	19.1%
Trust C	933,846	59.8%	73.4% (nurses not listed separately)		7.3%	19.2%

*up to 28.1.2019 as care assistant unavailable after this date

The bands refer to NHS Employers Agenda for Change pay scales.
Band 3 is a senior maternity assistant, Band 6 is a senior registered midwife.

For peer review only - http://bmjopen.bmj.com/site/about/guidelines.xhtml

SUPPLEMENTARY INFORMATION

S6 Trends in staffing over time in each of the Trusts

RM = registered midwife MA=maternity assistant HPPD= Hours Per Patient Day

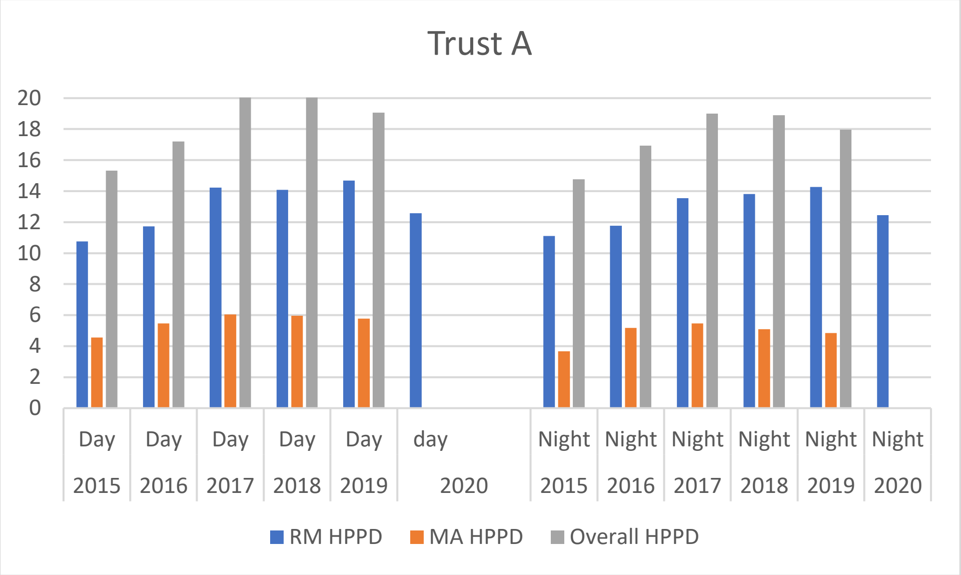

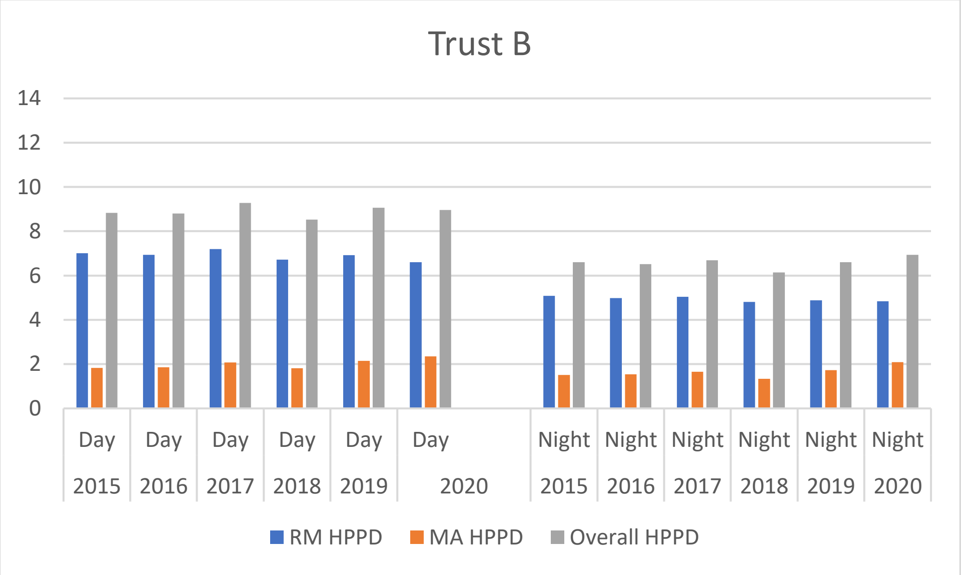

SUPPLEMENTARY INFORMATION

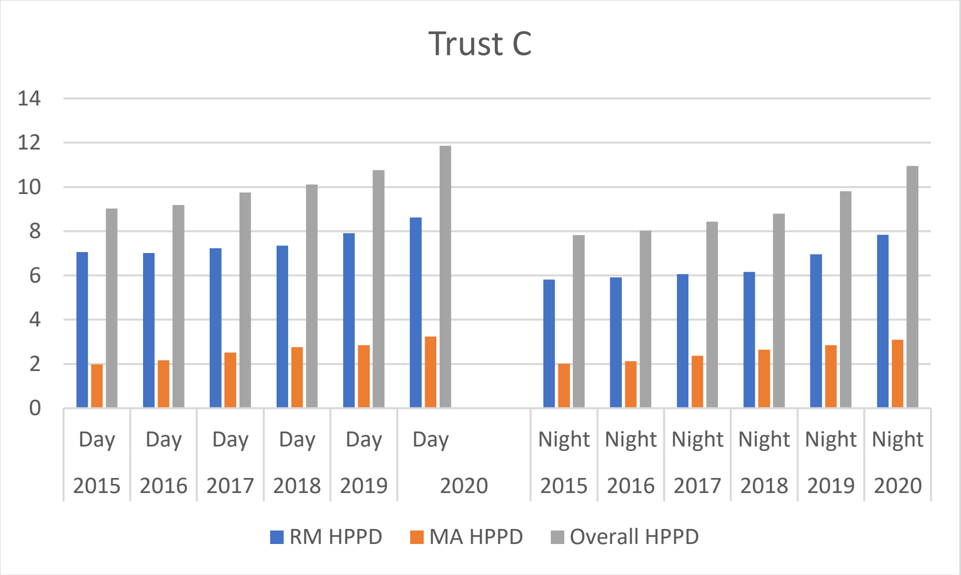

S7 Mean admissions per day and night in each Trust

	Mean admissions per Day	Mean admissions per Night
Trust A	3.2	1.7
Trust B	39.0	28.4
Trust C	15.6	13.5

For peer review only - http://bmjopen.bmj.com/site/about/guidelines.xhtml

SUPPLEMENTARY INFORMATION

S8 Univariable analyses

Based on dataset with 64,250 records. Cases leading to birth only and their exposure to staffing and outcomes. Each independent variable was entered into a regression model with only this variable and the readmission outcome variable, nested within hospital Trust.

S8.1 Age category

For outcome of readmission within 7 days
(other age categories are compared with the 20-24 age group)

	Readmitted within 7 days		
	OR	95% CI	
Age category			
<20 years	1.009	0.770	1.323
20-24 years	1.000	reference category	
25-29 years	0.954	0.828	1.100
30-34 years	1.040	0.906	1.195
35-39 years	1.043	0.893	1.219
40+ years	1.476	1.193	1.827

For outcome of readmission within 30 days
(other age categories are compared with the 20-24 age group)

	Readmitted within 30 days		
	OR	95% CI	
Age category			
<20 years	0.994	0.787	1.256
20-24 years	1.000	reference category	
25-29 years	1.013	0.897	1.143
30-34 years	1.095	0.973	1.233
35-39 years	1.125	0.986	1.284
40+ years	1.454	1.208	1.750

For peer review only

SUPPLEMENTARY INFORMATION

S8.2 Mode of birth

For outcome of readmission within 7 days
(other birth categories are compared with spontaneous vaginal birth)

	Readmitted within 7 days		
	OR	95% CI	
Assisted birth	1.838	1.622	2.083
Emergency Caesarean birth	1.888	1.685	2.116
Spontaneous vaginal birth	1.000	reference category	
Planned Caesarean birth	1.500	1.310	1.717

For outcome of readmission within 30 days
(other birth categories are compared with spontaneous vaginal birth)

	Readmitted within 30 days		
	OR	95% CI	
Assisted birth	1.828	1.642	2.036
Emergency Caesarean birth	2.041	1.854	2.247
Spontaneous vaginal birth		reference category	
Planned Caesarean birth	1.706	1.526	1.908

For peer review only

For peer review only - http://bmjopen.bmj.com/site/about/guidelines.xhtml

SUPPLEMENTARY INFORMATION

S8.3 SHMI risk

For outcome of readmission within 7 days

	OR	95% CI	
SHMI risk	1.554	0.601	4.021

For outcome of readmission within 30 days

	OR	95% CI	
SHMI risk	1.546	0.671	3.560

S8.4 Understaffing by Registered Midwives, Maternity Assistants and Overall staffing

For outcome of readmission within 7 days

	Readmitted within 7 days		
	OR	95% CI	
Registered Midwife staffing <mean	1.065	0.974	1.166
Maternity Assistant staffing <mean	0.984	0.898	1.077
Overall staffing <mean	1.054	0.964	1.154

For outcome of readmission within 30 days

	Readmitted within 30 days		
	OR	95% CI	
Registered Midwife staffing <mean	1.041	0.965	1.123
Maternity Assistant staffing <mean	0.980	0.908	1.059
Overall staffing <mean	1.041	0.964	1.123

S8.5 Turnover (admissions + discharges) more than expected (mean) value for Trust

For outcome of readmission within 7 days

	Readmitted within 7 days		
	OR	95% CI	
Turnover > mean	0.972	0.889	1.062

For outcome of readmission within 30 days

	Readmitted within 30 days		
	OR	95% CI	
Turnover > mean	0.966	0.896	1.042

For peer review only

For peer review only - http://bmjopen.bmj.com/site/about/guidelines.xhtml

SUPPLEMENTARY INFORMATION

S8.6 Skill mix lower than expected (mean) levels for service

For outcome of readmission within 7 days

	Readmitted within 7 days	
	OR	95% CI
Skill mix <mean	1.017	0.928 1.113

For outcome of readmission within 30 days

	Readmitted within 30 days	
	OR	95% CI
Skill mix <mean	0.979	0.907 1.057

For peer review only

For peer review only - http://bmjopen.bmj.com/site/about/guidelines.xhtml

SUPPLEMENTARY INFORMATION

S9 Full models for staffing below the mean compared to mean staffing (HPPD)

For outcome of readmission within 7 days

Variable	OR readmission 7 days	95% CI	
<20 years	1.066	0.810	1.402
20-24 years	1.000	reference category	
25-29 years	0.914	0.790	1.057
30-34 years	0.983	0.853	1.133
35-39 years	0.983	0.837	1.153
40+ years	1.368	1.099	1.703
Assisted birth	1.873	1.648	2.128
Emergency Caesarean birth	1.912	1.701	2.148
Spontaneous vaginal birth	1.000	reference category	
Planned Caesarean birth	1.518	1.321	1.745
Exposed to staffing below mean Registered midwives (HPPD)	1.108	1.003	1.223
Exposed to staffing below mean Maternity assistants (HPPD)	0.957	0.866	1.057
SHMI risk	0.687	0.108	4.360

For outcome of readmission within 30 days

Variable	OR readmission 30 days	95% CI	
<20 years	1.065	0.840	1.350
20-24 years	1.000	reference category	
25-29 years	0.969	0.856	1.098
30-34 years	1.024	0.906	1.156
35-39 years	1.046	0.913	1.198
40+ years	1.318	1.089	1.594
Assisted birth	1.850	1.657	2.065
Emergency Caesarean birth	2.077	1.883	2.291
Spontaneous vaginal birth	1.000	reference category	
Planned Caesarean birth	1.707	1.522	1.915
Exposed to staffing below mean Registered midwives (HPPD)	1.080	0.994	1.174
Exposed to staffing below mean Maternity assistants (HPPD)	0.965	0.887	1.049
SHMI risk	0.8868	0.2421	3.2480

For peer review only

SUPPLEMENTARY INFORMATION

S10. Full models with staffing expressed as individuals per midwife/maternity assistant (absolute level)

For outcome of readmission within 7 days

Variable	OR readmission 7 days	95% CI
<20 years	1.066	0.811 1.402
20-24 years	1.000	reference category
25-29 years	0.913	0.789 1.056
30-34 years	0.983	0.853 1.133
35-39 years	0.983	0.838 1.154
40+ years	1.368	1.099 1.702
Assisted birth	1.873	1.648 2.128
Emergency Caesarean birth	1.912	1.721 2.148
Spontaneous vaginal birth	1.000	reference category
Planned Caesarean birth	1.519	1.322 1.745
Number of individuals to care for per midwife	1.063	0.960 1.177
Number of individuals to care for per maternity assistant	0.998	0.978 1.018
SHMI risk	0.691	0.109 4.370

For outcome of readmission within 30 days

Variable	OR readmission 30 days	95% CI
<20 years	1.066	0.840 1.351
20-24 years	1.000	reference category
25-29 years	0.969	0.856 1.098
30-34 years	1.025	0.907 1.157
35-39 years	1.046	0.913 1.199
40+ years	1.317	1.088 1.593
Assisted birth	1.850	1.657 2.064
Emergency Caesarean birth	2.077	1.883 2.291
Spontaneous vaginal birth	1.000	reference category
Planned Caesarean birth	1.706	1.520 1.913
Number of individuals to care for per midwife	1.013	0.929 1.105
Number of individuals to care for per maternity assistant	1.002	0.986 1.019
SHMI risk	0.889	0.243 3.255

For peer review only - http://bmjopen.bmj.com/site/about/guidelines.xhtml

SUPPLEMENTARY INFORMATION

S11 Additional analysis : High and low staffing categories compared with mean staffing

Observed (O) and Expected (E) staffing levels were calculated,
with E being the mean for each service.
The ratio of O/E was derived from these figures
The reference category was defined as O/E between 0.95 and 1.05
Low staffing was defined as O/E <0.95
High staffing was defined as O/E >1.05

This grouping allowed the mean (plus 5% either side of the mean) to be considered as an 'expected' level of staffing, allowing for a small amount of variability. The criteria for 'low' and 'high' were set outside this expected level.

This grouping was considered to be more meaningful that splitting the data into tertiles or quantiles or using standard deviations, as it is more reflective of a real clinical scenario and will provide a better insight to service planners.

For outcome of readmission within 7 days

Variable	OR readmission 7 days	95% CI	
<20 years	1.068	0.812	1.404
20-24 years	1.000	reference category	
25-29 years	0.914	0.790	1.057
30-34 years	0.984	0.854	1.134
35-39 years	0.984	0.838	1.154
40+ years	1.370	1.101	1.706
Assisted birth	1.873	1.648	2.127
Emergency Caesarean birth	1.911	1.701	2.147
Spontaneous vaginal birth	1.000	reference category	
Planned Caesarean birth	1.518	1.321	1.744
Midwifery staffing			
Low, 95% of mean or less	1.056	0.943	1.182
Near to mean 95%-105%	1.000	reference category	
High 105% of mean or more	0.973	0.862	1.098
Maternity assistant staffing			
Low, 95% of mean or less	0.924	0.816	1.046
Near to mean 95%-105%	1.000	reference category	
High 105% of mean or more	0.935	0.825	1.059
SHMI risk	0.693	0.111	4.335

SHMI Standardised Hospital Mortality Indicator

For peer review only - http://bmjopen.bmj.com/site/about/guidelines.xhtml

SUPPLEMENTARY INFORMATION

For outcome of readmission within 30 days

Variable	OR readmission 7 days	95% CI	
<20 years	1.066	0.841	1.351
20-24 years	1.000	reference category	
25-29 years	0.970	0.856	1.099
30-34 years	1.025	0.908	1.158
35-39 years	1.046	0.913	1.199
40+ years	1.319	1.090	1.595
Assisted birth	1.850	1.657	2.065
Emergency Caesarean birth	2.076	1.882	2.290
Spontaneous vaginal birth	1.000	reference category	
Planned Caesarean birth	1.705	1.520	1.913
Midwifery staffing			
Low, 95% of mean or less	1.002	0.911	1.102
Near to mean 95%-105%	1.000	reference category	
High 105% of mean or more	0.957	0.865	1.060
Maternity assistant staffing			
Low, 95% of mean or less	0.968	0.871	1.076
Near to mean 95%-105%	1.000	reference category	
High 105% of mean or more	0.968	0.870	1.077
SHMI risk	0.889	0.243	3.247

SHMI Standardised Hospital Mortality Indicator

S12 Frequency of exposure to staffing categories in study population

For registered midwives the staffing categories breakdown is as follows

1,258/64,250 (1.96%) staffing values known to be missing as reported in supplement 1.3

Staffing caregory	Frequency	percent
Low, 95% of mean or less	24,954	39.61
Near to mean 95%-105%	18,576	29.49
High 105% of mean or more	19,462	30.90

.

For maternity assistants the staffing categories breakdown is as follows

2,452/64,250 (3.82%) staffing values known to be missing as reported in supplement 1.3

Staffing caregory	Frequency	percent
Low, 95% of mean or less	26,374	42.68
Near to mean 95%-105%	12,520	20.26
High 105% of mean or more	22,903	37.06

For peer review only

SUPPLEMENTARY INFORMATION

S13 Flow chart for data selection

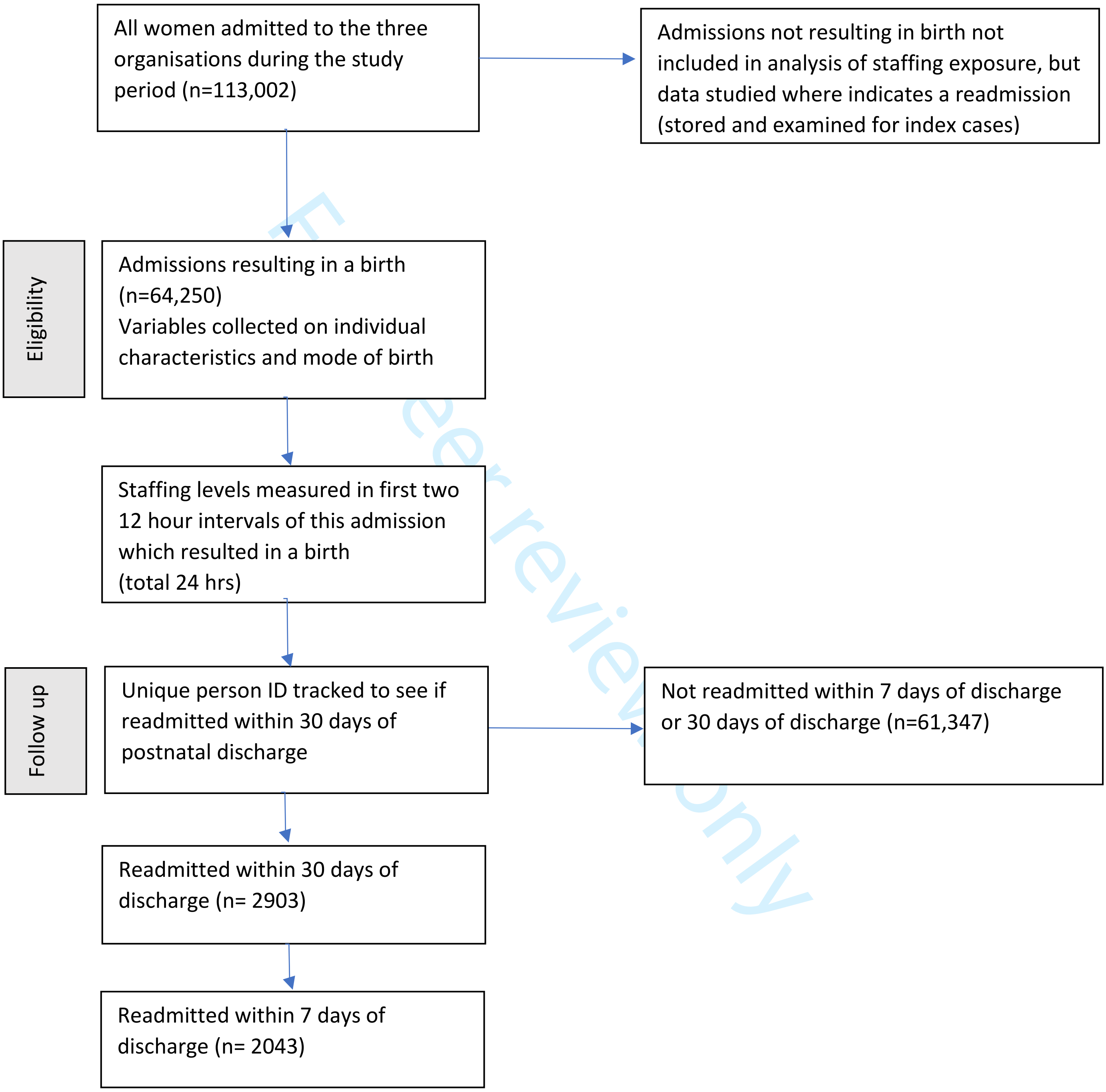

For peer review only

For peer review only - http://bmjopen.bmj.com/site/about/guidelines.xhtml

STROBE Statement—Checklist of items that should be included in reports of ***cohort studies***

	Item No	Recommendation	TRACKED CHANGES DOCUMENT Line No
**Title and abstract**	1	(*a*) Indicate the study's design with a commonly used term in the title or the abstract	1,28
		(*b*) Provide in the abstract an informative and balanced summary of what was done and what was found	30-58
**Introduction**			
Background/rationale	2	Explain the scientific background and rationale for the investigation being reported	82-117
Objectives	3	State specific objectives, including any prespecified hypotheses	118-120
**Methods**			
Study design	4	Present key elements of study design early in the paper	124-125
Setting	5	Describe the setting, locations, and relevant dates, including periods of recruitment, exposure, follow-up, and data collection	125-128
Participants	6	(*a*) Give the eligibility criteria, and the sources and methods of selection of participants. Describe methods of follow-up<br>(*b*) For matched studies, give matching criteria and number of exposed and unexposed	125-136
Variables	7	Clearly define all outcomes, exposures, predictors, potential confounders, and effect modifiers. Give diagnostic criteria, if applicable	136-186
Data sources/ measurement	8*	For each variable of interest, give sources of data and details of methods of assessment (measurement). Describe comparability of assessment methods if there is more than one group	136-186
Bias	9	Describe any efforts to address potential sources of bias	163-167, 190-192
Study size	10	Explain how the study size was arrived at	125-128, supp 13
Quantitative variables	11	Explain how quantitative variables were handled in the analyses. If applicable, describe which groupings were chosen and why	187-203, supp 11
Statistical methods	12	(*a*) Describe all statistical methods, including those used to control for confounding<br>(*b*) Describe any methods used to examine subgroups and interactions<br>(*c*) Explain how missing data were addressed<br>(*d*) If applicable, explain how loss to follow-up was addressed<br>(*e*) Describe any sensitivity analyses	187-206 supp 1, supp 10,11
**Results**			
Participants	13*	(a) Report numbers of individuals at each stage of study—eg numbers potentially eligible, examined for eligibility, confirmed eligible, included in the study, completing follow-up, and analysed<br>(b) Give reasons for non-participation at each stage<br>(c) Consider use of a flow diagram	214-216, supp 13
Descriptive data	14*	(a) Give characteristics of study participants (eg demographic, clinical, social) and information on exposures and potential confounders	Table 1, 215-222
		(b) Indicate number of participants with missing data for each variable of interest	Supplement1

For peer review only

		(c) Summarise follow-up time (eg, average and total amount)	188-190
Outcome data	15*	Report numbers of outcome events or summary measures over time	223-228

For peer review only

For peer review only - http://bmjopen.bmj.com/site/about/guidelines.xhtml

Main results	16	(*a*) Give unadjusted estimates and, if applicable, confounder-adjusted estimates and their precision (eg, 95% confidence interval). Make clear which confounders were adjusted for and why they were included	Table 3, 246-258
		(*b*) Report category boundaries when continuous variables were categorized	Tables2-4
		(*c*) If relevant, consider translating estimates of relative risk into absolute risk for a meaningful time period	277-280
Other analyses	17	Report other analyses done—eg analyses of subgroups and interactions, and sensitivity analyses	277-288, S11
**Discussion**			
Key results	18	Summarise key results with reference to study objectives	295-301
Limitations	19	Discuss limitations of the study, taking into account sources of potential bias or imprecision. Discuss both direction and magnitude of any potential bias	360-382
Interpretation	20	Give a cautious overall interpretation of results considering objectives, limitations, multiplicity of analyses, results from similar studies, and other relevant evidence	295-304
Generalisability	21	Discuss the generalisability (external validity) of the study results	305-307
**Other information**			
Funding	22	Give the source of funding and the role of the funders for the present study and, if applicable, for the original study on which the present article is based	425-427

*Give information separately for exposed and unexposed groups.

Note: An Explanation and Elaboration article discusses each checklist item and gives methodological background and published examples of transparent reporting. The STROBE checklist is best used in conjunction with this article (freely available on the Web sites of PLoS Medicine at http://www.plosmedicine.org/, Annals of Internal Medicine at http://www.annals.org/, and Epidemiology at http://www.epidem.com/). Information on the STROBE Initiative is available at http://www.strobe-statement.org.

For peer review only - http://bmjopen.bmj.com/site/about/guidelines.xhtml

BMJ Open

Inpatient midwifery staffing levels and postpartum readmissions – a retrospective multi-centre longitudinal study

Journal:	*BMJ Open*
Manuscript ID	bmjopen-2023-077710.R2
Article Type:	Original research
Date Submitted by the Author:	12-Mar-2024

Complete List of Authors:	Turner, Lesley; University of Southampton, School of Health Sciences<br>Saville, Christina; University of Southampton, School of Health Sciences<br>Ball, Jane; University of Southampton, School of Health Sciences<br>Culliford, David; NIHR Applied Research Collaboration Wessex; University of Southampton, School of Health Sciences<br>Dall'Ora, Chiara; University of Southampton, School of Health Sciences<br>Jones, Jeremy; University of Southampton, School of Health Sciences<br>Kitson-Reynolds, Ellen; University of Southampton, School of Health Sciences<br>Meredith, Paul; University of Southampton, School of Health Sciences<br>Griffiths, Peter; University of Southampton, School of Health Sciences; National Institute for Health Research Applied Research Centre (Wessex)

**Primary Subject Heading**:	Obstetrics and gynaecology
Secondary Subject Heading:	Health services research
Keywords:	OBSTETRICS, Nursing Care, Postpartum Period, Human resource management < HEALTH SERVICES ADMINISTRATION & MANAGEMENT

SCHOLARONE™
Manuscripts

I, the Submitting Author has the right to grant and does grant on behalf of all authors of the Work (as defined in the below author licence), an exclusive licence and/or a non-exclusive licence for contributions from authors who are: i) UK Crown employees; ii) where BMJ has agreed a CC-BY licence shall apply, and/or iii) in accordance with the terms applicable for US Federal Government officers or employees acting as part of their official duties; on a worldwide, perpetual, irrevocable, royalty-free basis to BMJ Publishing Group Ltd ("BMJ") its licensees and where the relevant Journal is co-owned by BMJ to the co-owners of the Journal, to publish the Work in this journal and any other BMJ products and to exploit all rights, as set out in our licence.

The Submitting Author accepts and understands that any supply made under these terms is made by BMJ to the Submitting Author unless you are acting as an employee on behalf of your employer or a postgraduate student of an affiliated institution which is paying any applicable article publishing charge ("APC") for Open Access articles. Where the Submitting Author wishes to make the Work available on an Open Access basis (and intends to pay the relevant APC), the terms of reuse of such Open Access shall be governed by a Creative Commons licence – details of these licences and which Creative Commons licence will apply to this Work are set out in our licence referred to above.

Other than as permitted in any relevant BMJ Author's Self Archiving Policies, I confirm this Work has not been accepted for publication elsewhere, is not being considered for publication elsewhere and does not duplicate material already published. I confirm all authors consent to publication of this Work and authorise the granting of this licence.

For peer review only

Inpatient midwifery staffing levels and postpartum readmissions – a retrospective multi-centre longitudinal study

Lesley Turner*, School of Health Sciences, University of Southampton, Southampton, England, SO17 1BJ

*Corresponding author : Lesley Turner
Email lyt1g19@soton.ac.uk

Christina Saville, School of Health Sciences, University of Southampton, Southampton, England

Jane Ball, School of Health Sciences, University of Southampton, Southampton, England

David Culliford, NIHR Applied Research Collaboration Wessex, School of Health Sciences, University of Southampton, Southampton, England

Chiara Dall'ora, School of Health Sciences, University of Southampton, Southampton, England

Jeremy Jones, School of Health Sciences, University of Southampton, Southampton, England

Ellen Kitson-Reynolds, School of Health Sciences, University of Southampton, Southampton, England

Paul Meredith, School of Health Sciences, University of Southampton, Southampton, England

Peter Griffiths, School of Health Sciences, University of Southampton, National Institute for Health Research Applied Research Centre (Wessex), Southampton, England

Keywords : readmission, post-partum, human resource management

Word count - excluding title page, references, figures and tables 3759 words

For peer review only

Inpatient midwifery staffing levels and postpartum readmissions – a retrospective multi-centre longitudinal study

Abstract

Background

Preventing readmission to hospital after giving birth is a key priority, as rates have been rising along with associated costs. There are many contributing factors to readmission, and some are thought to be preventable. Nurse and midwife understaffing has been linked to deficits in care quality. This study explores the relationship between staffing levels and readmission rates in maternity settings.

Methods

We conducted a retrospective longitudinal study using routinely collected individual patient data in three maternity services in England from 2015-2020. Data on admissions, discharges and case-mix were extracted from hospital administration systems. Staffing and workload were calculated in Hours Per Patient day per shift in the first two 12-hour shifts of the index (birth) admission. Postpartum readmissions and staffing exposures for all birthing admissions were entered into a hierarchical multivariable logistic regression model to estimate the odds of readmission when staffing was below the mean level for the maternity service.

Results

64,250 maternal admissions resulted in birth and 2903 mothers were readmitted within 30 days of discharge (4.5%). Absolute levels of staffing ranged between 2.3 and 4.1 individuals per midwife in the three services. Below average midwifery staffing was associated with higher rates of postpartum readmissions within 7 days of discharge (aOR 1.108, 95% CI 1.003, 1.223). The effect was smaller and not statistically significant for readmissions within 30 days of discharge (aOR 1.080, 95% CI 0.994, 1.174). Below average maternity assistant staffing was associated with lower rates of postpartum readmissions (7 days, aOR 0.957, 95% CI 0.867, 1.057; 30 days aOR 0.965, 95% CI 0.887, 1.049, both not statistically significant).

Conclusion

We found evidence that lower than expected midwifery staffing levels is associated with more postpartum readmissions. The nature of the relationship requires further investigation including examining potential mediating factors and reasons for readmission in maternity populations.

For peer review only

List of abbreviations

aOR	Adjusted odds ratio
NHS	National Health Service
HRG	Healthcare Resource Group
SHMI	Standardised Hospital Mortality Indicator
HPPD	Hours Per Patient Day
RM	Registered Midwife
MA	Maternity Assistant
AIC	Akaike information criterion
BIC	Bayesian information criterion

Strengths and Limitations of this study

Longitudinal design with individual level linking of staffing exposure to subsequent readmissions.

Adjustment for case mix, staff groups and organisational level variables using hierarchical multivariable modelling.

Staffing was measured in the first two 12-hour intervals of the index (birth) hospital stay and did not account for the exposure to staffing for the whole hospital stay.

Considers exposure to staffing in the whole maternity service rather than close to women at ward level.

For peer review only

For peer review only - http://bmjopen.bmj.com/site/about/guidelines.xhtml

Background

In many countries of the world the majority of mothers give birth in hospital[1]. Preventing readmission due to potentially avoidable complications is a key priority, not only because of the negative experience and burden on the women affected[2], but because readmissions are costly[3]. Readmission rates are used as an indicator of the quality of care[4], and in some countries providers are held financially accountable for patient outcomes during the first 30 days after discharge for many patient groups[5]. Rates of postpartum readmission vary by country, with reported rates of 1.0% in the USA[6], 1.7% in Sweden[7] and 3.3% in England and Wales[8].

Reports from England and the USA suggest that readmission rates have increased in recent years[8, 9]. This coincides with an increase in caesarean birth rates[10]. In England 4.3% of women having a caesarean birth are readmitted (1 in 23) compared with 2.9% of those having a vaginal birth (1 in 34)[8]. The recent trend towards shorter length of hospital stay and reduced community provision may also contribute to increased readmission rates. However, whilst some research has pointed to an association between reduced length of stay and increased readmission rates [11, 12] this finding is not consistent in the research literature[13, 14]. The most common reasons for readmission include wound complications, post-partum haemorrhage, hypertension, mastitis and thromboembolism [6, 9, 10, 15, 16]. Postpartum readmissions typically occur within the first 14-20 days after discharge[17, 18].

Pressures on staffing within maternity services have been reported globally[19] but the consequences of this are poorly understood. In many areas of health care there is strong evidence that lower nurse staffing levels are associated with reduced quality of care and poorer patient outcomes (e.g. as measured by mortality[20], adverse events[21] and patient satisfaction)[22]. Studies in medical and surgical inpatient settings have found that higher registered nurse staffing levels are associated with reduced readmission rates[23-25]. A scoping review of midwifery and nurse staffing for inpatient maternity services[26] found just two studies which measured maternal readmission in relation to staffing. These studies found that readmission rates were lower when more midwifery staff were employed[27] and the proportion of registered staff increased[28]. More evidence is needed on the relationship between maternity staffing levels, skill mix and readmission rates to understand whether previous findings are replicated and to understand the size of the effect if an association exists.

For peer review only

The aim of this study is to assess the relationship between individual patient-level exposure to staffing and postpartum readmission rates. A longitudinal design was adopted to enable women's exposure to staffing to be aligned to their outcomes, adjusting for individual risk factors.

Methods

We conducted a retrospective longitudinal study using individual patient records in multiple centres[29]. Maternity inpatient stays from 13th April 2015- 29th February 2020 were extracted from the hospital Patient Administration System for 3 Acute National Health Service (NHS) Trusts in England. Data after February 2020 was not studied due to service changes during the covid pandemic. NHS Trusts comprise one or more hospitals operating as a group under a combined management. Two of the Trusts had inpatient services all on one site (antenatal, postnatal and labour ward). The third Trust had two geographical sites, comprising two maternity hospitals.

The available data included all women admitted to maternity wards, including those admitted via Day Assessment triage unit, totalling 113,002 admissions. Patient data was extracted for the date and time of admissions and discharges per maternity patient episode during the study period. The patient pseudonyms and dates were used to identify which postpartum women had been readmitted to the same maternity service for any cause within 7 days and 30 days of discharge. The primary reason for readmission was extracted from the ICD-10 code for the readmission episode. To account for variation in case mix and comorbidities we used the age band on admission, mode of birth from the Healthcare Resource Group (HRG) procedure code[30], and the Standardised Hospital Mortality Indicator (SHMI) which calculates an age specific risk score based on comorbidities for a given reason for admission ('diagnosis')[31, 32].HRGs are standard groupings of clinically similar treatments which use comparable levels of healthcare resource. Admissions without a birth were excluded from the final data set as they would not be eligible to have a postpartum readmission.

Shift-level staffing data was obtained from Trust electronic roster systems which recorded all worked shifts and the grades of staff. This data comprised the time worked excluding breaks for the Registered Midwife group and Maternity Assistant Staff group and was matched to 12 hour periods, either 07.00 to 19.00 or 1900 to 07.00 to determine the staff time available in each time period. Staffing was measured by Hours Per Patient Day (HPPD) and this variable was generated by dividing the total worked time for the Staff group by the total ward occupancy as detailed in the measure

For peer review only - http://bmjopen.bmj.com/site/about/guidelines.xhtml

guidance document[33].. All admitted individuals were included in the occupancy measure because they all contribute to workload, with neonates accounting for 40% of the admitted population. The HPPD was calculated for Registered Midwife (RM) and Maternity Assistant (MA) staff separately, and the staff groups were combined as a measure of Overall staffing per 12-hour period per day. Skill mix was calculated per shift using the RM worked seconds as a proportion of the total worked seconds, which encompassed RMs and MAs. Registered Nurses were included in the Registered Midwife totals as there were very few (<0.1%) and were of a similar grade to midwives. Data was cleaned to remove shifts where patient occupancy is zero seconds, and to remove shifts with RM HPPD outliers (defined as RM HPPD < 0.5 or RM HPPD > 48).

Staffing for only the index (birth) admission prior to the postpartum readmission was recorded. For the analysis, staffing was noted in the first two 12-hour periods of care in the Index admission (one interval of day shift and one of night shift). This method avoids recording staffing exposure for increased length of stay for a minority of complex cases. Longer time periods will skew the analysis as there is more opportunity to be exposed to understaffing. We defined the expected staffing as the mean HPPD for the maternity service in each Trust, and this was calculated separately for both day and night shifts. Each of the two 12-hour intervals of observed staffing (HPPD) was divided by expected staffing for the same period (HPPD) and these ratios were averaged for the first two 12 hour intervals, with values below 1 indicating a reduction in staff compared with expected levels. The HPPD was converted into the number individuals cared for per midwife and individuals per maternity assistant by dividing 24 hours by the HPPD for each group. This allows service planners to see the potential differences of altering staffing levels in absolute terms using variables familiar to them. A further measure of ward activity and workload was calculated. The variable 'turnover' equalled the total number of admissions plus discharges per Trust per 12 hour interval. Transfers between wards were not included in this measure. Mean turnover was calculated per Trust and variables were created to represent higher than expected turnover compared to the Trust mean, by day and night shifts.

Univariable analyses on the relationship between independent variables and readmissions were performed, nested in hospital Trust. Multi-level logistic regression was then undertaken with the primary outcomes of postpartum readmission within 7 days and 30 days of discharge, nested in hospital Trust. Readmissions within 7 days was included as this may be more sensitive to variation in care quality during index admissions[34, 35], and longer time intervals increase the chance that

For peer review only - http://bmjopen.bmj.com/site/about/guidelines.xhtml

readmission may be due to outpatient management[36]. The null model was the starting point and variables were added firstly based on indicators in the literature to suggest a potential effect on readmission (age, mode of birth), then staffing variables of interest (exposure to RM and MA staffing below the mean in the main analysis) and then remaining variables of SHMI risk, skill mix and turnover were added in sequence and only retained in the full model if they improved model fit using Akaike information criterion (AIC) and Bayesian information criterion (BIC) values (see supplement S1 for model fit using this forward selection process). After the model fitting, only age, mode of birth and SHMI risk remained in the full model along with the staffing variables. For ease of interpretation, we repeated the full model using absolute values of staffing expressed as the number of individuals cared for by each midwife and maternity assistant. We performed a secondary analysis comparing low and high levels of staffing compared to near mean staffing, classified as 95% to 105% of the mean, to explore non-linear relationships. As some sub-groups may have higher support needs and thus be more vulnerable to any adverse effects of low staffing, we performed a secondary analysis to explore staffing effects according to mode of birth.

All analyses were conducted in Stata (Release 16. College Station, TX: StataCorp LLC). Ethics permission was gained via the University of Southampton Ethics Committee ERGO 52957 and IRAS 128056. This study was funded by the National Institute for Health Research, Health Services & Delivery Research programme, award 128056.

Patient and Public Involvement

This study had service user and clinical staff consultation when selecting the outcome measures of interest, and a lay member contributed to the project steering group.

For peer review only - http://bmjopen.bmj.com/site/about/guidelines.xhtml

Results

There were 64,250 maternal admissions that led to a birth between mid-April 2015 and the end of February 2020. The Trusts differed in size with 5,260, 45,518 and 13,472 admissions leading to birth in each of the Trusts respectively. In the cohort, 58.1% of women had a spontaneous vaginal birth, 12.9% assisted instrumental birth, 12.3% planned caesarean birth and 16.7% emergency caesarean birth (Table 1).

Table 1 : Characteristics of the study population and their exposure to staffing

Age		
<20 years	2099	(3.27%)
20-24 years	9,862	(15.35%)
25-29 years	18,362	(28.58%)
30-34 years	20,114	(31.31%)
35-39 years	11,016	(17.15%)
40+ years	2,797	(4.35%)
Mode of Birth		
Assisted birth	7,764	(12.88%)
Emergency Caesarean birth	10,041	(16.66%)
Spontaneous vaginal birth	35,040	(58.14%)
Planned Caesarean birth	7,425	(12.32%)
SHMI	Mean	0.0035
	Std. Dev.	0.0319
Turnover (admissions plus discharges per day)	Mean	56.716
	Std. Dev.	25.397
Registered midwife staffing exposure (HPPD)	Mean	6.640
	Std. Dev.	2.351
Maternity assistant staffing exposure (HPPD)	Mean	2.146
	Std. Dev.	1.045
Skill mix (registered midwives as proportion of total staff)	Mean	0.757
	Std. Dev.	0.039
Readmitted within 30 days of discharge	2903	(4.52%)
Readmitted within 7 days of discharge	2043	(3.18%)

SHMI Standardised Hospital Mortality Indicator HPPD Hours Per Patient Day

For peer review only

Two thousand nine hundred and three women who gave birth were readmitted postnatally within 30 days of discharge (4.5%). Of those readmitted within 30 days, 70% were readmitted within the first 7 days and 89% within 14 days. Rates of readmission differed by birth method: 3.7% of women having spontaneous vaginal birth were readmitted, and rates were 5.9% for planned caesarean birth, 6.3% for assisted birth and 6.8% for emergency caesarean birth respectively. The postpartum readmission rates following birth varied by Trust and were 1.8%, 4.9% and 4.3%. The mean length of stay for postpartum readmissions was 45.4 hours (median 22.5 hrs, IQR 3 hrs to 52 hrs). Infection/sepsis was the most common primary reason for readmission (677/2903, 23.3%). Postpartum haemorrhage accounted for 316/2903 readmissions (10.9%). Some readmissions

For peer review only - http://bmjopen.bmj.com/site/about/guidelines.xhtml

appeared to be healthy mothers accompanying their newborn, although this represented only 125/2903 (4.3%) of maternal readmissions (supplement S2).

Staffing varied by Trust for those in our study cohort, with an average of 2.3, 3.6 and 4.1 individuals per midwife in the three services (this equates to 11.7, 6.9 and 5.9 HPPD respectively). More staff were available on day shifts compared with night shifts. The proportion of RM hours per Trust varies from 70.7%% to 77.4% between the Trusts, and almost all the registered staff are classified as midwives where this data is available (supplements S3-S5).

For two of the Trusts the mean overall staffing in HPPD has shown an increase during the 5-year study period, whereas in the third Trust there is a stable level of overall staffing but a slight decrease in RM staff and increase in MA staffing over this period (approx. 4% change in each group). There was more ward activity in terms of admissions and discharges on day shifts compared with night shifts (supplements S6-S7).

Univariable analyses on covariates and postpartum readmission

Age and the mode of birth were statistically significant predictors of postpartum readmission in univariable analyses, with older mothers and those having assisted or operative birth at higher risk (see Table 2 and supplement S8). Higher SHMI risk was associated with higher risk of readmission, but this was not statistically significant. Women exposed to below average RM staffing and overall staffing had increased odds for readmission, but this was not statistically significant. Having more admissions and discharges than expected and a more diluted skill mix than average was not associated with increased postpartum readmissions at 7 days and 30 days.

For peer review only

For peer review only - http://bmjopen.bmj.com/site/about/guidelines.xhtml

Table 2: Univariable analyses of Readmission at 7 days and at 30 days from discharge

	Readmitted within 7 days			Readmitted within 30 days		
	OR	95% CI		OR	95% CI	
Age category						
<20 years	1.009	0.770	1.323	0.994	0.787	1.256
20-24 years	1.000	reference category		1.000	reference category	
25-29 years	0.954	0.828	1.100	1.013	0.897	1.143
30-34 years	1.040	0.906	1.195	1.095	0.973	1.233
35-39 years	1.043	0.893	1.219	1.125	0.986	1.284
40+ years	1.476	1.193	1.827	1.454	1.208	1.750
Mode of birth						
Assisted birth	1.838	1.622	2.083	1.828	1.642	2.036
Emergency Caesarean birth	1.888	1.685	2.116	2.041	1.854	2.247
Spontaneous vaginal birth	1.000	reference category			reference category	
Planned Caesarean birth	1.500	1.310	1.717	1.706	1.526	1.908
Registered Midwife staffing <mean	1.065	0.974	1.166	1.041	0.965	1.123
Maternity Assistant staffing <mean	0.984	0.898	1.077	0.980	0.908	1.059
Overall staffing <mean	1.054	0.964	1.154	1.041	0.964	1.123
SHMI risk	1.554	0.601	4.021	1.546	0.671	3.560
Turnover > mean	0.972	0.889	1.062	0.966	0.896	1.042
Skill mix <mean	1.017	0.928	1.113	0.979	0.907	1.057

SHMI Standardised Hospital Mortality Indicator

Relationship between staffing and postpartum readmission in multivariable models

Model fit was improved from the null model using a forward selection procedure with the addition of age category, mode of birth, RM and MA staffing and SHMI risk in that order. It was not improved with the addition of turnover and skill mix variables and therefore these variables are not shown in the full model. The variance inflation factors were below 2 for all variables which suggests that they are not collinear. Missing data is minimal for the variables studied (supplement S1).

Lower than expected midwifery staffing was associated with an 11% higher odds of postpartum readmissions within 7 days of discharge (adjusted OR 1.108, 95% CI 1.003, 1.223). The effect was smaller and not statistically significant for readmissions within 30 days of discharge (adjusted OR 1.080, 95% CI 0.994, 1.175). Lower than expected maternity assistant staffing was associated with a lower odds of readmission at 7 days (OR 0.957, 95% CI 0.866, 1.057) and at 30 days (OR 0.965, 95% CI 0.887, 1.049). These relationships were not statistically significant in the multivariable model (see Table 3 and supplement S9).

For peer review only

263 Table 3 : 7 day and 30 day readmissions in the full multivariable models
264 (staffing relative to the mean per service, supplement S9).

Variable	OR readmission 7 days	95% CI		OR readmission 30 days	95% CI	
<20 years	1.066	0.810	1.402	1.065	0.840	1.350
20-24 years	1.000	reference category		1.000	reference category	
25-29 years	0.914	0.790	1.057	0.969	0.856	1.098
30-34 years	0.983	0.853	1.133	1.024	0.906	1.156
35-39 years	0.983	0.837	1.153	1.046	0.913	1.198
40+ years	1.368	1.099	1.703	1.318	1.089	1.594
Assisted birth	1.873	1.648	2.128	1.850	1.657	2.065
Emergency Caesarean birth	1.912	1.701	2.148	2.077	1.883	2.291
Spontaneous vaginal birth	1.000	reference category		1.000	reference category	
Planned Caesarean birth	1.518	1.321	1.745	1.707	1.522	1.915
Exposed to staffing below mean Registered midwives (HPPD)	1.108	1.003	1.223	1.080	0.994	1.174
Exposed to staffing below mean Maternity assistants (HPPD)	0.957	0.866	1.057	0.965	0.887	1.049
SHMI risk	0.687	0.108	4.360	0.8868	0.2421	3.2480

265 SHMI Standardised Hospital Mortality Indicator HPPD Hours Per Patient Day

266

267 In models using absolute (rather than relative) staffing levels, an increase of one individual (mother
268 or baby) per midwife was associated with a 6% increase in the odds of readmission at 7 days,
269 aOR1.063 (95% CI 0.960, 1.177). There was no association with maternity assistant staffing (aOR
270 0.998, 95% CI 0.978, 1.018). Neither relationship was statistically significant (supplement 10)

271

272 Staffing was examined in categories to capture higher than average staffing as well as lower than
273 average levels for readmissions within 7 and 30 days (Table 4, supplement 11). When grouped as
274 three categories, higher midwifery staffing (105% of mean or more) was associated with reduced
275 odds of readmission compared with the category containing the mean. The category of lower than
276 mean midwifery staffing (95% of mean or lower) was associated with increased odds of readmission.
277 None of the relationships in the categorical analyses were statistically significant.

278

For peer review only - http://bmjopen.bmj.com/site/about/guidelines.xhtml

Table 4 : Multivariable analysis with staffing in categories above and below mean levels for readmissions within 7 days and 30 days of discharge

Variable	**OR readmission 7 days**	95% CI		**OR readmission 30 days**	95% CI	
<20 years	1.068	0.812	1.404	1.066	0.841	1.351
20-24 years	1.000	reference category		1.000	reference category	
25-29 years	0.914	0.790	1.057	0.970	0.856	1.099
30-34 years	0.984	0.854	1.134	1.025	0.908	1.158
35-39 years	0.984	0.838	1.154	1.046	0.913	1.199
40+ years	1.370	1.101	1.706	1.319	1.090	1.595
Assisted birth	1.873	1.648	2.127	1.850	1.657	2.065
Emergency Caesarean birth	1.911	1.701	2.147	2.076	1.882	2.290
Spontaneous vaginal birth	1.000	reference category		1.000	reference category	
Planned Caesarean birth	1.518	1.321	1.744	1.705	1.520	1.913
Midwifery staffing						
Low, 95% of mean or less	1.056	0.943	1.182	1.002	0.911	1.102
Near to mean 95%-105%	1.000	reference category		1.000	reference category	
High 105% of mean or more	0.973	0.862	1.098	0.957	0.865	1.060
Maternity assistant staffing						
Low, 95% of mean or less	0.924	0.816	1.046	0.968	0.871	1.076
Near to mean 95%-105%	1.000	reference category		1.000	reference category	
High 105% of mean or more	0.935	0.825	1.059	0.968	0.870	1.077
SHMI risk	0.693	0.111	4.335	0.889	0.243	3.247

SHMI Standardised Hospital Mortality Indicator See supplements S11 and S12

In the secondary analysis by mode of birth, exposure to midwifery understaffing was associated with increased odds of readmission after emergency and planned caesarean births, and assisted births. The estimated effects were stronger in these groups than in the whole cohort (OR 1.113-1.315) although the result was only statistically significant for the assisted birth group. For spontaneous vaginal births there was no association between understaffing and readmissions (supplement S13).

Discussion

In this longitudinal study we analysed individual patient stays and concurrent staff rosters, and we found an association between midwifery staffing below the mean level and higher rates of postpartum readmission. This effect was stronger and statistically significant for readmissions within 7 days of discharge, with an increase in odds of 11% for understaffing compared with the organisation mean staffing levels. We estimate that if a midwife's workload is increased by one additional person, the odds of readmission within 7 days increases by 6.3%, although confidence intervals are wide and cross the null effect in this analysis of absolute staffing data. The direction of findings is in line with that of Gerova[27] who found that increased number of full-time-equivalent midwives in a service was associated with reduced postpartum readmissions. These findings are also seen in the nursing literature, in medical, surgical and paediatric settings[23, 37].

For peer review only - http://bmjopen.bmj.com/site/about/guidelines.xhtml

For peer review only

The mean 30-day readmission rate in this study was 4.5% which is higher than rates reported in USA[6] and Sweden[7] where reports are between 1-2%. This difference may reflect service variability such as community provision and the threshold for readmission[38]. The readmission rate following birth is much lower than with medical and surgical patients[39]. This is likely to be because obstetric patients are healthier and younger than other patient populations and because the index case is often undergoing a normal physiological process rather than management of a disease or condition[5]. The readmission median length of stay was 22.5 hours, which represents a sizeable provision within services each year when multiplied by the rate of readmission.

We found wide variation in the number of individuals cared for by each midwife, from 2.3 per midwife in one organisation to 4.1 per midwife in another. It is important to remember that this figure spans the inpatient maternity service where some women will receive one-to-one care in labour[40] and others will be on wards including neonates with fewer staff per person than in the birthing environment. One of the Trusts had a smaller service than the others, while also having higher rates of staffing and lower readmission rates. Smaller services require more staff to ensure services are available and to manage unscheduled demand[41, 42]. Smaller services may also be treating fewer complex cases which could account for lower readmission rates[38]. These variations were controlled for in our hierarchical analysis and also in the case-mix adjustment, so we can be confident our analysis has taken account of these factors.

Risk factors associated with maternal postpartum readmission include age, ethnicity, parity, body mass index and mode of birth[10, 15]. Individual characteristics were noted to account for most of the inter-hospital variance in postpartum readmission rates in the study by Clapp[43] and we saw further evidence of this as age and mode of birth were statistically significant predictors of readmission in all of our analyses. Staffing is a modifiable factor that has also been associated with rates of readmission[27, 28]. Having more people to care for can affect the quality of care, as some aspects can be missed or incomplete as staff prioritise and ration their time[44]. Some activities such as medicines administration are commonly prioritised, whereas patient teaching and discharge preparation may be seen as less urgent, and therefore more likely to be incomplete or missed[37]. This has been found in a number of studies including those in Malaysia[45], Australia[46], USA[37] and South Korea[47], although not all of these studies are in maternity populations. Our secondary analysis suggested that women with more complex modes of birth are particularly vulnerable to the effects of understaffing.

For peer review only - http://bmjopen.bmj.com/site/about/guidelines.xhtml

The mechanisms for an association between staffing and readmissions need further study. Weiss et al[48] found a sequential pathway from high registered nurse staffing, to improved discharge teaching quality and readiness, to decreased odds of readmission for medical-surgical patients. However, this type of study has not been replicated in the maternity population as far as we are aware. Proposed mechanisms include reduced staff time for recognising deterioration[49], time for education[37], coordination of care[24], hand hygiene compliance[4, 50], discharge planning[44] and medicines optimisation[17]. We found that infection or sepsis was listed as the primary diagnosis in 23% of the postpartum readmissions. Although this was the most common identifiable reason for readmission it was not as high as that reported in the literature, where infection has been implicated in 62% of all postpartum admissions[10] and 82% of readmissions following caesarean birth[16]. Nevertheless, this is still a sizeable figure so mediating pathways from staffing linked to infection and then readmission may be worthy of exploring.

We found a statistically significant association between midwifery staffing below the mean level and higher rates of postpartum readmissions within 7 days of discharge. We went on to look at staffing levels in categories below andabove the mean levels. This categorical analysis of midwifery staffing found fewer readmissions when services were staffed above mean levels and more readmissions when staffed below mean levels, although these were not statistically significant. Service planners should consider setting staffing above mean levels if these findings are confirmed, as it is possible that mean levels in themselves represent suboptimal understaffing.

There was no clear relationship between maternity assistant staffing levels and readmission rates in our study. It may be that maternity assistants perform fewer sensitive activities which could be related to the risk of readmission, although there is very little published evidence on assistants in maternity services to compare our findings to.

Strengths and Limitations

There is limited evidence to determine optimal staffing levels in maternity services. Our research adds to the body of evidence by highlighting the association between staffing levels of midwives and rates of postpartum readmissions. Our analysis has taken account of case mix and between-hospital differences when estimating effects, but unfortunately, we were unable to adjust for other important factors such as ethnicity, parity, deprivation and body mass index from the available data.

For peer review only - http://bmjopen.bmj.com/site/about/guidelines.xhtml

A strength of our approach is the longitudinal and granular design, so we have been able to measure the individual's exposure to staffing per shift, rather than estimating via monthly or annual data. Our approach has an advantage over sequential panel studies as the longitudinal design allows direct individual-level linking of staffing exposure to readmission despite the time lag between these variables.

The amount of missing data is minimal, and therefore we believe this does not materially affect the conclusions drawn. A limitation of our study is that we have been unable to extract information on the staffing close to each woman at the ward level, as two of the Trusts only had staffing data available as a whole service. We measured staffing in the first 24 hours of the admission including birth and will not have captured the hours before discharge for all women. Some of the protective mechanism of adequate staffing could be related to the period prior to discharge, with the handover of care and provision of discharge information. Also we have been unable to account for post-discharge care in the community, which is likely to have a bearing on readmission rates if women have differing opportunities to seek early intervention elsewhere[15]. A further limitation is that our study did not include the rosters of medical staff due to differences in roster configurations. If this staff group is included in future models this may modify the estimates seen for midwives and maternity assistants.

Areas for future research

Any measurable reduction in hospital readmission rates would offer significant financial benefits to hospitals[5]. The cost of increasing staffing could be offset by the potential savings in the future. Cost-effectiveness studies are needed to investigate the consequences of different staffing configurations in relation to the quality of care and related costs and savings. In future research, the components of services that help to reduce readmissions could be explored so resources are applied in areas known to be effective. This may mean targeting increased attention towards at-risk populations that account for a high proportion of readmissions[10, 18]. The provision of additional staff alone may not be fully effective in reducing readmissions unless they are focusing activity on areas of need or critical activities. A more thorough investigation of reasons for readmission would need to be included in this future work.

Conclusion

For peer review only - http://bmjopen.bmj.com/site/about/guidelines.xhtml

Low midwifery staffing in maternity units is associated with increased rates of readmissions within seven days after discharge but there was no adverse effect from lower assistant staffing. This is an important finding especially as readmission rates have increased in some countries in recent years, they are costly to services and distressing for families. Limiting the number of individuals cared for per midwife may have benefits beyond the inpatient stay and result in cost savings if readmissions can be reduced.

Contributor statement

Funding acquisition : PG, CDO, JB, DC, JJ, PM, CS.
CS and PM cleaned and linked data from different sources
LT, PG, CDO, JB, DC, JJ, PM, and CS developed the methodology.
LT conducted the investigation and analysis and wrote the original draft.
LT, PG, CDO, JB, DC, PM, CS and EKR reviewed the results and contributed to interpretation.
PG, EKR and JB supervised this project.
DC provided statistical advice.
All authors reviewed the manuscript.

Acknowledgements.
The views expressed are those of the author(s) and not necessarily those of the National Institute for Health Research, the Department of Health and Social Care, 'arms-length' bodies or other government departments.
Frankie Lambert (FL) made contributions to the work that fall short of ICMJE authorship criteria but we wish her contribution to be recognised.
Please note that, for the purpose of open access, the author has applied a Creative Commons attribution license (CC BY) to any Author Accepted Manuscript version arising from this submission.

Competing interest

No authors have declared any conflicts of interest

Funding

National Institute for Health Research Health Services & Delivery Research programme, award 128056
NIHR Applied Research Collaboration Wessex, no award number.

Data sharing statement

Due to the sensitive nature of the data and data sharing agreements with the providers we are unable to freely share the source data, but we guarantee its authenticity and the rigour of methods used in the analysis.

Ethics approval statement

This work has been reviewed and approved by the following ethics committees ERGO 52957, IRAS 128056.

For peer review only - http://bmjopen.bmj.com/site/about/guidelines.xhtml

For peer review only

For peer review only - http://bmjopen.bmj.com/site/about/guidelines.xhtml

References

1. World Health Organization, Global Health Observatory data repository, 2023. https://www.who.int/data/gho.

2. M.M. Feenstra, I. Nilsson, D.B. Danbjørg, Broken expectations of early motherhood: Mothers' experiences of early discharge after birth and readmission of their infants, Journal of clinical nursing 28(5-6) (2019) 870-881.

3. J.R. Vest, L.D. Gamm, B.A. Oxford, M.I. Gonzalez, K.M. Slawson, Determinants of preventable readmissions in the United States: a systematic review, Implementation science 5(1) (2010) 1-28.

4. K.S. Blume, K. Dietermann, U. Kirchner-Heklau, V. Winter, S. Fleischer, L.M. Kreidl, G. Meyer, J. Schreyögg, Staffing levels and nursing-sensitive patient outcomes: Umbrella review and qualitative study, Health Services Research 00 (2021) 1-23.

5. T.L. Brown, Nurse Staffing, Satisfaction and Hospital Readmission Rates in Skilled Nursing Facilities: A Quantitative, Correlational Study. Doctoral Thesis., University of Phoenix, 2020.

6. M.A. Clapp, S.E. Little, J. Zheng, A.J. Kaimal, J.N. Robinson, Hospital-level variation in postpartum readmissions, JAMA 317(20) (2017) 2128-2129.

7. L. Ellberg, B. Lundman, M. Persson, U. Hogberg, Comparison of health care utilization of postnatal programs in Sweden, Journal of Obstetric, Gynecologic, & Neonatal Nursing 34(1) (2005) 55-62.

8. National Maternity and Perinatal Audit, Clinical Report 2022. Based on births in NHS maternity services in England and Wales between 1 April 2018 and 31 March 2019, 2022. https://maternityaudit.org.uk/FilesUploaded/Ref%20336%20NMPA%20Clinical%20Report_2022.pdf .

9. M.A. Clapp, S.E. Little, J. Zheng, J.N. Robinson, A multi-state analysis of postpartum readmissions in the United States, American journal of obstetrics and gynecology 215(1) (2016) 113. e1.

10. M. Sharvit, T. Rubinstein, D. Ravid, G. Shechter-Maor, A. Fishman, T. Biron-Shental, Patients with high-risk pregnancies and complicated deliveries have an increased risk of maternal postpartum readmissions, Archives of gynecology and obstetrics 290 (2014) 629-633.

11. S. Liu, M. Heaman, M.S. Kramer, K. Demissie, S.W. Wen, S. Marcoux, M.H.S.G.o.t.C.P.S. System, Length of hospital stay, obstetric conditions at childbirth, and maternal readmission: a population-based cohort study, American journal of obstetrics and gynecology 187(3) (2002) 681-687.

12. T. Wen, V.X. Yu, J.D. Wright, D. Goffman, F. Attenello, W.J. Mack, M. D'Alton, A.M. Friedman, Postpartum length of stay and risk for readmission among women with preeclampsia, The Journal of Maternal-Fetal & Neonatal Medicine 33(7) (2020) 1086-1094.

13. J.B. Ford, C.S. Algert, J.M. Morris, C.L. Roberts, Decreasing length of maternal hospital stay is not associated with increased readmission rates, Australian and New Zealand Journal of Public Health 5(36) (2012) 430-434.

14. S. Pervez, N. Sharawi, L. Blake, A. Habib, K. Brookfield, B. Carvalho, Impact of enhanced recovery after caesarean delivery on maternal outcomes: A systematic review and meta-analysis, Anaesthesia, Critical Care & Pain Medicine (100935) (2021) 1-11.

For peer review only - http://bmjopen.bmj.com/site/about/guidelines.xhtml

15. M. Szafrańska, C. Begley, M. Carroll, D. Daly, Factors associated with maternal readmission to hospital, attendance at emergency rooms or visits to general practitioners within three months postpartum, European Journal of Obstetrics & Gynecology and Reproductive Biology 254 (2020) 251-258.

16. S.J. Kipkore, Risk Factors, Indications and Outcomes of Post Cesarean Section Readmission at Kenyatta N. Hospital: a Case-control Study. Masters Thesis., University of Nairobi, 2022.

17. C.A. Combs, D. Goffman, C.M. Pettker, Society for Maternal-Fetal Medicine special statement: a critique of postpartum readmission rate as a quality metric, American Journal of Obstetrics and Gynecology 226(4) (2022) B2-B9.

18. T. Wen, E.E. Overton, J.-J. Sheen, F.J. Attenello, W.J. Mack, M.E. D'Alton, A.M. Friedman, Risk for postpartum readmissions and associated complications based on maternal age, The Journal of Maternal-Fetal & Neonatal Medicine 34(9) (2019) 1375-1381.

19. United Nations Population Fund, The State of The World's Midwifery, 2021. https://www.unfpa.org/sites/default/files/resource-pdf/21-038-UNFPA-SoWMY2021-FastFacts-ENv4302.pdf.

20. J. Needleman, Liu, L., Shang, J., Larson, E., Association of registered nurse and nursing support staffing with inpatient hospital mortality, BMJ quality & safety 29 (2020) 10-18.

21. R.L. Kane, T.A. Shamliyan, C. Mueller, S. Duval, T.J. Wilt, The association of registered nurse staffing levels and patient outcomes: systematic review and meta-analysis, Medical care (2007) 1195-1204.

22. L.H. Aiken, D.M. Sloane, J. Ball, L. Bruyneel, A.M. Rafferty, P. Griffiths, Patient satisfaction with hospital care and nurses in England: an observational study, BMJ Open 8(1) (2018) e019189.

23. C. Ma, M.D. McHugh, L.H. Aiken, Organization of hospital nursing and 30-day readmissions in Medicare patients undergoing surgery, Medical Care 53(1) (2015) 65-70.

24. M.D. McHugh, J. Berez, D.S. Small, Hospitals with higher nurse staffing had lower odds of readmissions penalties than hospitals with lower staffing, Health Affairs 32(10) (2013) 1740-1747.

25. K.B. Lasater, M. McHugh, P.R. Rosenbaum, L.H. Aiken, H. Smith, J.G. Reiter, B.A. Niknam, A.S. Hill, L.L. Hochman, S. Jain, J.H. Silber, Valuing hospital investments in nursing: multistate matched-cohort study of surgical patients, BMJ Quality & Safety 30(1) (2021) 46-55.

26. L. Turner, P. Griffiths, E. Kitson-Reynolds, Midwifery and nurse staffing of inpatient maternity services – a systematic scoping review of associations with outcomes and quality of care, Midwifery 103 (2021) 103118.

27. V. Gerova, P. Griffiths, S. Jones, D. Bick, The association between midwifery staffing and outcomes in maternity units in England: observational study using routinely collected data, Kings College London, 2010.

28. S.J. Kim, K.T. Han, H.J. Lee, J.A. Kwon, E.C. Park, Positive effects of medical staffing on readmission within 30 days after discharge: a retrospective analysis of obstetrics and gynecology data, European Journal of Public Health 26(6) (2016) 935-939.

For peer review only

For peer review only - http://bmjopen.bmj.com/site/about/guidelines.xhtml

29. P. Griffiths, Dall'Ora, C., Ball, J., Culliford, D., Emmanuel, T., Jones, J., Lambert, F., Meredith, P., Rubbo, B., Schmidt, O., Saville, C. , Consequences, costs and cost-effectiveness of different workforce configurations in English acute hospitals: a longitudinal retrospective study using routinely collected data (protocol), in: A.I. NIHR128056 (Ed.) 2022.

30. NHS England, NHS data model and dictionary, 2023. https://www.datadictionary.nhs.uk/supporting_information/healthcare_resource_group.html.

31. NHS Digital, Summary Hospital-level Mortality Indicator (SHMI), 2023. https://digital.nhs.uk/data-and-information/publications/ci-hub/summary-hospital-level-mortality-indicator-shmi.

32. NHS Digital, Indicator Supporting Documentation. IAP00385 Summary Hospital-level Mortality Indicator (SHMI), 2019. https://www.nice.org.uk/Media/Default/Standards-and-indicators/National-Library-of-Quality-Indicators/IAP00385%20supporting%20documentation.docx.

33. NHS Improvement, Care hours per patient day (CHPPD): guidance for acute and acute specialist trusts, 2021.

34. D.L. Chin, H. Bang, R.N. Manickam, P.S. Romano, Rethinking thirty-day hospital readmissions: shorter intervals might be better indicators of quality of care, Health Affairs 35(10) (2016) 1867-1875.

35. K.L. Graham, E.H. Wilker, M.D. Howell, R.B. Davis, E.R. Marcantonio, Differences between early and late readmissions among patients: a cohort study, Annals of Internal Medicine 162(11) (2015) 741-749.

36. N.I. Goldfield, E.C. McCullough, J.S. Hughes, A.M. Tang, B. Eastman, L.K. Rawlins, R.F. Averill, Identifying potentially preventable readmissions, Health care financing review 30(1) (2008) 75.

37. H.L. Tubbs-Cooley, J.P. Cimiotti, J.H. Silber, D.M. Sloane, L.H. Aiken, An observational study of nurse staffing ratios and hospital readmission among children admitted for common conditions, BMJ Quality & Safety 22(9) (2013) 735-742.

38. C. Fischer, H.F. Lingsma, P.J. Marang-van de Mheen, D.S. Kringos, N.S. Klazinga, E.W. Steyerberg, Is the readmission rate a valid quality indicator? A review of the evidence, PloS one 9(11) (2014) e112282.

39. NHS Digital, Emergency readmissions to hospital within 30 days of discharge by diagnosis, 2022. https://digital.nhs.uk/data-and-information/publications/statistical/compendium-emergency-readmissions/current/emergency-readmissions-to-hospital-within-30-days-of-discharge.

40. Care Quality Commission, Maternity survey 2021, 2022.

41. L. Vaughan, N. Edwards, The problems of smaller, rural and remote hospitals: separating facts from fiction, Future Healthcare Journal 7(1) (2020) 38.

42. N. Edwards, Vaughan, L., Palmer, B., Maternity services in smaller hospitals: a call to action, 2020. https://www.nuffieldtrust.org.uk/research/maternity-services-in-smaller-hospitals-a-call-to-action.

43. M.A. Clapp, S.E. Little, J. Zheng, J.N. Robinson, A.J. Kaimal, The relative effects of patient and hospital factors on postpartum readmissions, Journal of Perinatology 38(7) (2018) 804-812.

For peer review only - http://bmjopen.bmj.com/site/about/guidelines.xhtml

44. K.R. Simpson, A. Lyndon, C. Ruhl, Consequences of inadequate staffing include missed care, potential failure to rescue, and job stress and dissatisfaction, Journal of Obstetric, Gynecologic & Neonatal Nursing 45(4) (2016) 481-490.

45. S.T. Nahasaram, V. Ramoo, W.L. Lee, Missed nursing care in the Malaysian context: A cross-sectional study from nurses' perspective, Journal of nursing management 00 (2021) 1-9.

46. R.A. Albsoul, Investigating missed nursing care in an Australian acute care hospital: An exploratory study., Queensland University of Technology, 2019.

47. E. Lee, B.J. Kalisch, Identification and comparison of missed nursing care in the United States and South Korea, Journal of Clinical Nursing 00 (2021) 1-11.

48. M.E. Weiss, O. Yakusheva, K.L. Bobay, Quality and cost analysis of nurse staffing, discharge preparation, and postdischarge utilization, Health services research 46(5) (2011) 1473-1494.

49. L. Jeffs, I. Dhalla, R. Cardoso, C.M. Bell, The perspectives of patients, family members and healthcare professionals on readmissions: preventable or inevitable?, Journal of interprofessional care 28(6) (2014) 507-512.

50. P.J. Rae, S. Pearce, P.J. Greaves, C. Dall'Ora, P. Griffiths, R. Endacott, Outcomes sensitive to critical care nurse staffing levels: A systematic review, Intensive and Critical Care Nursing 67 (2021) 103110.

For peer review only

SUPPLEMENTARY INFORMATION

Table of contents

S1 Model fit and missing data ... 2

1.1 Model fit by Akaike information criterion (AIC) and Bayesian information criterion (BIC) .. 2

1.2 Collinearity testing ... 2

1.3 Missing data per person in the study population (women who had a birth admission) ... 2

S2 Diagnosis codes for the 2903 postpartum readmissions ... 3

S3 Staffing levels expressed as Individuals per Registered Midwife (RM) and Maternity Assistant (MA) ... 5

S4 Staffing on day and night shifts over time in each of the Trusts ... 6

S5 Skill mix of staff within the three Trusts ... 7

S6 Trends in staffing over time in each of the Trusts ... 8

S7 Mean admissions per day and night in each Trust ... 9

S8 Univariable analyses ... 10

S8.1 Age category ... 10

S8.2 Mode of birth ... 11

S8.3 SHMI risk ... 12

S8.4 Understaffing by Registered Midwives, Maternity Assistants and Overall staffing ... 12

S8.5 Turnover (admissions + discharges) more than expected (mean) value for Trust ... 12

S8.6 Skill mix lower than expected (mean) levels for service ... 13

S9 Full models for staffing below the mean compared to mean staffing (HPPD) ... 14

S10. Full models with staffing expressed as individuals per midwife/maternity assistant (absolute level) ... 15

S11 Additional analysis : High and low staffing categories compared with mean staffing ... 16

S12 Frequency of exposure to staffing categories in study population ... 17

S13 Results of full models by subset of population ... 18

S14 Flow chart for data selection ... 19

For peer review only - http://bmjopen.bmj.com/site/about/guidelines.xhtml

SUPPLEMENTARY INFORMATION

S1 Model fit and missing data

1.1 Model fit by Akaike information criterion (AIC) and Bayesian information criterion (BIC)

Postpartum readmission	7 days from discharge model		30 days from discharge model	
	AIC	BIC	AIC	BIC
Empty model nested in Trust	18036.62	18054.76	23539.27	23557.41
Age category added	18031.47	18058.68	23527.22	23554.43
Mode of birth added	17667.94	17703.96	23024.88	23060.91
RM<mean staffing added	17240.87	17285.81	22518.88	22563.81
MA<mean staffing added	17112.23	17166.03	22296.37	22350.17
SHMI added	17008.74	17071.47	22149.12	22211.84
Skill mix added (did not improve model fit so excluded from full model)	17010.53	17082.21	22148.77	22220.45
Turnover added (did not improve model fit so excluded from full model)	17012	17092.65	22149.99	22230.63

RM Registered Midwife, MA Maternity Assistant, SHMI Standardised Hospital Mortality Indicator

1.2 Collinearity testing

Test for collinearity using the Variance inflation factor (VIF)

	VIF
understaffRMless1	1.37
understaffMAless1	1.90
age_category	1.00
Mode_birth	1.00
SHMI_risk	1.00
Higher expected turnover	1.02
Less expected skill mix	1.56

RM Registered Midwife, MA Maternity Assistant

VIF scores are low (<2) so collinearity was not detected. A VIF indicating high collinearity would be >10.

1.3 Missing data per person in the study population (women who had a birth admission)

Age	None
Length of stay	None
Mode birth	None
SHMI	385/64,250 (0.6%)
Staffing Registered midwives	1,258/64,250 (1.96%)
Staffing Maternity Assistants	2,452/64,250 (3.82%)

(Maternity assistant staffing data was missing from Trust A after 28.1.2019)

For peer review only

For peer review only - http://bmjopen.bmj.com/site/about/guidelines.xhtml

SUPPLEMENTARY INFORMATION

S2 Diagnosis codes for the 2903 postpartum readmissions

**ICD codes for primary diagnosis matched to description** Where categories are broad the second diagnosis code has been reported at the end of this table	Count	percentage
Total number of readmissions	**2903**	
Other complications of the puerperium, not elsewhere classified*	482	16.6%
Care and examination of lactating mother**	373	12.8%
Delayed and secondary postpartum haemorrhage	310	10.7%
Puerperal sepsis	246	8.5%
Infection of obstetric surgical wound	227	7.8%
Other specified diseases and conditions complicating pregnancy, childbirth and the puerperium***	159	5.5%
Disruption of caesarean section wound	104	3.6%
Disruption of perineal obstetric wound	103	3.5%
Maternal care for rhesus isoimmunization	71	2.4%
Nonpurulent mastitis associated with childbirth	63	2.2%
Unspecified maternal hypertension	57	2.0%
Haematoma of obstetric wound	45	1.6%
Urinary tract infection following delivery	43	1.5%
Persons encountering health services in other specified circumstances	40	1.4%
Pyrexia of unknown origin following delivery	39	1.3%
Fitting and adjustment of urinary device	33	1.1%
Anaemia complicating pregnancy, childbirth and the puerperium	30	1.0%
Diseases of the digestive system complicating pregnancy, childbirth and the puerperium	26	0.9%
Care and examination immediately after delivery	25	0.9%
Spinal and epidural anaesthesia-induced headache during the puerperium	24	0.8%
Other prophylactic chemotherapy	23	0.8%
Spinal and epidural anaesthesia-induced headache during labour and delivery	21	0.7%
Gestational [pregnancy-induced] hypertension	20	0.7%
Other infection of genital tract following delivery	17	0.6%
Prophylactic immunotherapy	16	0.6%
Other specified pregnancy-related conditions	15	0.5%
Other maternal infectious and parasitic diseases complicating pregnancy, childbirth and the puerperium	14	0.5%
Pre-eclampsia, unspecified	14	0.5%
Retained portions of placenta and membranes, without haemorrhage	13	0.4%
Routine postpartum follow-up	11	0.4%
Supervision of other normal pregnancy	11	0.4%
Other specified puerperal infections	9	0.3%
Diseases of the respiratory system complicating pregnancy, childbirth and the puerperium	8	0.3%
Unspecified infection of urinary tract in pregnancy	8	0.3%
Complication of the puerperium, unspecified	7	0.2%
Diseases of the circulatory system complicating pregnancy, childbirth and the puerperium	7	0.2%
Obstetric blood-clot embolism	7	0.2%
Attention to surgical dressings and sutures	6	0.2%
Elevated blood-pressure reading, without diagnosis of hypertension	6	0.2%
Examination and observation for other specified reasons	6	0.2%

For peer review only - http://bmjopen.bmj.com/site/about/guidelines.xhtml
For peer review only

SUPPLEMENTARY INFORMATION

**ICD codes for primary diagnosis matched to description**<br>Where categories are broad the second diagnosis code has been reported at the end of this table	**Count**	**percentage**
Mental disorders and diseases of the nervous system complicating pregnancy, childbirth and the puerperium	6	0.2%
Other immediate postpartum haemorrhage	6	0.2%
Maternal care due to uterine scar from previous surgery	5	0.2%
Other and unspecified disorders of breast associated with childbirth	5	0.2%
Postpartum coagulation defects	5	0.2%
Second degree perineal laceration during delivery	5	0.2%
Superficial thrombophlebitis in the puerperium	5	0.2%
Unspecified pre-existing hypertension complicating pregnancy, childbirth and the puerperium	5	0.2%
Other conditions with ICD codes affecting <5 women in dataset each (condensed for reporting in this table)	115	4.0%
Blank	7	0.2%

* 68/478 had blank second diagnosis code, 179/478 had ICD indicating a type of pain as second diagnosis code
** 226/373 had blank second diagnosis code, 125/373 had 'Healthy person accompanying sick person' as second diagnosis code
***24/159 had blank second diagnosis code, no pattern seen in remaining ICD codes

When totalling the above 677/2903 (23.3%) of postpartum readmissions were due to infection or sepsis, 316/2903 (10.9%) were due to haemorrhage

Missing ICD diagnosis code for readmissions 7/2903 (0.2%)

For peer review only

SUPPLEMENTARY INFORMATION

S3 Staffing levels expressed as Individuals per Registered Midwife (RM) and Maternity Assistant (MA)

Trust	Mean RM HPPD	Individuals per Midwife	Mean MA HPPD	Individuals per Maternity Assistant
A	11.747	2.330	4.869	5.934
B	5.940	4.104	1.759	14.205
C	6.906	3.576	2.650	9.387
All Trusts (non weighted average)	6.520	3.879	2.146	12.67

RM Registered Midwife, MA Maternity Assistant, HPPD= Hours Per Patient Day

Staffing was calculated for each Trust in the study period and averaged per day.
The 'individuals cared for per staff member' was calculated using the following formula = 24/HPPD.

For peer review only

SUPPLEMENTARY INFORMATION

S4 Staffing on day and night shifts over time in each of the Trusts

Data for staffing for whole admitted population during specified time periods

Year	DAY or NIGHT	Mean RM HPPD	Mean MA HPPD	Mean Overall HPPD
Trust A				
2015	Day	10.75	4.56	15.32
2015	Night	11.10	3.66	14.77
2016	Day	11.73	5.47	17.20
2016	Night	11.76	5.18	16.94
2017	Day	14.22	6.04	20.26
2017	Night	13.54	5.46	19.00
2018	Day	14.08	5.97	20.04
2018	Night	13.81	5.09	18.90
2019	Day	14.68	5.77	19.06
2019	Night	14.28	4.84	17.96
2020	Day	12.57	Not available	Not available
2020	Night	12.44	Not available	Not available
Trust B				
2015	Day	7.00	1.82	8.83
2015	Night	5.09	1.52	6.60
2016	Day	6.93	1.86	8.80
2016	Night	4.99	1.53	6.52
2017	Day	7.19	2.08	9.27
2017	Night	5.04	1.65	6.70
2018	Day	6.71	1.81	8.52
2018	Night	4.81	1.34	6.15
2019	Day	6.92	2.15	9.07
2019	Night	4.89	1.72	6.61
2020	Day	6.61	2.35	8.96
2020	Night	4.84	2.09	6.93
Trust C				
2015	Day	7.05	1.98	9.03
2015	Night	5.82	2.01	7.83
2016	Day	7.02	2.17	9.19
2016	Night	5.91	2.12	8.03
2017	Day	7.23	2.52	9.75
2017	Night	6.06	2.37	8.44
2018	Day	7.34	2.76	10.10
2018	Night	6.16	2.64	8.80
2019	Day	7.91	2.84	10.76
2019	Night	6.96	2.85	9.81
2020	Day	8.62	3.25	11.87
2020	Night	7.84	3.10	10.94

RM = registered midwife MA=maternity assistant HPPD= Hours Per Patient Day

For peer review only - http://bmjopen.bmj.com/site/about/guidelines.xhtml

SUPPLEMENTARY INFORMATION

S5 Skill mix of staff within the three Trusts

This table indicates the skill mix and seniority of staff within Registered Midwife and Maternity Assistant groups.

	Sum of worked hours and % of total in each staff group					
	Total sum of worked hrs	RM Band 6 or above	Registered midwives	Registered nurses	Band 3 care assistants	Band 2 care assistants
Trust A*	316,880	33.5%	70.7%	0.09%	2.2%	26.8%
Trust B	1,990,058	60.4%	77.4%	0%	3.5%	19.1%
Trust C	933,846	59.8%	73.4% (nurses not listed separately)		7.3%	19.2%

*up to 28.1.2019 as care assistant unavailable after this date

The bands refer to NHS Employers Agenda for Change pay scales.
Band 3 is a senior maternity assistant, Band 6 is a senior registered midwife.

For peer review only - http://bmjopen.bmj.com/site/about/guidelines.xhtml

SUPPLEMENTARY INFORMATION

S6 Trends in staffing over time in each of the Trusts

RM = registered midwife MA=maternity assistant HPPD= Hours Per Patient Day

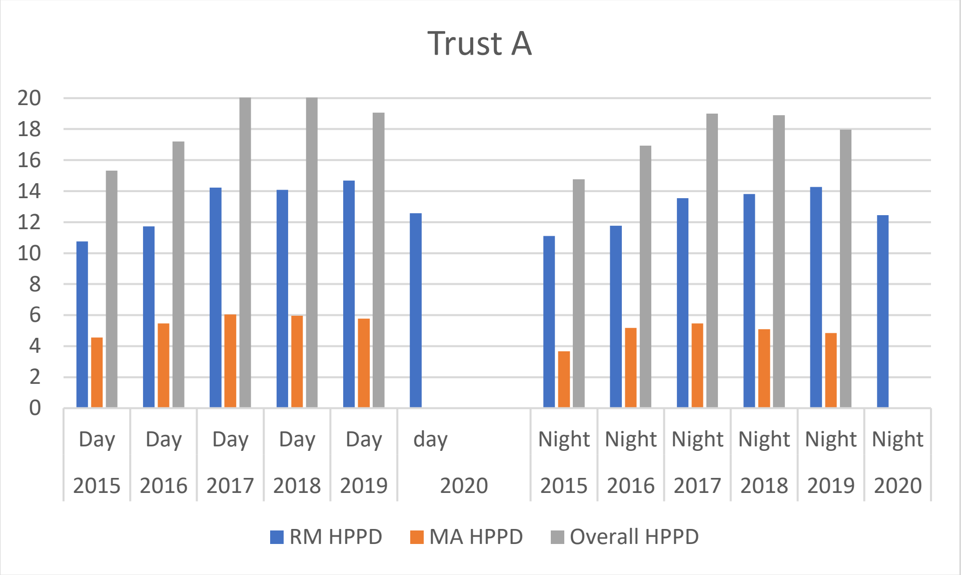

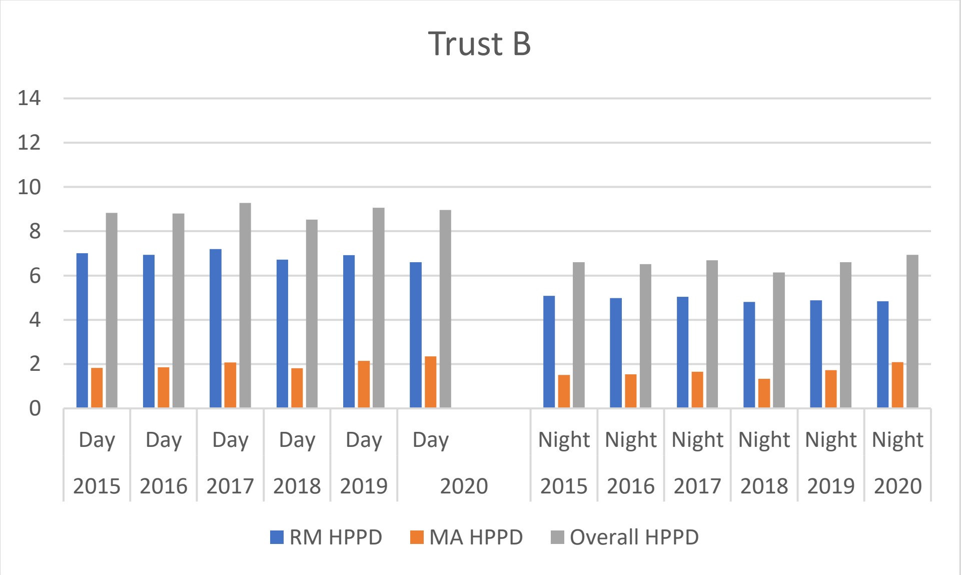

SUPPLEMENTARY INFORMATION

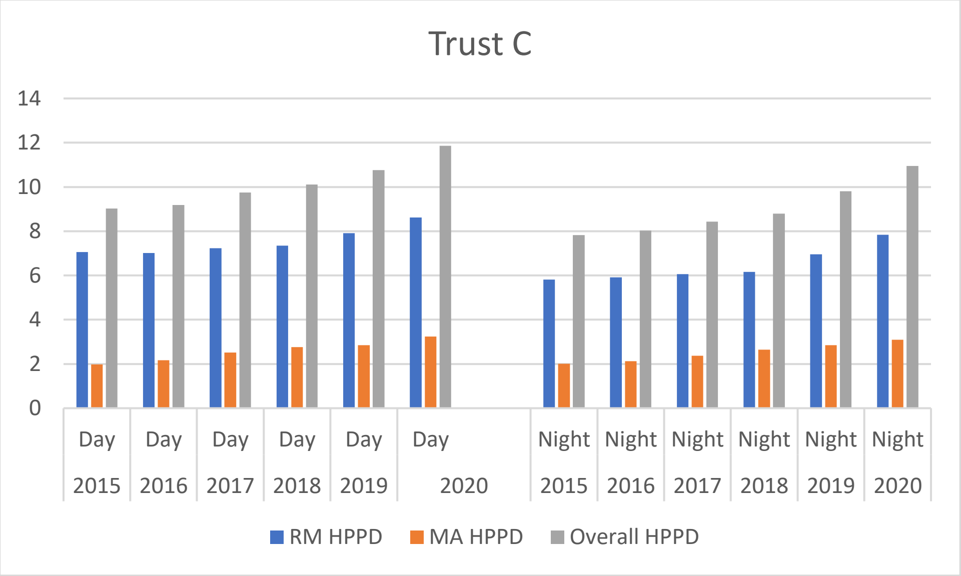

S7 Mean admissions per day and night in each Trust

	Mean admissions per Day	Mean admissions per Night
Trust A	3.2	1.7
Trust B	39.0	28.4
Trust C	15.6	13.5

SUPPLEMENTARY INFORMATION

S8 Univariable analyses

Based on dataset with 64,250 records. Cases leading to birth only and their exposure to staffing and outcomes. Each independent variable was entered into a regression model with only this variable and the readmission outcome variable, nested within hospital Trust.

S8.1 Age category

For outcome of readmission within 7 days
(other age categories are compared with the 20-24 age group)

	Readmitted within 7 days		
	OR	95% CI	
Age category			
<20 years	1.009	0.770	1.323
20-24 years	1.000	reference category	
25-29 years	0.954	0.828	1.100
30-34 years	1.040	0.906	1.195
35-39 years	1.043	0.893	1.219
40+ years	1.476	1.193	1.827

For outcome of readmission within 30 days
(other age categories are compared with the 20-24 age group)

	Readmitted within 30 days		
	OR	95% CI	
Age category			
<20 years	0.994	0.787	1.256
20-24 years	1.000	reference category	
25-29 years	1.013	0.897	1.143
30-34 years	1.095	0.973	1.233
35-39 years	1.125	0.986	1.284
40+ years	1.454	1.208	1.750

For peer review only

SUPPLEMENTARY INFORMATION

S8.2 Mode of birth

For outcome of readmission within 7 days
(other birth categories are compared with spontaneous vaginal birth)

	Readmitted within 7 days		
	OR	95% CI	
Assisted birth	1.838	1.622	2.083
Emergency Caesarean birth	1.888	1.685	2.116
Spontaneous vaginal birth	1.000	reference category	
Planned Caesarean birth	1.500	1.310	1.717

For outcome of readmission within 30 days
(other birth categories are compared with spontaneous vaginal birth)

	Readmitted within 30 days		
	OR	95% CI	
Assisted birth	1.828	1.642	2.036
Emergency Caesarean birth	2.041	1.854	2.247
Spontaneous vaginal birth		reference category	
Planned Caesarean birth	1.706	1.526	1.908

For peer review only

SUPPLEMENTARY INFORMATION

S8.3 SHMI risk

For outcome of readmission within 7 days

	OR	95% CI	
SHMI risk	1.554	0.601	4.021

For outcome of readmission within 30 days

	OR	95% CI	
SHMI risk	1.546	0.671	3.560

S8.4 Understaffing by Registered Midwives, Maternity Assistants and Overall staffing

For outcome of readmission within 7 days

	Readmitted within 7 days		
	OR	95% CI	
Registered Midwife staffing <mean	1.065	0.974	1.166
Maternity Assistant staffing <mean	0.984	0.898	1.077
Overall staffing <mean	1.054	0.964	1.154

For outcome of readmission within 30 days

	Readmitted within 30 days		
	OR	95% CI	
Registered Midwife staffing <mean	1.041	0.965	1.123
Maternity Assistant staffing <mean	0.980	0.908	1.059
Overall staffing <mean	1.041	0.964	1.123

S8.5 Turnover (admissions + discharges) more than expected (mean) value for Trust

For outcome of readmission within 7 days

	Readmitted within 7 days		
	OR	95% CI	
Turnover > mean	0.972	0.889	1.062

For outcome of readmission within 30 days

	Readmitted within 30 days		
	OR	95% CI	
Turnover > mean	0.966	0.896	1.042

For peer review only - http://bmjopen.bmj.com/site/about/guidelines.xhtml

For peer review only

SUPPLEMENTARY INFORMATION

S8.6 Skill mix lower than expected (mean) levels for service

For outcome of readmission within 7 days

	Readmitted within 7 days	
	OR	95% CI
Skill mix <mean	1.017	0.928 1.113

For outcome of readmission within 30 days

	Readmitted within 30 days	
	OR	95% CI
Skill mix <mean	0.979	0.907 1.057

For peer review only

SUPPLEMENTARY INFORMATION

S9 Full models for staffing below the mean compared to mean staffing (HPPD)

For outcome of readmission within 7 days

Variable	OR readmission 7 days	95% CI	
<20 years	1.066	0.810	1.402
20-24 years	1.000	reference category	
25-29 years	0.914	0.790	1.057
30-34 years	0.983	0.853	1.133
35-39 years	0.983	0.837	1.153
40+ years	1.368	1.099	1.703
Assisted birth	1.873	1.648	2.128
Emergency Caesarean birth	1.912	1.701	2.148
Spontaneous vaginal birth	1.000	reference category	
Planned Caesarean birth	1.518	1.321	1.745
Exposed to staffing below mean Registered midwives (HPPD)	1.108	1.003	1.223
Exposed to staffing below mean Maternity assistants (HPPD)	0.957	0.866	1.057
SHMI risk	0.687	0.108	4.360

For outcome of readmission within 30 days

Variable	OR readmission 30 days	95% CI	
<20 years	1.065	0.840	1.350
20-24 years	1.000	reference category	
25-29 years	0.969	0.856	1.098
30-34 years	1.024	0.906	1.156
35-39 years	1.046	0.913	1.198
40+ years	1.318	1.089	1.594
Assisted birth	1.850	1.657	2.065
Emergency Caesarean birth	2.077	1.883	2.291
Spontaneous vaginal birth	1.000	reference category	
Planned Caesarean birth	1.707	1.522	1.915
Exposed to staffing below mean Registered midwives (HPPD)	1.080	0.994	1.174
Exposed to staffing below mean Maternity assistants (HPPD)	0.965	0.887	1.049
SHMI risk	0.8868	0.2421	3.2480

For peer review only - http://bmjopen.bmj.com/site/about/guidelines.xhtml

SUPPLEMENTARY INFORMATION

S10. Full models with staffing expressed as individuals per midwife/maternity assistant (absolute level)

For outcome of readmission within 7 days

Variable	OR readmission 7 days	95% CI	
<20 years	1.066	0.811	1.402
20-24 years	1.000	reference category	
25-29 years	0.913	0.789	1.056
30-34 years	0.983	0.853	1.133
35-39 years	0.983	0.838	1.154
40+ years	1.368	1.099	1.702
Assisted birth	1.873	1.648	2.128
Emergency Caesarean birth	1.912	1.721	2.148
Spontaneous vaginal birth	1.000	reference category	
Planned Caesarean birth	1.519	1.322	1.745
Number of individuals to care for per midwife	1.063	0.960	1.177
Number of individuals to care for per maternity assistant	0.998	0.978	1.018
SHMI risk	0.691	0.109	4.370

For outcome of readmission within 30 days

Variable	OR readmission 30 days	95% CI	
<20 years	1.066	0.840	1.351
20-24 years	1.000	reference category	
25-29 years	0.969	0.856	1.098
30-34 years	1.025	0.907	1.157
35-39 years	1.046	0.913	1.199
40+ years	1.317	1.088	1.593
Assisted birth	1.850	1.657	2.064
Emergency Caesarean birth	2.077	1.883	2.291
Spontaneous vaginal birth	1.000	reference category	
Planned Caesarean birth	1.706	1.520	1.913
Number of individuals to care for per midwife	1.013	0.929	1.105
Number of individuals to care for per maternity assistant	1.002	0.986	1.019
SHMI risk	0.889	0.243	3.255

For peer review only - http://bmjopen.bmj.com/site/about/guidelines.xhtml

SUPPLEMENTARY INFORMATION

S11 Additional analysis : High and low staffing categories compared with mean staffing

Observed (O) and Expected (E) staffing levels were calculated,
with E being the mean for each service.
The ratio of O/E was derived from these figures
The reference category was defined as O/E between 0.95 and 1.05
Low staffing was defined as O/E <0.95
High staffing was defined as O/E >1.05

This grouping allowed the mean (plus 5% either side of the mean) to be considered as an 'expected' level of staffing, allowing for a small amount of variability. The criteria for 'low' and 'high' were set outside this expected level.

This grouping was considered to be more meaningful that splitting the data into tertiles or quantiles or using standard deviations, as it is more reflective of a real clinical scenario and will provide a better insight to service planners.

For outcome of readmission within 7 days

Variable	OR readmission 7 days	95% CI	
<20 years	1.068	0.812	1.404
20-24 years	1.000	reference category	
25-29 years	0.914	0.790	1.057
30-34 years	0.984	0.854	1.134
35-39 years	0.984	0.838	1.154
40+ years	1.370	1.101	1.706
Assisted birth	1.873	1.648	2.127
Emergency Caesarean birth	1.911	1.701	2.147
Spontaneous vaginal birth	1.000	reference category	
Planned Caesarean birth	1.518	1.321	1.744
Midwifery staffing			
Low, 95% of mean or less	1.056	0.943	1.182
Near to mean 95%-105%	1.000	reference category	
High 105% of mean or more	0.973	0.862	1.098
Maternity assistant staffing			
Low, 95% of mean or less	0.924	0.816	1.046
Near to mean 95%-105%	1.000	reference category	
High 105% of mean or more	0.935	0.825	1.059
SHMI risk	0.693	0.111	4.335

SHMI Standardised Hospital Mortality Indicator

For peer review only - http://bmjopen.bmj.com/site/about/guidelines.xhtml

SUPPLEMENTARY INFORMATION

For outcome of readmission within 30 days

Variable	OR readmission 7 days	95% CI
<20 years	1.066	0.841 1.351
20-24 years	1.000	reference category
25-29 years	0.970	0.856 1.099
30-34 years	1.025	0.908 1.158
35-39 years	1.046	0.913 1.199
40+ years	1.319	1.090 1.595
Assisted birth	1.850	1.657 2.065
Emergency Caesarean birth	2.076	1.882 2.290
Spontaneous vaginal birth	1.000	reference category
Planned Caesarean birth	1.705	1.520 1.913
Midwifery staffing		
Low, 95% of mean or less	1.002	0.911 1.102
Near to mean 95%-105%	1.000	reference category
High 105% of mean or more	0.957	0.865 1.060
Maternity assistant staffing		
Low, 95% of mean or less	0.968	0.871 1.076
Near to mean 95%-105%	1.000	reference category
High 105% of mean or more	0.968	0.870 1.077
SHMI risk	0.889	0.243 3.247

SHMI Standardised Hospital Mortality Indicator

S12 Frequency of exposure to staffing categories in study population

For registered midwives the staffing categories breakdown is as follows

1,258/64,250 (1.96%) staffing values known to be missing as reported in supplement 1.3

Staffing caregory	Frequency	percent
Low, 95% of mean or less	24,954	39.61
Near to mean 95%-105%	18,576	29.49
High 105% of mean or more	19,462	30.90

For maternity assistants the staffing categories breakdown is as follows

2,452/64,250 (3.82%) staffing values known to be missing as reported in supplement 1.3

Staffing caregory	Frequency	percent
Low, 95% of mean or less	26,374	42.68
Near to mean 95%-105%	12,520	20.26
High 105% of mean or more	22,903	37.06

For peer review only

For peer review only - http://bmjopen.bmj.com/site/about/guidelines.xhtml

SUPPLEMENTARY INFORMATION

S13 Results of full models by subset of population

Odds of readmission within 7 days, by subset of mode of birth

	Readmission rate	Exposed to staffing below mean Registered midwives (HPPD)	OR 95% CI	Exposed to staffing below mean Registered midwives (HPPD)	OR 95% CI
Whole cohort	3.18%	1.108	1.003, 1.223	0.957	0.866, 1.057
Assisted birth	4.59%	1.315	1.033, 1.675	0.876	0.689, 1.114
Emergency Caesarean	4.67%	1.219	0.991, 1.499	0.893	0.726, 1.098
Spontaneous birth	2.66%	0.983	0.849, 1.139	0.988	0.852, 1.146
Planned Caesarean	3.85%	1.113	0.858, 1.444	1.082	0.833, 1.405

For peer review only

For peer review only - http://bmjopen.bmj.com/site/about/guidelines.xhtml

SUPPLEMENTARY INFORMATION

S14 Flow chart for data selection

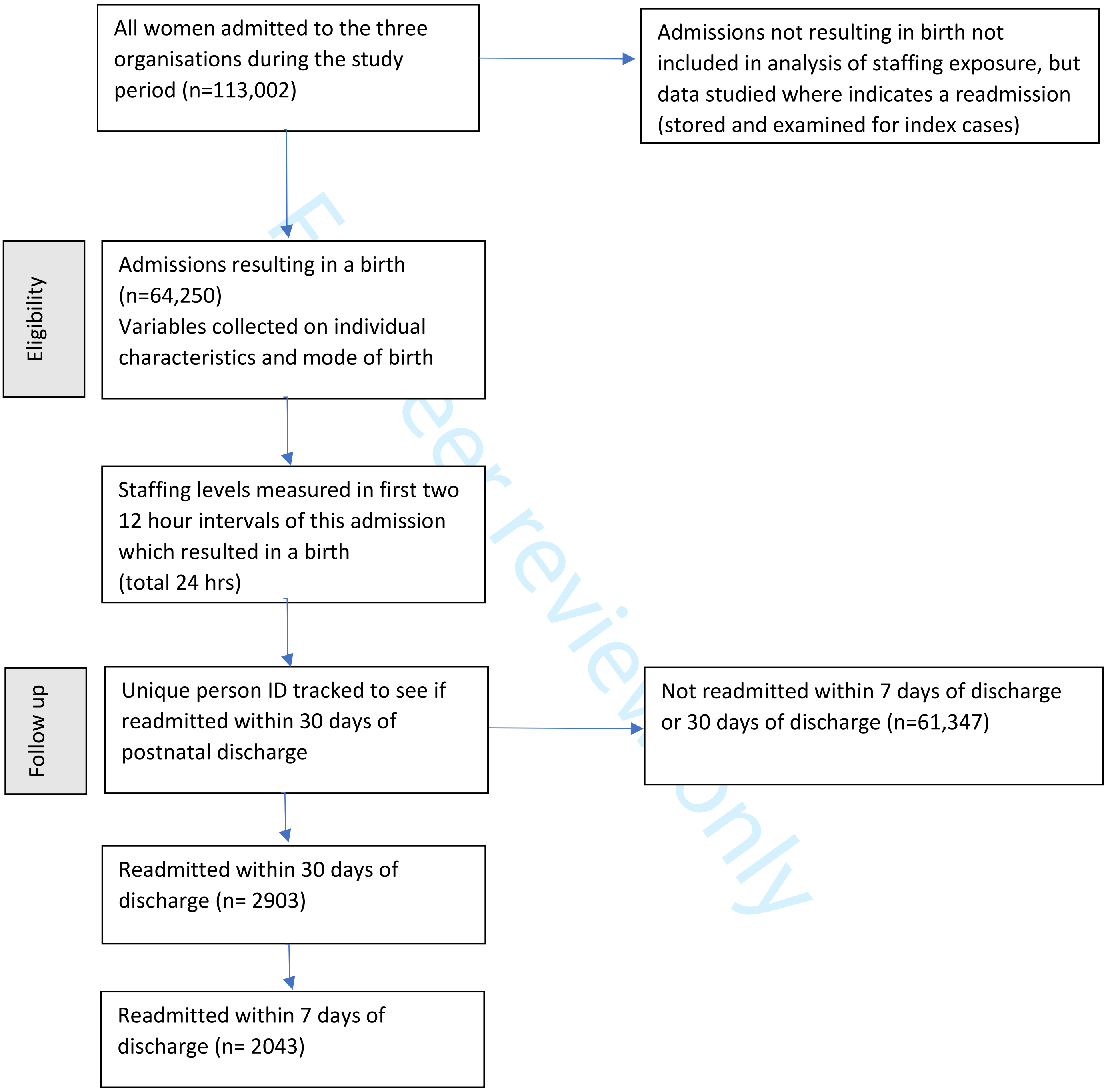

For peer review only - http://bmjopen.bmj.com/site/about/guidelines.xhtml

STROBE Statement—Checklist of items that should be included in reports of ***cohort studies***

	Item No	Recommendation	TRACKED CHANGES DOCUMENT Line No
**Title and abstract**	1	(*a*) Indicate the study's design with a commonly used term in the title or the abstract	1,28
		(*b*) Provide in the abstract an informative and balanced summary of what was done and what was found	30-58
**Introduction**			
Background/rationale	2	Explain the scientific background and rationale for the investigation being reported	82-117
Objectives	3	State specific objectives, including any prespecified hypotheses	118-120
**Methods**			
Study design	4	Present key elements of study design early in the paper	124-125
Setting	5	Describe the setting, locations, and relevant dates, including periods of recruitment, exposure, follow-up, and data collection	125-128
Participants	6	(*a*) Give the eligibility criteria, and the sources and methods of selection of participants. Describe methods of follow-up<br>(*b*) For matched studies, give matching criteria and number of exposed and unexposed	125-136
Variables	7	Clearly define all outcomes, exposures, predictors, potential confounders, and effect modifiers. Give diagnostic criteria, if applicable	136-186
Data sources/ measurement	8*	For each variable of interest, give sources of data and details of methods of assessment (measurement). Describe comparability of assessment methods if there is more than one group	136-186
Bias	9	Describe any efforts to address potential sources of bias	163-167, 190-192
Study size	10	Explain how the study size was arrived at	125-128, supp 13
Quantitative variables	11	Explain how quantitative variables were handled in the analyses. If applicable, describe which groupings were chosen and why	187-203, supp 11
Statistical methods	12	(*a*) Describe all statistical methods, including those used to control for confounding<br>(*b*) Describe any methods used to examine subgroups and interactions<br>(*c*) Explain how missing data were addressed<br>(*d*) If applicable, explain how loss to follow-up was addressed<br>(*e*) Describe any sensitivity analyses	187-206 supp 1, supp 10,11
**Results**			
Participants	13*	(a) Report numbers of individuals at each stage of study—eg numbers potentially eligible, examined for eligibility, confirmed eligible, included in the study, completing follow-up, and analysed<br>(b) Give reasons for non-participation at each stage<br>(c) Consider use of a flow diagram	214-216, supp 13
Descriptive data	14*	(a) Give characteristics of study participants (eg demographic, clinical, social) and information on exposures and potential confounders	Table 1, 215-222
		(b) Indicate number of participants with missing data for each variable of interest	Supplement1

For peer review only - http://bmjopen.bmj.com/site/about/guidelines.xhtml

		(c) Summarise follow-up time (eg, average and total amount)	188-190
Outcome data	15*	Report numbers of outcome events or summary measures over time	223-228

For peer review only

Main results	16	(*a*) Give unadjusted estimates and, if applicable, confounder-adjusted estimates and their precision (eg, 95% confidence interval). Make clear which confounders were adjusted for and why they were included	Table 3, 246-258
		(*b*) Report category boundaries when continuous variables were categorized	Tables2-4
		(*c*) If relevant, consider translating estimates of relative risk into absolute risk for a meaningful time period	277-280
Other analyses	17	Report other analyses done—eg analyses of subgroups and interactions, and sensitivity analyses	277-288, S11
**Discussion**			
Key results	18	Summarise key results with reference to study objectives	295-301
Limitations	19	Discuss limitations of the study, taking into account sources of potential bias or imprecision. Discuss both direction and magnitude of any potential bias	360-382
Interpretation	20	Give a cautious overall interpretation of results considering objectives, limitations, multiplicity of analyses, results from similar studies, and other relevant evidence	295-304
Generalisability	21	Discuss the generalisability (external validity) of the study results	305-307
**Other information**			
Funding	22	Give the source of funding and the role of the funders for the present study and, if applicable, for the original study on which the present article is based	425-427

*Give information separately for exposed and unexposed groups.

Note: An Explanation and Elaboration article discusses each checklist item and gives methodological background and published examples of transparent reporting. The STROBE checklist is best used in conjunction with this article (freely available on the Web sites of PLoS Medicine at http://www.plosmedicine.org/, Annals of Internal Medicine at http://www.annals.org/, and Epidemiology at http://www.epidem.com/). Information on the STROBE Initiative is available at http://www.strobe-statement.org.

For peer review only
